# Clonal lineage tracing and parallel multiomics profiling reveal transcriptional heterogeneity induced by ARID1A deficiency

**DOI:** 10.1126/sciadv.aed7880

**Published:** 2026-10-07

**Authors:** Kenichi Miyata, Liying Yang, Yuxiao Yang, Kohei Kumegawa, Reo Maruyama

**Affiliations:** ^1^ Division of Cancer Epigenomics, Cancer Institute, Japanese Foundation for Cancer Research, 3-8-31 Ariake, Koto-ku, Tokyo 135-8550, Japan.; ^2^ Cancer Cell Communication Project, NEXT-Ganken Program, Japanese Foundation for Cancer Research, 3-8-31 Ariake, Koto-ku, Tokyo 135-8550, Japan.; ^3^ Cancer Cell Diversity Project, NEXT-Ganken Program, Japanese Foundation for Cancer Research, 3-8-31 Ariake, Koto-ku, Tokyo 135-8550, Japan.

## Abstract

Transcriptional heterogeneity drives malignant progression, yet how it emerges within tumors remains unclear. By combining genetic perturbation and lineage tracing with parallel single-cell multiomics, we tracked the consequences of ARID1A loss within individual clonal lineages. ARID1A deficiency increased transcriptional variability both within and across clones, recurrently giving rise to diverse transcriptional profiles that did not converge on a common, coordinated pattern. This variability was accompanied by broadened chromatin accessibility at normally silenced loci and depended on residual SMARCA4 activity. Clones in which this variability was prominent showed no growth advantage under standard conditions but were preferentially recovered under specific environmental stresses and during lung colonization, a context dependence consistent with bet-hedging. Our findings suggest that loss of an epigenetic regulator can promote tumor evolution by relaxing transcriptional constraints and widening the range of phenotypes accessible to a clonal lineage, rather than by imposing a single fixed phenotype.

## INTRODUCTION

Intratumoral heterogeneity (ITH) in gene expression and cell states is a defining feature of cancer and a major determinant of malignant progression, therapeutic resistance, and recurrence (*1*–*6*). Tumors are increasingly viewed as dynamic systems composed of diverse and evolving cellular states rather than uniform populations of genetically identical cells (*7*–*10*). How this transcriptional heterogeneity arises and how it is organized within tumors remains a central challenge in cancer biology (*3*, *5*, *11*–*16*).

Epigenetic modifiers (EMs) are strong candidates for shaping this heterogeneity (*17*–*21*). Large-scale sequencing has revealed recurrent loss-of-function mutations in chromatin remodelers, histone modifiers, and other chromatin-associated factors across many cancers (*22*). In pancreatic ductal adenocarcinoma (PDAC), inactivating mutations in EMs such as ARID1A, SMARCA4, KDM6A, and SETD2 are frequent (*23*–*28*) and often arise as late events, suggesting that they act less as classical oncogenic drivers than as modulators of tumor phenotype.

Because EMs constrain and coordinate transcriptional programs, their loss is expected to relax transcriptional control and increase variability in gene expression (*17*, *20*, *29*–*31*). However, most analyses to date have relied on bulk or population-level single-cell profiling, which cannot determine how EM loss reshapes heterogeneity within individual clonal lineages. It therefore remains unclear whether EM loss simply increases stochastic transcriptional noise or instead drives defined modes of transcriptional diversification.

Critically, the consequences of EM loss need not be uniform across cells. Individual cancer cells differ in transcriptional state, chromatin landscape, and regulatory context at the time of perturbation (*32*–*34*), so loss of a single regulator may produce divergent outcomes in different cells of an otherwise genetically homogeneous population. Population-level analyses cannot distinguish whether the resulting heterogeneity reflects pre-existing differences, stochastic variation, or divergent responses to a shared perturbation. Resolving these possibilities requires strategies that track the transcriptional consequences of EM loss within defined clonal lineages.

To this end, we developed shPseuMO-Tag (shRNA-equipped pseudo-multiomics and cell tagging), a barcoded short hairpin RNA (shRNA) system that couples gene knockdown with clonal lineage tracing and parallel single-cell multiomic profiling. Applying it to ARID1A in PDAC cells, we found that ARID1A deficiency increased transcriptional variability across many clonal lineages. A recurrent consensus non-negative matrix factorization (cNMF)–derived component termed GEP3 lacked a dominant pathway annotation, and cells with high GEP3 usage showed low mutual similarity, supporting its interpretation as an axis of transcriptional variability rather than a discrete cell state. Across clones, GEP3 usage was associated with chromatin-accessibility variability, and both GEP3 usage and accessibility entropy were reduced by SMARCA4 degradation. Clones with high baseline GEP3 usage were preferentially recovered under selected environmental stress conditions and after lung colonization. Together, these findings suggest that ARID1A loss widens the range of transcriptional phenotypes accessible to a lineage and highlight the value of clone-resolved perturbation for dissecting how epigenetic alterations shape ITH.

## RESULTS

### Loss of EMs promotes transcriptional heterogeneity in pancreatic cancer

Loss-of-function alterations in EMs are frequent across human cancers, including PDAC (*22*, *35*). Given their essential role in maintaining chromatin organization and transcriptional fidelity (*18*), we reasoned that impaired epigenetic regulation—whether through mutation or reduced expression—might contribute to tumor heterogeneity. Analyzing RNA sequencing (RNA-seq) data from The Cancer Genome Atlas (TCGA) pancreatic adenocarcinoma cohort, we quantified transcriptomic diversity across tumors using Shannon entropy (*32*) and principal component–based diversity indices (*36*). The reduced expression of EMs was associated with higher transcriptomic diversity for most of the modifiers tested (209 and 136 of 240 modifiers for the two metrics, respectively; fig. S1, A to D). This broad correlation links reduced EM expression to tumor transcriptional diversity at the population level but cannot establish causality or resolve events within individual tumors, motivating a controlled, clone-resolved perturbation approach.

To test directly whether EM loss promotes transcriptomic heterogeneity, we developed shPseuMO-Tag, an all-in-one DNA barcoding system that couples genetic perturbation and lineage tracing with single-cell multimodal profiling (fig. S2, A and B). We applied it to the PDAC cell lines BxPC3 and HPAFII, targeting four EMs (ARID1A, KDM6A, SETD2, and SMARCA4), chosen because their reduced expression correlates with transcriptomic diversity (fig. S1, C and D) and because they are recurrently inactivated in PDAC and associated with poor prognosis (*23*–*28*). To avoid batch effects, all knockdown conditions across both cell lines were pooled and processed in a single single-cell RNA-seq (scRNA-seq) run (*37*). The knockdown of these modifiers increased transcriptomic heterogeneity, measured by normalized mutual information (NMI) (figs. S1, E to H and fig. S2C; see Materials and Methods) (*4*, *38*). Together with the clinical correlation, these results are consistent with a causal contribution of EM loss to transcriptional diversification in PDAC.

### Clonal lineage tracing and parallel multiomics profiling reveal transcriptional heterogeneity following ARID1A loss

While our initial screen indicated that EM loss promotes transcriptional heterogeneity (fig. S1, A to H), it remained unclear whether a heterogeneous population can emerge from a single cell following loss of an EM. We focused on ARID1A, a key subunit of the SWI/SNF chromatin-remodeling complex frequently inactivated in PDAC (*18*, *39*). Using shPseuMO-Tag targeting ARID1A (two independent shRNAs, ARI3# and ARI4#) or a nontargeting control (SCR2#), we transduced BxPC3 cells and, after single-cell cloning and ∼20 cell divisions, established 28 clonal populations (8 control and 20 ARID1A-knockdown clones; Fig. 1A, fig. S3A, and see text S1). Analyzing multiple independent clones from the same parental population, ARID1A-deficient clones showed broader morphological diversity and more variable and generally reduced growth relative to controls (fig. S3, B and C), indicating that ARID1A deficiency increases phenotypic heterogeneity among independently derived clones.

**Fig. 1.**
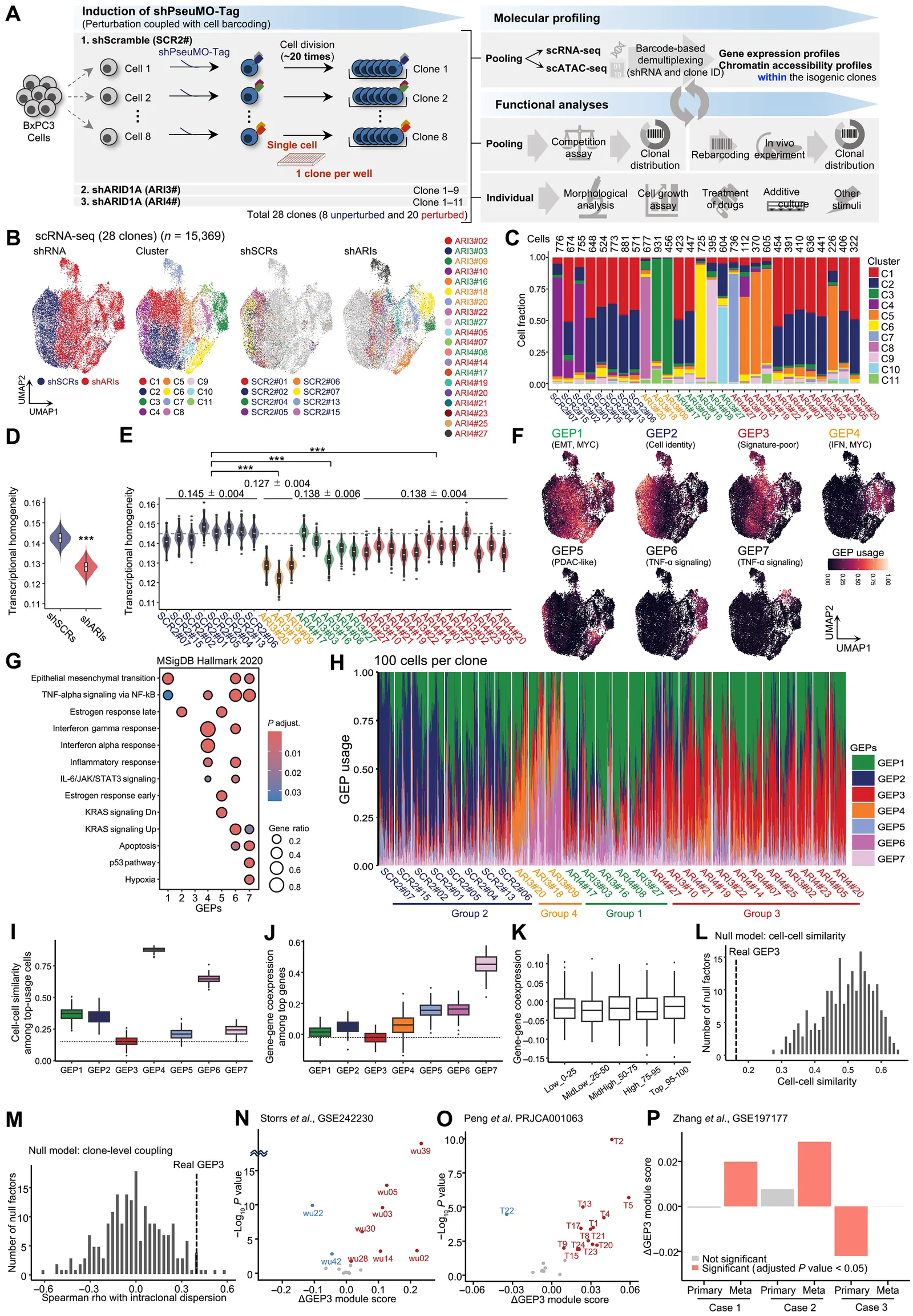
Clonal lineage tracing and parallel multiomics profiling reveal transcriptional heterogeneity following ARID1A loss. (**A**) Clone-resolved workflow. BxPC3 cells were transduced with control or two shARID1A constructs, isolated by single-cell cloning, expanded for approximately 20 divisions, pooled for parallel scRNA-seq and scATAC-seq, with selected clones retained for functional assays. (**B**) UMAP projections of 15,369 scRNA-seq cells from 28 clones, colored by shRNA condition, transcriptomic cluster, or clone identity. (**C**) Fractional distribution of transcriptomic clusters within each clone; numbers indicate assigned cells. (**D** and **E**) NMI-based transcriptional homogeneity for pooled conditions (D) and individual clones (E). Violin plots show bootstrap distributions; labels indicate mean ± SD, and the dashed line indicates the control-clone median. ***P < 0.001, one-way ANOVA with Dunnett’s post hoc test. (**F**) UMAP projections colored by GEP1–GEP7 usage. (**G**) MSigDB Hallmark 2020 enrichment among GEP-associated genes; dot size indicates gene ratio and color indicates adjusted P value. (**H**) GEP usage in 100 sampled cells per clone. (**I**) Cell-cell similarity among the top 5% usage-positive cells for each GEP, calculated from the top 20 associated genes using up to 100 cells in 200 iterations. (**J**) Gene-gene coexpression among the same genes and cells. (**K**) Coexpression among the top 20 GEP3-associated genes across five GEP3-usage percentile bins, with equal sampling over 200 iterations. (**L** and **M**) Null distributions from expression-shuffled NMF for cell-cell similarity (L) and correlations between factor usage and clone-level transcriptional dispersion (M); dashed lines indicate observed GEP3 values. (**N** and **O**) Patient-level ΔGEP3 between ARID1A transcript-negative and -positive PDAC cells in GSE242230 (N) and PRJCA001063 (O), defined as median score in negative minus positive cells; two-sided Wilcoxon rank-sum tests. (**P**) ΔGEP3 in matched primary/metastatic lesions (GSE197177); coral indicates Benjamini-Hochberg–adjusted *P* < 0.05 and gray indicates nonsignificant comparisons.

All 28 clones were profiled by parallel scRNA-seq and scATAC-seq to assess heterogeneity within and across clones (Fig. 1A and see Materials and Methods). scRNA-seq revealed distinct transcriptional shifts in ARID1A-deficient clones relative to controls (Fig. 1, B and C, and fig. S3, D to F), with increased overall transcriptomic diversity (Fig. 1D) and greater variability across individual clones (Fig. 1E). Thus, ARID1A loss facilitates the emergence of diverse transcriptional profiles within individual founder-cell lineages and the divergence of independently derived clones toward distinct profiles.

To dissect these alterations, we applied cNMF (*40*) to the scRNA-seq data. cNMF decomposes expression profiles into gene expression programs (GEPs) and estimates the usage of each GEP in individual cells; this analysis resolved seven GEPs (Fig. 1, F to H, and table S1). Summarizing GEP usage across cells within each clone (pseudo-bulk) and clustering the resulting profiles, all control clones fell into a single group (group 2), enriched for GEP2, a program reflecting baseline BxPC3 identity (*n* = 8; fig. S4, A and B). ARID1A-knockdown clones were instead separated into three groups: group 1 (*n* = 5; GEP1 enriched), group 3 (*n* = 12; GEP3 enriched), and group 4 (*n* = 3; GEP4/6 enriched). ARID1A-knockdown clones thus displayed several distinct transcriptional profiles rather than a single uniform response, with the GEP3-enriched group 3 accounting for the largest fraction of deficient clones. GEP1 corresponded to an EMT-like program and GEP4 to an interferon-like program, both associated with MYC expression (Fig. 1G and fig. S4, C and D).

GEP3 was the most consistently recurring cNMF-derived component across ARID1A-deficient clones, yet unlike the other GEPs, it did not correspond to any dominant pathway signature (Fig. 1G). To characterize this component, we examined its internal structure. Cells with high GEP3 usage were markedly less similar to one another than cells with high usage of coherent programs such as GEP4 (Fig. 1I), and the top-ranked GEP3 genes exhibited comparatively weak coexpression across cells with high GEP3 usage (Fig. 1J). Moreover, gene-module coherence remained low across all GEP3 usage bins, including the highest-usage bin (Fig. 1K), indicating that cells with high GEP3 usage do not converge on a shared transcriptional state. Consistent with this, higher clone-level GEP3 usage tended to be associated with greater intraclonal transcriptional dispersion (fig. S4E). To determine whether these features could arise simply from factorization of data lacking cell-level biological structure, we independently permuted each gene across cells and refit NMF. The observed GEP3 factor exhibited substantially lower cell-cell similarity than factors recovered from the expression-shuffled data (real GEP3, 0.163; null median, 0.503; empirical *P* = 0.0047; Fig. 1L), whereas its coupling to intraclonal transcriptional dispersion was stronger than expected under the shuffled null model (Spearman ρ = 0.398; empirical *P* = 0.033; Fig. 1M). Together, these results indicate that GEP3 does not behave as a conventional coordinated transcriptional program, while its defining properties were not reproduced by factors recovered from expression-shuffled data. Instead, it represents a recurrent, low-coherence, nonrandom axis associated with heterogeneous transcriptional diversification following ARID1A loss (see text S2).

This variability emerged early, increased progressively, and persisted over prolonged culture. GEP3 usage increased and transcriptional homogeneity declined in parallel—consistent with both reflecting the same underlying shift—as early as day 5 after ARID1A knockdown, roughly two to three cell divisions, and these changes became more pronounced by day 16 (fig. S4, F to H). Elevated GEP3 was maintained for at least 10 population doublings, suggesting that this shift was sustained rather than transient (fig. S4I). The same shift occurred in HPAFII cells (fig. S4J). It was also detectable in clinical samples: Across three independent scRNA-seq datasets, GEP3 module scores were higher in ARID1A-negative than in ARID1A-positive cancer cells (Fig. 1, N to P), consistent with the association between ARID1A loss and GEP3 being detectable in clinical samples.

Together, these results show that ARID1A-deficient clones generate divergent transcriptional profiles from single founder cells. Among these, GEP3 stands out as a recurrent, signature-poor axis of transcriptional variability, consistent with a role for ARID1A in restraining transcriptomic diversification.

### ARID1A knockdown remodels the chromatin accessibility landscape

Having found that ARID1A deficiency increases transcriptional variability among clones, we asked whether these transcriptional differences are mirrored in chromatin. From the same pooled population profiled by scRNA-seq (Fig. 1A), we generated parallel single-cell ATAC sequencing (scATAC-seq) data (fig. S5A). Unsupervised clustering resolved 10 chromatin clusters (C1 to C10) (Fig. 2, A and B). Control clones were largely confined to a few clusters, whereas ARID1A-deficient clones were broadly distributed, consistent with extensive chromatin remodeling. Integrating chromatin clusters with GEP-based clone groups, GEP4/6-enriched Group 4 (yellow) mapped mainly to C2; GEP1-enriched group 1 (green) distributed across C1, C2, C5, and C10; and GEP3-enriched group 3 (red) mapped primarily to C3 and C4, two clusters with an increased number of accessible peaks (Fig. 2, A and B, and fig. S5, B and C).

**Fig. 2.**
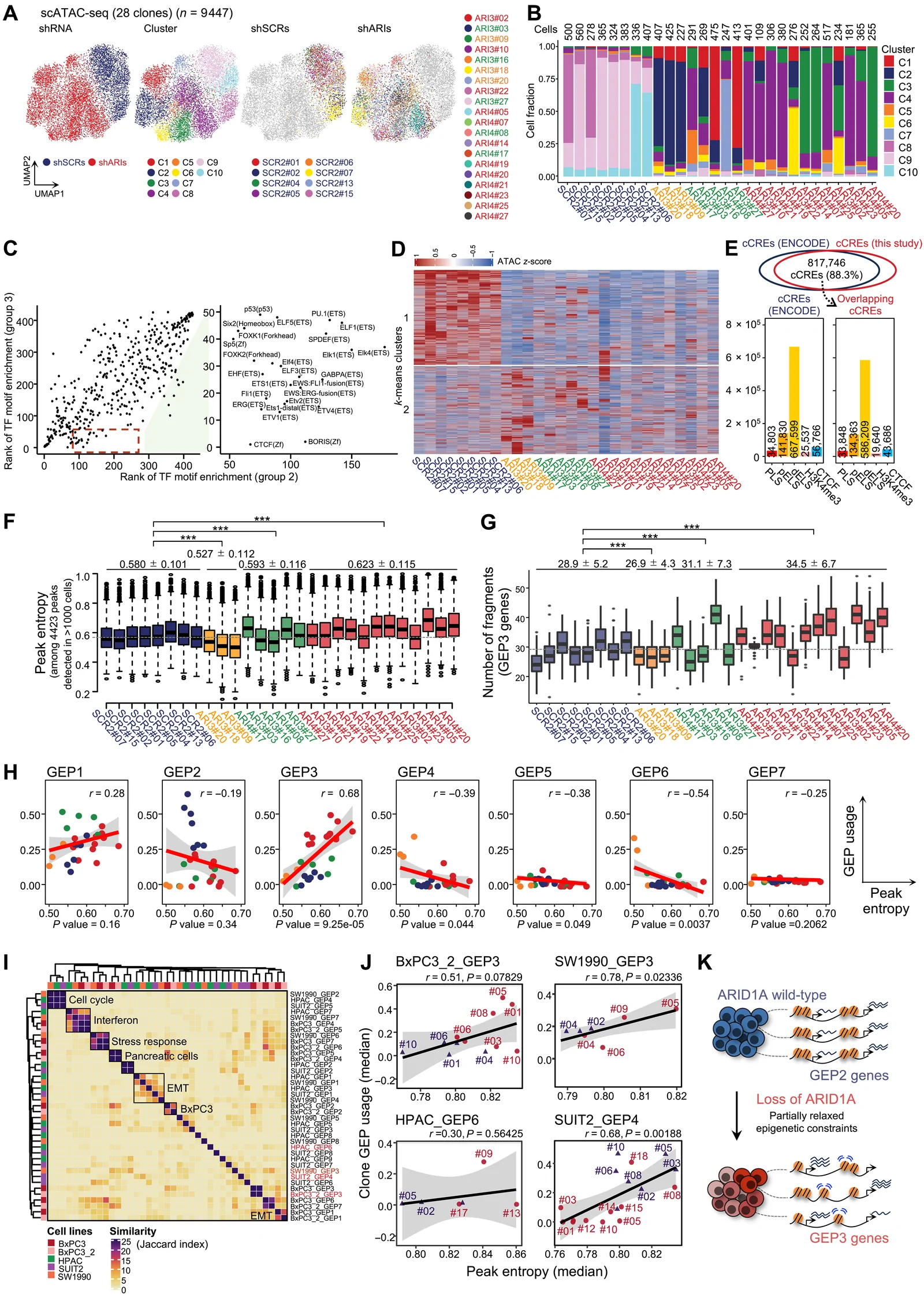
ARID1A loss remodels chromatin accessibility and increases chromatin-accessibility heterogeneity. (**A**) UMAP projections of 9447 scATAC-seq cells from 28 clones, colored by chromatin cluster, shRNA condition, or clone identity. (**B**) Fractional distribution of chromatin clusters within each clone; numbers indicate assigned cells. (**C**) Comparison of transcription-factor motif-enrichment ranks between group 2 (*x* axis) and group 3 (*y* axis), using hypergeometric-test *P* values; the inset highlights group 3 enriched motifs. (**D**) Heatmap of accessibility *z*-scores for 7202 differentially accessible regions (FDR ≤ 0.5 and log_2_ fold change ≥ 0.25) across clones, divided into two *k*-means clusters. (**E**) Overlap between accessible peaks identified here and ENCODE candidate cis-regulatory elements (cCREs): 817,746 of 926,535 cCREs (88.3%) overlapped a study peak. PLS, promoter-like signature; pELS and dELS, proximal/distal enhancer-like signatures; H3K4me3, H3K4me3-associated elements; CTCF, CTCF-only elements. (**F**) Peak-entropy distributions for each clone across 4,423 cCRE-overlapping peaks detected in >1000 cells. Colors denote GEP-based clone groups; labels indicate means ± SD, and the dashed line indicates the control-clone median. ***P < 0.001, one-way ANOVA with Dunnett’s post hoc test. (**G**) ATAC fragments overlapping promoter regions of the top five GEP3-associated genes. Box plots summarize 100 bootstrap samples of 100 cells per clone; annotations and statistics are as in (F). (**H**) Correlations between clone-level median GEP usage and median peak entropy; Pearson correlation coefficients and *P* values are shown. (**I**) Jaccard similarity matrix of cNMF components from the original BxPC3 experiment, BxPC3 replicate, HPAC, SW1990, and SUIT-2 cells based on the top 50 associated genes. (**J**) Correlations between selected signature-poor component usage and peak entropy in BxPC3_2, SW1990, HPAC, and SUIT-2 clones; Pearson coefficients and *P* values are shown. (**K**) Conceptual model in which ARID1A loss partially relaxes epigenetic constraints and increases accessibility at GEP3-associated loci.

Motif enrichment analysis showed a shared gain of accessibility at ETS family and CTCF motifs in ARID1A-deficient clones (Fig. 2C and fig. S5, D and E), but we found no strong enrichment of clone-specific marker peaks (Fig. 2D). This suggests that ARID1A loss does not impose new clone-specific chromatin identities but instead relaxes pre-existing regulatory constraints, yielding a more heterogeneous accessibility landscape. Comparison with ENCODE candidate cis-regulatory elements (cCREs) identified 817,746 overlapping elements (88.3%; Fig. 2E), indicating that the accessible landscape extensively intersected annotated regulatory elements. Nearest-gene annotation of C3/C4-specific accessible peaks overlapping these cCREs identified 1497 genes enriched for normally silenced and lineage-inappropriate loci, without a dominant oncogenic or metastasis-associated pathway (table S2). The entropy analysis of 4423 cCREs confirmed greater cell-to-cell variability in accessibility in ARID1A-deficient clones, with group 3 showing the highest peak entropy (Fig. 2F and fig. S5F).

We next asked whether this chromatin variability tracked with any of the GEPs. Across clones, peak entropy correlated positively with GEP3 usage (*r* = 0.68) but not with the other GEPs (Fig. 2, G and H). Notably, GEP4/6-enriched group 4 clones were also transcriptionally heterogeneous yet showed comparatively low peak entropy, dissociating chromatin-accessibility variability from transcriptional heterogeneity in general and linking it specifically to GEP3. Because GEP3 usage and peak entropy are derived from independent modalities (RNA and chromatin accessibility, respectively), this correspondence indicates that the transcriptional variability captured by GEP3 has a counterpart in chromatin.

To test whether the GEP3-like component was reproducible, we first repeated the entire experiment in BxPC3 as a fully independent replicate and separately applied the same shPseuMO-Tag strategy to four additional PDAC cell lines (HPAC, SW1990, SUIT-2, and HPAFII; fig. S6, A and B; table S3; and see text S3). In the independent BxPC3 replicate, a subset of ARID1A-deficient clones again gave rise to a component with the principal features of GEP3—no dominant Hallmark signature, high peak entropy, and low transcriptional homogeneity (Fig. 2, I and J, and fig. S6, C to F)—recovering the original finding in a separately derived set of clones. In this replicate, the EMT-like and interferon-like programs also recurred but were not restricted to ARID1A-deficient clones and appeared among control clones as well, consistent with ARID1A loss altering access to pre-existing states rather than creating them de novo. Among the additional cell lines, the outcome varied with cellular background (fig. S7): A signature-poor component associated with increased peak entropy was identified in SW1990 and HPAC, although the specific contributing genes differed between lines (Fig. 2, I and J, and fig. S7, A to R) — as expected if what recurs is a mode of transcriptional variability rather than a fixed gene module. Together, these results show that GEP3 is recovered in an independently derived set of BxPC3 clones and suggest that comparable programs can arise in other PDAC backgrounds following ARID1A loss (Fig. 2K).

### GEP3-enriched clones show context-dependent selective advantages

Having characterized GEP3 as a recurrent high-variability component, we asked what phenotypes were associated with high baseline GEP3 usage. We began in vitro, subjecting an equally mixed pool of 12 clones—three each from GEP1-enriched group 1, control group 2, GEP3-enriched group 3, and GEP4/6-enriched group 4—to a panel of tumor microenvironment–related stressors (Fig. 3A). Because control clones proliferate faster under standard conditions (fig. S3C), we measured stress-induced enrichment relative to each clone’s unperturbed baseline. GEP3-enriched group 3 clones were reproducibly enriched under CoCl_2_ (hypoxia-mimetic) and glutamine deprivation, whereas glucose deprivation instead favored an interferon-like and an EMT-like clone (Fig. 3, B and C). Thus, different clone groups were favored under different pressures, with GEP3-enriched clones advantaged under a specific subset—a context-dependent rather than a general fitness benefit.

**Fig. 3.**
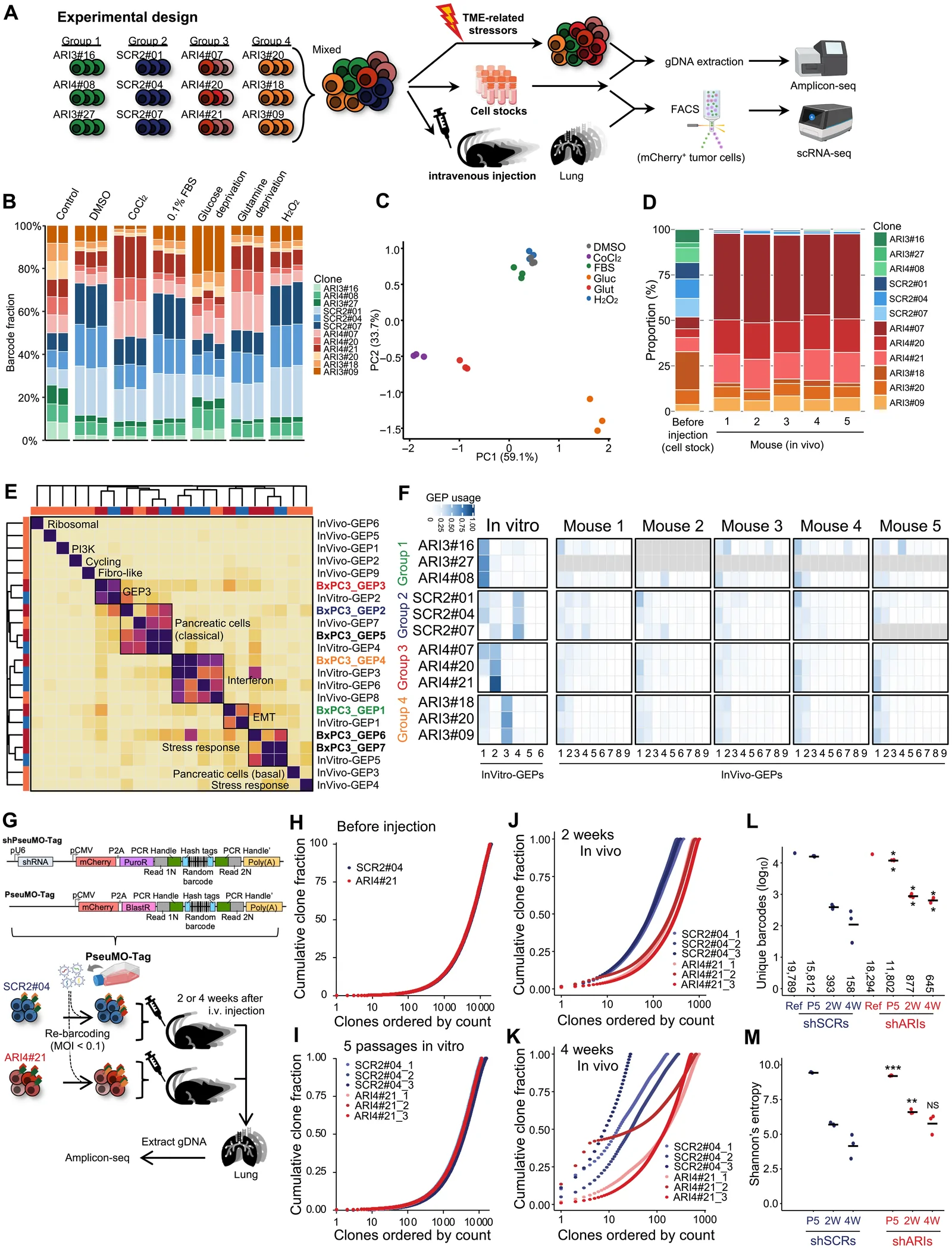
GEP3-enriched clones show context-dependent selective advantages. (**A**) Twelve-clone competition workflow. Three clones per transcriptional group were mixed equally. For in vitro assays, the pool was exposed to microenvironmental stressors and analyzed by amplicon sequencing. For the in vivo assay, the pool was injected intravenously; after 2 weeks, viable mCherry-positive lung tumor cells were isolated by FACS and profiled by scRNA-seq with the matched pre-injection pool. The schematic in (A) was partially created with BioRender content. Created in BioRender. K. Miyata (2026; https://BioRender.com/7hlgqei). (**B**) Clonal composition at baseline, after DMSO, or after exposure to CoCl_2_, 0.1% FBS, glucose deprivation, glutamine deprivation, or H_2_O_2_. (**C**) PCA of clonal abundance profiles from the stress assay; points represent replicates. (**D**) Clonal composition of the matched pre-injection pool and lung-derived samples from five mice. (**E**) Jaccard similarity matrix of cNMF components from the original 28-clone BxPC3 dataset, matched pre-injection dataset, and lung-derived dataset, based on the top 50 associated genes. (**F**) Median component usage per clone before and after lung colonization; gray indicates clone-sample combinations with no recovered cells. (**G**) PseuMO-Tag rebarcoding workflow. SCR2#04 and ARI4#21 were rebarcoded at MOI < 0.1 and analyzed in vitro or after intravenous injection into NSG mice. (**H** and **I**) Cumulative fractions of barcode-defined sublineages before injection (H) and after five in vitro passages (I). (**J** and **K**) Fractions recovered from lungs after 2 weeks (J) and 4 weeks (K). Red indicates ARI4#21-derived sublineages, and blue indicates SCR2#04-derived sublineages. (**L** and **M**) Unique barcode numbers (log_10_) (L) and Shannon entropy of barcode distributions (M) in the reference population, after five passages, and after 2 or 4 weeks in vivo. Points represent biological replicates; horizontal bars indicate medians. **P < 0.01 and ***P < 0.001, one-way ANOVA with Dunnett’s post hoc test; NS, not significant.

We next tested whether this advantage extended to an in vivo setting. Twelve clones—three each from groups 1 to 4—were mixed in equal proportions and injected intravenously into NSG mice, with matched in vitro cultures processed in parallel (Fig. 3A). Two weeks after injection, GEP3-enriched group 3 clones were preferentially recovered from the lung (Fig. 3D and fig. S8A). Because groups 1 and 4 are also ARID1A deficient yet showed no such advantage, enhanced colonization was tracked with high GEP3 usage rather than with ARID1A loss per se. In an independent experiment using a separate set of clones, GEP3-enriched clones similarly outcompeted controls in the lung (fig. S8, B and C), supporting this finding.

To examine how cells from GEP3-enriched clones were transcriptionally organized in this new environment, we compared the GEPs of clones before and after engraftment. In vitro, cNMF recovered a GEP3-like component as a major axis of cell-to-cell variation (InVitro-GEP2); in lung-derived cells, however, this axis was no longer dominant, and cells were instead organized by a distinct adaptive program (InVivo-GEP1), modestly enriched for phosphatidylinositol 3-kinase/AKT/mechanistic target of rapamycin signaling and protein-secretion signatures (Fig. 3, E and F; fig. S8, D to F; and table S4). This did not reflect loss of the GEP3 module signal, which was retained—and modestly increased—in lung-derived group 3 cells (fig. S8G). Rather, the dominant axis of variation shifted: After engraftment, GEP3-enriched populations were organized by InVivo-GEP1 while retaining GEP3 module activity. Notably, the few control clones that engrafted were also organized by InVivo-GEP1, indicating that cells of different clonal origins can adopt a shared lung-associated transcriptional program. Together, these observations suggest that high GEP3 usage does not itself drive colonization but instead marks clones from which environment-specific adaptive programs more readily emerge—a plastic, adaptable population rather than a fixed phenotype.

To examine this at the level of clonal diversity, we used a rebarcoding system that labels the descendants of a single clone with distinct barcodes, allowing sublineage complexity to be tracked through selection (Fig. 3G). A GEP3-enriched clone (ARI4#21) retained greater sublineage diversity than a control clone (SCR2#04) through lung colonization (Fig. 3, H to M), consistent with greater barcode-defined sublineage complexity being maintained within this GEP3-enriched clone.

### Residual SMARCA4 activity sustains GEP3-associated transcriptional and chromatin variability

The chromatin variability associated with GEP3 (Fig. 2H) raised the question of which residual SWI/SNF component sustains these features once ARID1A is lost. To generate hypotheses, we inferred transcriptional regulator activities from the scRNA-seq data across clones (Fig. 4A). Clones stratified largely along their GEP-based groups, and the inferred activity of SMARCA4—the catalytic ATPase of SWI/SNF (*41*)—was elevated in ARID1A-deficient clones, including both GEP3-enriched group 3 and GEP4/6-enriched group 4, relative to controls. This pointed to residual SMARCA4 as a candidate for maintaining GEP3-associated chromatin and transcriptional features, which we tested directly by perturbation.

**Fig. 4.**
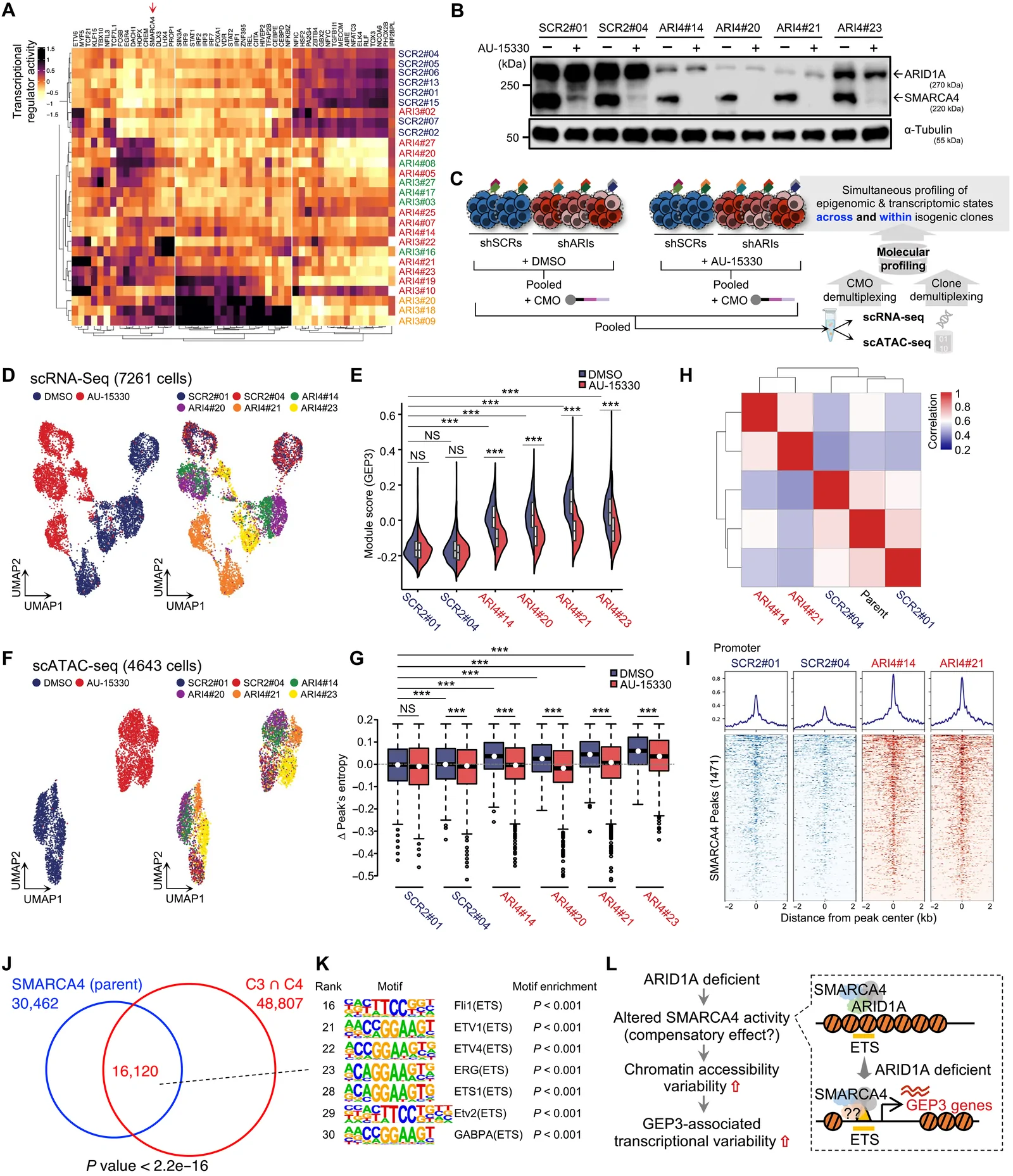
Residual SMARCA4 sustains GEP3-associated transcriptional and chromatin variability after ARID1A loss. (**A**) Heatmap of inferred transcriptional-regulator activities across 28 clones. The 50 regulators with the greatest variability are shown; the arrow indicates SMARCA4. (**B**) Immunoblot analysis of ARID1A, SMARCA4, and α-tubulin in selected clones treated with DMSO or the SMARCA4 degrader AU-15330. (**C**) Workflow for parallel single-cell profiling after SMARCA4 degradation. Control and ARID1A-knockdown clones were treated with DMSO or AU-15330 and pooled within each treatment group. Cell-multiplexing oligos distinguished the two scRNA-seq pools, and PseuMO-Tag barcodes resolved clone identities in both scRNA-seq and scATAC-seq. (**D**) UMAP projections of 7261 scRNA-seq cells, colored by treatment condition or clone identity. (**E**) GEP3 module scores in individual clones treated with DMSO or AU-15330. ***P < 0.001 and NS, not significant, one-way ANOVA with Tukey’s post hoc test. (**F**) UMAP projections of 4643 scATAC-seq cells, colored by treatment or clone. (**G**) Distributions of treatment-induced changes in peak entropy (Δ peak entropy) across clones. ***P < 0.001, one-way ANOVA with Tukey’s post hoc test. (**H**) Pairwise Pearson correlations of SMARCA4 CUT&Tag peak signals across parental cells and selected control and ARID1A-knockdown clones. (**I**) SMARCA4 CUT&Tag profiles at promoter-associated peaks enriched in ARI4#21. Lines show average spike-in-normalized counts per million mapped reads, and heatmaps show signal intensity at individual peaks centered on the summits. (**J**) Overlap between accessible peaks assigned to scATAC-seq clusters C3 and C4 and SMARCA4 CUT&Tag peaks in parental BxPC3 cells. The overlap comprised 16,120 peaks; the hypergeometric test *P* value is indicated. (**K**) ETS-family motif enrichment among the overlapping peaks in (**J**). (**L**) Proposed model in which residual SMARCA4 contributes to chromatin-accessibility variability and GEP3-associated transcriptional variability following ARID1A loss.

We treated control (group 2) and GEP3-enriched (group 3) clones individually with the SMARCA4 degrader and profiled them by parallel scRNA-seq and scATAC-seq (Fig. 4, B and C, and see text S1). SMARCA4 degradation reduced GEP3 usage in Group 3 clones (Fig. 4, D and E, and fig. S9, A to D) and concomitantly decreased peak entropy (Fig. 4, F and G, and fig. S9, E to H). Thus, the elevated transcriptional and chromatin variability associated with GEP3 share a dependence on residual SMARCA4, supporting a common SMARCA4 dependence of the two features correlated in Fig. 2H.

To ask how SMARCA4 acts on these regions, we mapped genome-wide SMARCA4 occupancy (Fig. 4H). SMARCA4 binding was redistributed in ARID1A-deficient clones and became enriched at promoter and exonic regions in Group 3 clones (Fig. 4I and fig. S9, I to L). The accessible peaks that characterize chromatin clusters C3 and C4—where GEP3-enriched clones predominate (Fig. 2, A and B)—overlapped with SMARCA4-bound regions (16,120 of the 48,807 peaks; Fig. 4J), and these shared regions were enriched for ETS-family motifs (Fig. 4K). Together, these results indicate that, following ARID1A loss, SMARCA4 preferentially occupies a set of regulatory regions whose increased and variable accessibility is associated with GEP3 usage and whose enrichment for ETS-family motifs suggests a possible involvement of ETS-family factors (Fig. 4L).

### Large-scale clonal tracing links GEP3 usage to heterogeneity and stress adaptation

To evaluate how EM loss shapes clonal dynamics at scale, we expanded from 28 to 2000 clones (400 per condition across five conditions). BxPC3 cells transduced with shPseuMO-Tag targeting ARID1A, KDM6A, SETD2, or SMARCA4 or a control, were cultured for ∼15 divisions, pooled, and profiled by parallel scRNA-seq and scATAC-seq (Fig. 5A; fig. S10, A to C; and see text S1). Except for KDM6A, EM knockdown increased transcriptomic and/or epigenomic heterogeneity in the pooled populations (Fig. 5, B and C, and fig. S10D). Demultiplexing by PseuMO-Tag barcodes resolved individual clones (fig. S10, E to H): 415 clones were recovered in the control but markedly fewer under knockdown (KDM6A, 249; SETD2, 272; ARID1A, 262; SMARCA4, 60), consistent with clonal imbalance and preferential expansion of specific clones (Fig. 5, D to H, and fig. S10I).

**Fig. 5.**
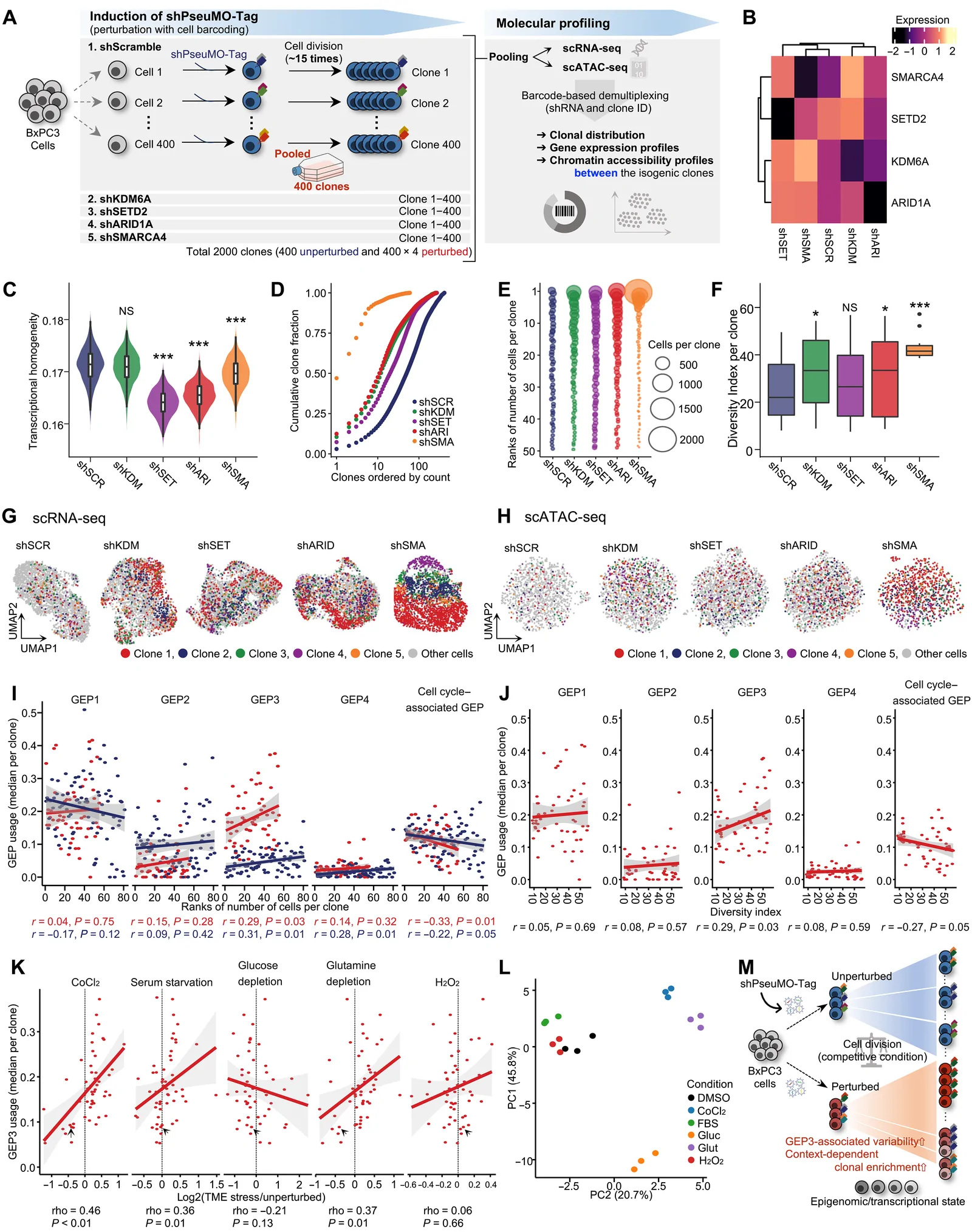
Large-scale clonal tracing links epigenetic-modifier loss to clonal selection and transcriptional diversification. (**A**) Workflow. BxPC3 cells were transduced with shPseuMO-Tag targeting KDM6A, SETD2, ARID1A, or SMARCA4, or with a nontargeting control. Approximately 400 founders per condition were expanded for ∼15 divisions, yielding ∼2000 barcode-defined clones. Populations were pooled within each experiment and profiled by parallel scRNA-seq and scATAC-seq using aliquots of the same mixture. (**B**) Heatmap of target-gene expression across shRNA conditions. (**C**) Transcriptional homogeneity quantified by NMI from 1000 bootstrap samples of 50 cells. ***P < 0.001, one-way ANOVA with Dunnett’s post hoc test. (**D**) Cumulative clone-fraction distributions, with clones ordered by cell count. (**E**) Bubble plot of the 50 most abundant clones per condition, ordered by rank; bubble size indicates assigned cells. (**F**) PCA-based transcriptional diversity calculated per clone and summarized across conditions. ***P < 0.001 and *P < 0.05, one-way ANOVA with Dunnett’s post hoc test. (**G** and **H**) UMAP projections of scRNA-seq (G) and scATAC-seq (H) cells, highlighting the five most abundant clones per condition; other cells are gray. (**I**) Correlations between clonal-abundance rank and clone-level median GEP usage in control (blue) and ARID1A-knockdown (red) clones; Pearson coefficients and *P* values are shown separately. (**J**) Correlations between median GEP usage and PCA-based transcriptional diversity among ARID1A-knockdown clones; Pearson coefficients and *P* values are shown. (**K**) Correlations between baseline clone-level median GEP3 usage and stress-specific enrichment in 54 ARID1A-knockdown clones. Enrichment was calculated as log_2_(stress/unperturbed); Spearman coefficients and *P* values are shown. Arrows indicate ARI#01, the most abundant pooled clone. (**L**) PCA of clonal-abundance profiles from the large-scale environmental-stress assay; points represent replicates. (**M**) Summary model linking ARID1A loss to GEP3-associated transcriptional variability and context-dependent clonal enrichment under environmental stress.

To relate GEP usage to clonal behavior, we applied cNMF to the ARID1A knockdown and control clones, recovering components resembling those defined earlier (Fig. 1F) together with a cell cycle–associated program (table S5). The cell cycle–associated program correlated negatively with clonal dominance (*r* = −0.33, *P* = 0.014; Fig. 5I). GEP3 usage was elevated across the ARID1A-knockdown clone population relative to controls, consistent with ARID1A loss increasing GEP3 usage at scale. GEP3 usage also correlated positively with clone-level transcriptional diversity (*r* = 0.29, *P* = 0.03; Fig. 5J), consistent with its association with ARID1A loss–induced heterogeneity. Notably, GEP3 usage showed a modest positive correlation with clonal dominance in this competitive setting (*r* = 0.29, *P* = 0.029; Fig. 5I). We therefore examined directly whether its association with clonal behavior varied across environmental conditions.

Last, to test whether GEP3 usage tracks with environmental adaptability at scale, we exposed aliquots of the same ARID1A-knockdown population (262 clones) to the panel of microenvironmental stressors used earlier (Fig. 3A). For each clone, we related GEP3 usage to its stress-specific enrichment, defined as the log_2_ ratio of abundance under stress relative to unperturbed conditions. Among the 54 clones with sufficient representation in both datasets, higher GEP3 usage was associated with greater enrichment under CoCl_2_, serum starvation, and glutamine deprivation, but not under glucose deprivation or H_2_O_2_ (Fig. 5, K and L). The associations under CoCl_2_ and glutamine deprivation matched those observed in the defined 12-clone assay (Fig. 3A), reproducing the finding across independent experimental scales. In conclusion, large-scale clonal tracing shows that ARID1A loss robustly induces GEP3 usage across hundreds of clones and that higher GEP3 usage marks clones with greater transcriptional diversity and enhanced, context-dependent adaptability under environmental stress (Fig. 5M).

## DISCUSSION

A central challenge in cancer biology is to understand how transcriptional heterogeneity arises and is organized within tumors (*3*, *5*, *11*–*16*). Recent surveys have cataloged recurrent expression programs across tumors and shown that the states available to cancer cells are strongly context-dependent (*5*). Here, by coupling barcoded perturbation with clone-resolved multiomics, we found that loss of a single EM, ARID1A, increases transcriptional variability both among the descendants of a single founder cell and between independently derived clones. Programs with defined signatures, such as EMT-like and interferon-like programs, were also represented among ARID1A-deficient clones; however, in the independent BxPC3 replicate, they were not specific to knockdown and also appeared in controls—consistent with ARID1A loss relaxing constraints on pre-existing states rather than creating new ones. The feature that recurred most consistently in association with ARID1A loss was GEP3—a cNMF-derived component that corresponded to no dominant pathway signature and whose high-usage cells were less similar to one another than those of the coherent programs. These observations suggest that GEP3 may be better understood not as a discrete cell state but as a recurrent axis of transcriptional variability associated with ARID1A loss.

Such an interpretation would have implications for how components of this kind are interpreted. Factorization methods such as cNMF are generally used to identify coordinated modules, and a factor reflecting variability rather than coordination would lack both a dominant signature and a coherent set of coexpressing cells—features that might invite its dismissal as a residual or technical artifact. Several observations argue against that reading here: the principal properties of GEP3 were recovered in an independent BxPC3 experiment; related signature-poor components were observed in some additional cell lines; the observed combination of gene weights and clone-level association was not reproduced by the expression-shuffling procedure used here; and GEP3 usage was associated with both chromatin-accessibility variability and clone-level phenotypes. We note, however, that because GEP3 lacks a defining signature, its correspondence across cell lines rests on shared statistical properties rather than on gene identity—a weaker form of correspondence than for signature-defined programs, and one we present as such. Whether variability-associated factors of this kind arise more generally after perturbations that relax transcriptional constraints will require dedicated investigation.

The chromatin data suggest how this variability may be related to chromatin remodeling and what sustains the associated features. ARID1A loss did not establish new clone-specific chromatin identities but relaxed existing constraints, broadening accessibility at otherwise silenced, lineage-inappropriate loci while remaining largely within annotated regulatory elements. This partial derepression was accompanied by elevated peak entropy, which, measured in an independent modality, tracked with GEP3 usage rather than with transcriptional heterogeneity in general. Notably, this variability was not simply a passive consequence of losing a chromatin remodeler: degrading SMARCA4 reduced both GEP3 usage and the chromatin-accessibility entropy associated with it. This suggests that residual SWI/SNF activity does not merely fail to restrain the genome but actively participates in maintaining the derepressed landscape that ARID1A loss creates—a role consistent with reports that ARID1A-deficient cells assemble alternative cBAF complexes incorporating ARID1B, which compensate incompletely (*42*, *43*), and with the buffering of chromatin perturbations by functionally coupled remodelers.

What such variability might offer a tumor is suggested by the pattern of selection we observed, although our data do not establish the underlying mechanism directly. Clones with high baseline GEP3 usage showed no growth advantage under standard conditions, and ARID1A-deficient clones proliferated more slowly than controls. Preferential recovery was observed only under selection and only under some pressures: GEP3-enriched clones were favored under hypoxia-mimetic and glutamine-deprived conditions and during lung colonization, whereas glucose deprivation instead favored interferon-like and EMT-like clones. This context dependence is compatible with a bet-hedging–like model, in which maintaining a range of transcriptional profiles carries a cost under stable conditions but can pay off when conditions change. Such a model is intrinsically defined at the level of populations rather than of single cells—the relevant property is the range of profiles a lineage can access—which matches the clonal resolution of our lineage-tracing system. Consistent with this view, lung-engrafted populations derived from GEP3-enriched clones were no longer organized primarily by a GEP3-like component but by a lung-associated adaptive program, as were the few control clones that engrafted; nevertheless, GEP3 module scores were retained. Cells of different clonal origins were thus organized by a shared adaptive state under selection, consistent with the possibility that high baseline GEP3 usage marks clonal populations from which environment-specific states can emerge, rather than cells committed to a particular state.

This raises a question about how this variability has previously been described. EMT has long been linked to phenotypic plasticity, and the EMT-like GEP1 was indeed transcriptionally variable in our data (Fig. 1E)—yet GEP3, which lacks a defining signature, was more variable still. Intermediate or partial EMT states are themselves defined by mixed, low-specificity signatures, which makes them difficult to distinguish from a signature-poor, high-variability component such as GEP3 (GEP1, but not GEP3, showed a weak trend toward enrichment for curated partial-EMT signatures; fig. S4K). It is therefore conceivable that some of the plasticity historically attributed to intermediate EMT reflects an unrecognized contribution from GEP3-like variability that was grouped with EMT in the absence of distinguishing markers. This possibility is speculative, but it suggests that marker-independent, clone-resolved approaches may be needed to separate the two.

Our study leaves several questions open. Selection was measured at the level of GEP3-enriched clones, and we could not determine which cells within such a clone were the ones actually selected under a given pressure; addressing this would require lineage tracing at finer resolution than our current system provides. The factors that bias a given ARID1A-deficient clone toward a GEP3-high regime rather than toward an EMT- or interferon-dominant program also remain unknown. Last, one cell line, SUIT-2, did not show increased heterogeneity after ARID1A knockdown. SUIT-2 carries a truncating ARID1A mutation and may have adapted chronically to reduced ARID1A function, unlike the wild-type lines examined after acute knockdown; because chronic adaptation was not tested directly, this interpretation remains speculative, and we cannot exclude that the response to ARID1A loss depends on molecular context in ways not captured here.

Together, our findings suggest that EM loss may contribute to tumor evolution less by installing a new phenotype than by widening the range of phenotypes a lineage can reach. ARID1A loss did not push cells into a defined malignant state; rather, it appeared to lift constraints, with residual remodeling activity helping to hold the resulting landscape open. Clone-resolved perturbation makes this process visible and suggests that an important part of what epigenetic disruption produces may be not a state to be cataloged, but variability itself.

## MATERIALS AND METHODS

### Cell culture and reagents

BxPC3, HPAC, and SUIT-2 cells were cultured in RPMI 1640 medium (Nacalai Tesque) supplemented with 10% fetal bovine serum (FBS) and penicillin/streptomycin (Sigma-Aldrich) in a 5% CO_2_ incubator at 37°C. SW1990 cells were cultured in Dulbecco’s modified Eagle’s medium supplemented with 10% FBS and penicillin/streptomycin. HPAFII cells were cultured in minimum essential medium (MEM) with Earle’s Salts medium (Nacalai Tesque) supplemented with 10% FBS, sodium pyruvate, and penicillin/streptomycin (Sigma-Aldrich) in a 5% CO_2_ incubator at 37°C. All cell lines tested negative for *Mycoplasma*. Some BxPC3 clones were incubated with 1 μM AU-15330 (Selleck) or dimethyl sulfoxide (DMSO) (Sigma-Aldrich) for 24 hours (Western blot and scRNA-seq) or 4 hours (scATAC-seq).

### Cell proliferation

To measure cell proliferation, BxPC3 clonal populations were seeded in 96-well plates at a density of 3125 cells/cm^2^. The cells were cultured for 7 days, during which phase-contrast images were acquired every 24 hours using a 4× objective on an IncuCyte SX5 Live-Cell Analysis System (Sartorius). Furthermore, confluence was automatically quantified at each time point using the integrated IncuCyte analysis software.

### Morphological classification of the clonal populations

To assess the morphological heterogeneity among the clonal populations, bright-field images were captured for each clone using a BZ-X700 microscope (Keyence). The clones were classified into six predefined morphological types (Types 1–6) based on a stepwise decision-making process.

First, if the average cell diameter was ≥20 μm, then the clone was assigned to type 6. If this criterion was not met, then colony-level features were assessed. Clones, in which cells formed tightly packed and condensed colonies, were categorized as type 2. Clones with looser intercellular contacts and scattered arrangements, which were suggestive of a mesenchymal-like morphology, were classified as type 5. If the cells appeared flattened, with an enlarged surface area, prominent perinuclear halos, or surface wrinkling, then the clone was assigned to type 4. Clones containing cells with an average diameter exceeding 11 μm—larger than that of the parental line—were designated as type 3. Clones that did not meet these criteria and resembled the morphology of the parental cell line, characterized by cohesive growth and an epithelial-like appearance, were categorized as type 1.

### Reverse transcription quantitative polymerase chain reaction

Total RNA was extracted using the RNeasy Plus Mini Kit (QIAGEN) according to the manufacturer’s instructions. Reverse transcription was done using the PrimeScript RT Master Mix (TaKaRa). Quantitative PCR was carried out on a StepOnePlus Real-Time PCR System (Thermo Fisher Scientific) using SYBR Premix Ex Taq II (Tli RNaseH Plus; TaKaRa, #RR820A), as previously described (*44*–*46*). The following primer sequences used were as follows: KDM6A (forward: 5′-GCGGACAAAAGAAGAACCAG-3′; reverse: 5′-AGATGAGGCGGATGGTAATG-3′), SETD2 (forward: 5′-AAACACAACAACTGAACGAGG-3′; reverse: 5′- GAGTTTGCTT-GTCTGGGTCTC -3′), ARID1A (forward: 5′-CGGCCAATGGATGGCACATA-3′; reverse: 5′-TCGGCCAAACTGGAATGGAA-3′), SMARCA4 (forward: 5′-CTCGGTCCGTCAAAGTGAAGA-3′; reverse: 5′-AGCGGTCCTCCTCTTGTTCC-3′), and ACTB (housekeeping gene) (forward: 5′-AGAGCTACGAGCTGCCTGAC-3′; reverse: 5′-AGCACTGTGTTGGCGTACAG-3′). Relative gene expression was calculated using the ΔΔCt method and normalized to ACTB expression.

### Western blot analysis

Western blot analysis was performed as previously described (*44*, *45*, *47*) with minor modifications. Cell pellets were lysed in buffer containing 0.1 M tris-HCl (pH 7.5), 10% glycerol, and 1% SDS; boiled for 5 min; and centrifuged at 15,000 rpm for 10 min. Protein concentrations were determined using the BCA Protein Assay Kit (Pierce). Equal amounts of protein were separated by SDS–polyacrylamide gel electrophoresis (SDS-PAGE) and transferred onto polyvinylidene difluoride membranes (Millipore). The membranes were blocked with 5% skim milk (Megumilk) or 5% bovine serum albumin (Sigma-Aldrich) in tris-buffered saline containing 0.1% Tween 20 (TBST). They were incubated overnight at 4°C with the following primary antibodies diluted in blocking buffer: anti-ARID1A (1:1000; Cell Signaling Technology, #12354), anti-BRG1 (1:1000; Cell Signaling Technology, #49360), and anti–α-tubulin (1:5000; Sigma-Aldrich, clone B-5-1-2, T5168). After washing three times in TBST, the membranes were incubated with horseradish peroxidase–conjugated secondary antibodies, which included either anti-mouse immunoglobulin G (IgG; 1:5000; Cell Signaling Technology, #7076) or anti-rabbit IgG (1:5000; Cell Signaling Technology, #7074) for 1 hour. Moreover, signals were visualized using the SuperSignal West Femto Maximum Sensitivity Substrate (Thermo Fisher Scientific) and captured with an Odyssey Fc Imaging System (LI-COR).

### Plasmid construct

The shPseuMO-Tag vector contains a DNA barcode in the 3′ untranslated region of a polyadenylate [poly(A)]–tagged mCherry-PuroR fusion construct, flanked by Tn5 adaptor sequences. This design enables simultaneous capture of the same barcode during 3′ Chromium scRNA-seq (10x Genomics) and ddSEQ scATAC-seq (Bio-Rad) without requiring custom reagents or protocols.

The shPseuMO-Tag plasmid was constructed based on the pLenti cytomegalovirus (CMV) green fluorescent protein (GFP) Puro (658-5) vector (Addgene, #17448). The cPPT/CTS, CMV enhancer, CMV promoter, and enhanced GFP regions were removed using HpaI and SalI restriction enzymes. Next, the U6 promoter, shRNA sequence (flanked by BamHI and SalI restriction sites), cPPT/CTS, CMV enhancer, CMV promoter, and a mCherry-P2A-PuroR-Read N1-PCR handle1-Stuffer (for inserting cell barcode sequences)-PCR handle2-Read N2-SV40 poly(A) signal cassette were inserted via In-Fusion 5X HD Cloning Plus (Takara Bio, #638909), using 20– to 30–base pair (bp) homologous sequences, which resulted in shPseuMO-Tag-Stuffer (available upon request). The full-length sequence is provided in table S7.

The sequences used for shRNA were as follows: KDM6A (5′-GATGCAAGTCTATGACCAATT-3′; target sequence of predesigned shRNA) (TRCN0000107763, Sigma-Aldrich), SETD2 (5′-AAACCAACAGTCTGTCAGTGTA-3′) (*48*), ARID1A [5′-GCCTGATCTATCTGGTTCAAT-3′ (ARI3) and 5′-GCATCCTTCCATGAACCAATC-3′ (ARI4) (*49*)], and SMARCA4 (5′-CGGCAGACACTGTGATCATTT-3′) (*50*). Nontargeting shRNA (scramble) sequence was used as a negative control (5′-AGTCTTAATCGCGTATAAG-3′) (*49*). The hairpin sequence used was as follows: 5′-CTGTGAAGCCACAGATGGG-3′ (*51*).

To assemble the cell barcode, based on a slight modification of a previous report (*52*), a 59-bp oligonucleotide containing a 10-bp random sequence flanked at both ends by a 5-bp HashTag sequence and a reverse extension primer was obtained from Integrated DNA Technologies: 5′-GAGCCTCGTCTCCTGACTNNNNNNNNNNNNNNNNNNNNCGTTGTGAGACGCATGCTGCA-3′. The 5-bp Hash-Tag sequences were as follows: shSCR: ATGCA; shKDM: AGCTG; shSET: TGCTA; shARID: CTGAA; shSMA: GCCAA; shSCR2: ATGCA; shARID3: CGTGC; and shARID4: CTGAA. The following extension reaction was performed to generate double-stranded barcode-shRNA oligonucleotides: 25 μl of NEBNext High-Fidelity 2X PCR Master Mix (NEB, M0541), 1 μl of 100 μM cell barcode, 2 μl of 100 μM reverse extension primer (5′-TGCAGCATGCGTCTCACAACG-3′), and 22 μl of water. The reaction conditions were as follows: 98°C for 2 min, 10× (65°C for 30 s, 72°C for 10 s), 72°C for 2 min, and hold at 4°C. The double-stranded barcode-shRNA oligonucleotide was purified using a QIAquick PCR Purification kit (QIAGEN, 28104). The double-stranded product contained two Bsm BI sites that, upon digestion, generated complementary overhangs for ligation into shPseuMO-Tag-Stuffer. Next, 1.5 μg of BsmBI-v2 digested (New England Biolabs, R0739) shPseuMO-Tag-Stuffer vector was added to a Golden Gate Assembly reaction with the double-stranded barcode insert at a molar ratio of 1:5 and cycled 100× (42°C for 2 min and 16°C for 5 min). The reaction was purified and concentrated in 10 μl of water using the DNA Clean & Concentrator Kit (Zymo, D4033) and transformed into NEB Stable Competent *Escherichia coli* (New England Biolabs, C3040). Transformants were inoculated into 500 ml of LB medium containing ampicillin (100 μg ml^−1^) and incubated overnight at 30°C. Bacterial cells were collected by centrifugation at 6000 RCF at 4°C for 15 min, and plasmid DNA was extracted using a QIAGEN Plasmid Plus Maxi kit (QIAGEN, 12943).

The PseuMO-Tag vector was constructed by removing the shRNA cassette from the shPseuMO-Tag via HpaI and SalI digestion, followed by a blunting reaction. The PuroR sequence was replaced with a BlastR sequence cloned from lentiCRISPR v2-Blast (Addgene, #83480) using In-Fusion cloning. For barcode construction, a 77-bp oligonucleotide containing an 18-bp random sequence (*53*) was synthesized: 5′-GAGCCTCGTCTCCTGACTATGCANNNGTNNNCTNNNAGNN NTGNNNCANN NTGCATCGTTGTGAGACGCATGCTGCA-3′, along with the same reverse primer as above. Barcode extension, cloning, and plasmid preparation were performed as described. The full-length sequence is provided in table S7.

### Lentivirus production

Human embryonic kidney (HEK) 293T cells were seeded at 3 × 10^6^ cells per 10-cm dish. The following day, 2.5 μg of the lentiviral vector was mixed with 1.25 μg of pCMV-VSVG-RSV/Rev envelope vector and 1.25 μg of pMDLg/pRRE packaging vector in 250 μl of Opti-MEM Reduced Serum Medium (Gibco, 31985-070). FuGENE HD Transfection Reagent (16 μl; Promega, E2311) was added, and the mixture was vortexed, left to incubate for 15 min, and carefully transferred to the HEK293T culture. The medium was refreshed 16 to 24 hours posttransfection, and virus supernatant was collected 48 and 72 hours later, passed through a 0.45-μm filter (Millipore Millex-HV, SLHVR33RS), aliquoted, snap-frozen in liquid nitrogen, and stored at −80°C until further use.

### Clonal lineage tracing and parallel multiomics profiling

To evaluate intraclonal heterogeneity, BxPC3 cells were seeded at a density of 5000 cells per well in 24-well plates (Figs. 1 to 4). The following day, the cells were transduced with shPseuMO-Tag lentiviral vectors encoding shRNAs targeting ARID1A (ARI3 and ARI4) or a nontargeting control (SCR2) at a multiplicity of infection of 6 to 8 in the presence of polybrene (10 μg/ml). The following day, the transduced cells were subjected to limiting dilution in 96-well plates to establish single cell–derived clones and cultured with puromycin (2 μg/ml; 3 days) to select cells infected with shPseuMO-Tag. Each clone was expanded until it reached ∼1 million cells. The expanded clonal populations were cryopreserved in Bambanker (Nippon Genetics) and stored for subsequent assays. A total of 28 clones (8 SCR2 clones, 9 ARI3 clones, and 11 ARI4 clones) were used in subsequent experiments. Experimental parameters were as follows: number of clones tracked: 28, culturing strategy: isolated single-cell cloning, and expansion time: ∼20 cell divisions.

In this single-cell cloning design, a clone was operationally defined as the progeny of a single founder cell isolated by limiting dilution after shPseuMO-Tag transduction. All cells within a clone therefore shared a common post-perturbation ancestor at the time of isolation. The rebarcoding of an established clone provided a finer barcode-defined sublineage resolution among its descendants.

Group 3 clones used in downstream functional experiments were selected before those experiments on the basis of high clone-level median GEP3 usage and/or high median peak entropy, together with confirmed ARID1A knockdown. Clones with high GEP3 usage included ARI4#21, ARI4#20, and ARI4#23, whereas clones with high peak entropy included ARI4#23, ARI4#07, ARI4#14, and ARI4#21. ARI4#21, one of the clones with the highest GEP3 usage, was selected for the rebarcoding experiment, with SCR2#04 as the group 2 control. For the AU-15330 experiments, SCR2#01 and SCR2#04 from group 2 and ARI4#14, ARI4#20, ARI4#21, and ARI4#23 from group 3 were analyzed. These experiments were designed to characterize selected high-GEP3 and/or high-entropy clones and were not intended to represent the full range of group 3 behavior.

To assess the reproducibility and cell line dependence of ARID1A-associated transcriptional diversification, the shPseuMO-Tag knockdown-and-barcoding workflow was repeated independently in BxPC3 cells and applied to HPAC, SW1990, SUIT-2, and HPAFII cells, as described above under the “Clonal lineage tracing and parallel multiomics profiling” section. Cells were transduced with shPseuMO-Tag lentiviral vectors encoding an ARID1A-targeting or nontargeting control shRNA and selected with puromycin where applicable. Single cell–derived clones were established, expanded, and subjected to downstream single-cell profiling for BxPC3, HPAC, SW1990, and SUIT-2 cells. Because single cell–derived HPAFII clones could not be established, HPAFII cells were analyzed as a pooled population after transduction and selection.

### Time-course scRNA-seq following ARID1A knockdown

To examine the temporal dynamics of transcriptional diversification after ARID1A depletion, BxPC3 cells were transduced with shPseuMO-Tag lentiviral vectors encoding either a nontargeting control shRNA or ARID1A-targeting shRNAs, as described above. Cells were harvested at day 5 and day 16 after transduction, corresponding ∼2 to 3 and 10 cell divisions, respectively. Viable cells were collected at each time point and subjected to scRNA-seq using the Chromium Single Cell 3′ protocol.

The resulting scRNA-seq data were processed using Cell Ranger and Seurat as described below. The transcriptional diversity was quantified using the principal component analysis (PCA)–based diversity index, and transcriptional homogeneity was quantified using NMI as described below. GEP3 module signal was calculated using Seurat’s AddModuleScore() function with the original GEP3 gene set derived from the 28-clone BxPC3 analysis.

### Clonal lineage tracing and parallel multiomics profiling

To assess heterogeneity among independently perturbed founder lineages, BxPC3 cells were seeded at a density of 5000 cells per well in 24-well plates (Fig. 5). The following day, the cells were transduced with shPseuMO-Tag lentiviral vectors encoding shRNAs targeting KDM6A, SETD2, ARID1A, SMARCA4, or a nontargeting control (Scramble) at a multiplicity of infection of 6 to 8 in the presence of polybrene (10 μg/ml). The next day, 400 transduced BxPC3 cells, representing ∼400 individual clones, were reseeded into 24-well plates and cultured with puromycin (2 μg/ml) for 3 days to select the successfully transduced cells. The cells were expanded until reaching ∼10 million cells. After 19 to 26 days postinfection, the expanded populations were cryopreserved in Bambanker (Nippon Genetics) and stored until subsequent analyses. This procedure was performed independently for each shRNA and resulted in a total of ∼2000 clones analyzed across the five conditions. Experimental parameters were as follows: number of clones tracked: ∼400 clones per shRNA (∼2000 clones in total), culturing strategy: pooled culture, and expansion time: ∼15 cell divisions.

In this pooled design, a “clone” denotes the progeny of one of ∼400 independently transduced founder cells per condition, each marked by a distinct PseuMO-Tag barcode; differences measured across these barcodes therefore reflect interclonal heterogeneity among independently perturbed founders rather than diversification within a single lineage.

### Analytical characterization of GEP coherence and transcriptional dispersion

cNMF usage values were normalized to sum to one within each cell. For each GEP, cells with nonzero usage were ranked according to their usage values, and cells in the top 5% were selected. Up to 100 cells were randomly sampled from this population in each of 200 iterations. Cell-cell similarity was calculated using the top 20 genes associated with each GEP as the median pairwise Pearson correlation between sampled cells. Gene-gene coexpression was calculated as the median pairwise Pearson correlation between the same top-ranked genes across sampled cells; genes with no variance among the sampled cells were excluded from the correlation calculation.

To evaluate gene-module coherence across different levels of GEP3 usage, GEP3-positive cells were divided into five percentile bins: 0 to 25, 25 to 50, 50 to 75, 75 to 95, and 95 to 100%. Equal numbers of cells, determined by the smallest bin and capped at 100 cells, were randomly sampled from each bin in each of 200 iterations. Gene-module coherence was calculated as the median pairwise Pearson correlation among the top 20 GEP3-associated genes.

To quantify clone-level transcriptional dispersion, 2000 variable genes were selected from the log-normalized expression matrix, followed by scaling and principal component analysis. Clones represented by at least 20 cells were retained. For each clone, a centroid was calculated in the space of the first 30 principal components, and transcriptional dispersion was defined as the median Euclidean distance of individual cells from the corresponding clone centroid. Clone-level GEP3 usage was summarized as the median usage across cells.

For the expression-shuffling analysis, expression values for each gene were independently permuted across cells, preserving each gene’s expression distribution while disrupting gene-gene coexpression, cell-level expression structure, and associations with clonal identity. Each shuffled matrix comprised 1500 highly variable genes together with the top 50 GEP3-associated genes. Seven-factor NMF was independently fitted to each of 30 shuffled matrices, generating 210 null factors. Factor usage and cell-cell similarity were calculated using the same procedures applied to the observed GEPs. For each null factor, median usage was calculated per clone and correlated with the observed clone-level transcriptional dispersion. Empirical *P* values were calculated from all finite null-factor values using a +1 correction. For cell-cell similarity, a two-sided empirical *P* value was calculated according to the absolute deviation from the median of the null distribution. For the association with clone-level transcriptional dispersion, a one-sided empirical *P* value was calculated as the proportion of null factors with a Spearman’s ρ equal to or greater than the observed value.

### In vivo 10-clone lung-colonization assay

For the in vivo lung-colonization assay, 10 individual clonal populations (SCR2#01, SCR2#04, SCR2#05, SCR2#06, SCR2#13, ARI4#07, ARI4#14, ARI4#20, ARI4#21, and ARI4#25) were harvested and resuspended in Hank’s balanced salt solution (Thermo Fisher Scientific). Equal numbers of cells from each clone were mixed, and a total of 20,000 cells in 200 μl were injected intravenously into the lateral tail vein of 7- to 8-week-old female NOD scid gamma (NSG) mice (the Jackson Laboratory Japan). Lung tissues were collected at 14 or 28 days postinjection. Genomic DNA (gDNA) was extracted using the DNeasy Blood & Tissue Kit (QIAGEN, 69504).

For rebarcoding experiments, two clones (SCR2#04 and ARI4#21) were independently rebarcoded, harvested, and resuspended in Hank’s balanced salt solution. A total of 0.2 million cells in 200 μl were intravenously injected into the tail veins of 7-to 8-week-old female NSG mice. The remaining cells (∼1 million) were plated into 10-cm dishes in triplicate and cultured under standard conditions. Upon reaching confluence, the cells were passaged at a 1:5 ratio for five consecutive passages. The genomic DNA was extracted from the confluent cells after the fifth passage using the DNeasy Blood & Tissue Kit.

All animal procedures were performed in accordance with protocols approved by the Animal Care and Use Committee of the Japanese Foundation for Cancer Research (approval number 22-01-2).

For barcode library preparation, 500 ng of extracted gDNA was used as input for first-round PCR using the NEBNext High-Fidelity 2× PCR Master Mix (NEB, M0541). The thermal cycling conditions were as follows: 98°C for 30 s; followed by 19 to 25 cycles of 98°C for 10 s, 67°C for 30 s, and 72°C for 30 s. The PCR products were purified using 0.6× SPRIselect beads (Beckman Coulter) and eluted in 10 μl of TE buffer. The first-round PCR products were diluted 1:5 and used as templates for second-round PCR (50-μl reactions) to incorporate sample indices. The thermal cycling conditions were as follows: 98°C for 30 s, followed by 5 cycles of 98°C for 10 s and 72°C for 30 s. Dual-sided size selection was performed using SPRIselect beads with exclusion and selection ratios of 0.8× and 1.2×, respectively. The final products were eluted in 10 μl of TE buffer. The library size distribution and concentration were assessed using a TapeStation HSD1000 System (Agilent Technologies) and a Quantus Fluorometer (Promega), respectively. The libraries were sequenced on an Illumina NextSeq 550 platform using paired-end reads (read 1: 75 bp; index 1: 8 bp; index 2: 8 bp; read 2: 75 bp). The primer sequences used for library preparation are provided in table S6.

### In vivo 12-clone lung-colonization assay and ex vivo scRNA-seq

For the defined 12-clone lung-colonization assay, 12 shPseuMO-Tag-labeled BxPC3 clones spanning all four transcriptional groups were mixed at equal proportions. The pool consisted of three group 1 clones (ARI3#16, ARI3#27, and ARI4#08), three group 2 clones (SCR2#01, SCR2#04, and SCR2#07), three group 3 clones (ARI4#07, ARI4#20, and ARI4#21), and three group 4 clones (ARI3#18, ARI3#20, and ARI3#09). Cells were trypsinized, mixed in equal numbers from each clone, centrifuged at 1000 rpm for 3 min at 4°C, and resuspended in Hank’s balanced salt solution. A total of 200,000 cells per mouse, corresponding to 16,667 cells per clone, were injected intravenously into the lateral tail vein of five 7- to 8-week-old female NSG mice. In parallel, aliquots of each clone were cryopreserved in BAMBANKER as input stocks.

Two weeks after injection, mice were euthanized and lungs were harvested. Lung tissues were minced on ice and dissociated into single-cell suspensions using the Tumor Dissociation Kit, human (Miltenyi Biotec) with the gentleMACS program 37C_h_TDK_1. After dissociation, red blood cells were lysed, and the resulting cell suspensions were counted.

For FACS isolation of lung-derived tumor cells, dissociated cells were labeled with cell multiplexing oligos where applicable and stained with SYTOX Green to exclude dead cells. Viable tumor cells were sorted as mCherry-positive and SYTOX Green-negative cells. Sorted cells from the five injected mice were counted and subjected to scRNA-seq library preparation. For the matched in vitro control, the cryopreserved preinjection 12-clone BxPC3 pool was thawed and processed in parallel. Cells were sorted using the same mCherry-positive and SYTOX Green-negative gating strategy, counted, and subjected to scRNA-seq library preparation in a separate lane. This matched in vitro sample was used as the baseline reference for comparison with lung-derived cells.

### In vitro environmental stress competition assay

The following conditions were tested: unperturbed complete medium, hypoxia-mimetic stress induced by 200 μM cobalt(II) chloride hexahydrate (CoCl_2_; Sigma-Aldrich, C8661), low-serum medium containing 0.1% FBS, glucose-depleted medium using RPMI 1640 without glucose with l-glutamine (Nacalai Tesque, 09892-15), glutamine-depleted medium using RPMI 1640 without l-glutamine (Nacalai Tesque, 05176-25), and oxidative stress induced by 0.1 mM H_2_O_2_ (FUJIFILM Wako). CoCl_2_, low-serum, and glutamine-depleted conditions were maintained for 1 week. H_2_O_2_ and glucose-depleted conditions were applied transiently for 3 days, after which cells were returned to standard complete medium. After each treatment, cells were expanded in standard medium until they reached ∼70 to 80% confluence. At the end of the assay, cells were harvested, and genomic DNA was extracted using the DNeasy Blood & Tissue Kit.

For the 12-clone environmental stress competition assay (Fig. 3, A to C), the same 12 BxPC3 clones used for the in vivo lung-colonization assay were pooled in equal numbers. For the large-scale pooled stress assay (Fig. 5, K and L), the ARID1A-knockdown population generated in the pooled shPseuMO-Tag experiment (Fig. 5A) was used. Cells were seeded in 6-cm dishes at a density of 3125 cells/cm^2^. Triplicate dishes were prepared for each of the six culture conditions, and two baseline control dishes were harvested at the start of the experiment.

### scRNA-seq library preparation and sequencing

For the molecular profiling of clones analyzed using the Figs. 1 to 4, we performed single-plex, scRNA-seq to minimize batch effects. Barcoded cell populations transduced with shPseuMO-Tag vectors targeting KDM6A, SETD2, ARID1A, SMARCA4, or a nontargeting control (shSCR) were processed individually (Fig. 5), along with 28 distinct clones derived from shSCR2-, shARI3-, or shARI4-transduced cells (Figs. 1 to 4).

Cryopreserved cell stocks were rapidly thawed at 37°C, and cell viability was confirmed to exceed 90% before further processing. An equal number of viable cells was pooled and loaded onto a 10x Genomics Chromium controller for single-cell partitioning and barcoding. cDNA synthesis and library preparation were performed using the Chromium Single Cell 3′ v3 protocol (10x Genomics, Pleasanton, CA) according to the manufacturer’s instructions. Libraries were sequenced on an Illumina NextSeq 550 platform (Illumina, CA, USA) with paired-end reads (read 1: 28 bp; index 1: 10 bp; index 2: 10 bp; read 2: 90 bp). To analyze SMARCA4 inhibition by AU-15330 (Fig. 4), cells from distinct clones treated with DMSO or AU-15330 were pooled within each treatment group. Specifically, clones exposed to the same conditions were mixed in equal proportions before sample multiplexing using the 10x Genomics CellPlex 3′ Multiplexing Kit Set A. Equal numbers of pooled cells were loaded into the Chromium controller and processed as described above. The demultiplexing of individual clones was performed using a custom PseuMO-Tag Decoder pipeline (see below), whereas CellPlex sample identities were resolved using Cell Ranger v6.1.2.

To selectively enrich reads containing the PseuMO-Tag sequence, a three-round nested PCR strategy was used. For the first-round PCR, ∼20 ng of scRNA-seq library was amplified in a 100-μl reaction using NEBNext High-Fidelity 2× PCR Master Mix (NEB, M0541) and then split into two 50-μl reactions. The thermal cycling conditions were as follows: 98°C for 30 s; 15 to 20 cycles of 98°C for 10 s, 67°C for 30 s, and 72°C for 20 s. The pooled reactions were purified with SPRIselect beads (Beckman Coulter) using a dual size-selection (exclusion ratio: 0.56×; selection ratio: 0.85×) and eluted in 10 μl of TE buffer. For the second-round PCR, 10 ng of the purified product was used in a 50-μl reaction under the following conditions: 98°C for 30 s; 5 cycles of 98°C for 10 s, 65°C for 30 s, and 72°C for 20 s. The products were purified using 0.9× SPRIselect beads and eluted in 10 μl of TE buffer. For the third-round PCR, 10 ng of the second-round product was amplified using the same PCR conditions with three cycles at 67°C annealing. The final products were purified with 0.9× SPRIselect beads and eluted in low-EDTA TE buffer. The final barcode-enriched libraries were quality-checked using an Agilent TapeStation (HSD1000 kit) and quantified with a Quantus Fluorometer (Promega). Sequencing was done using an Illumina NextSeq 550 platform (paired-end, read 1: 75 bp; index 1: 8 bp; index 2: 8 bp; read 2: 75 bp). The primer sequences are listed in table S6.

### scATAC-seq library preparation and sequencing

For single-cell chromatin accessibility profiling, we performed single-plex, single-cell ATAC sequencing to minimize batch effects. Barcoded cell populations transduced with the shPseuMO-Tag vectors targeting KDM6A, SETD2, ARID1A, SMARCA4, or a nontargeting control (shSCR) were processed individually (Fig. 5). Similarly, the 28 clones derived from shSCR2-, shARI3-, or shARI4-transduced cells were processed (Figs. 1 to 4). The pooled clone mixtures were treated with either DMSO or AU-15330 (Fig. 4).

Cryopreserved cells were thawed at 37°C, and viability was confirmed to exceed 90%. Equivalent numbers of viable cells were used for each condition. Single-cell ATAC-seq libraries were generated using the SureCell ATAC-Seq Library Prep Kit and SureCell ddSEQ Index Kit (Bio-Rad) according to the manufacturer’s instructions. Libraries were sequenced on an Illumina NextSeq 550 platform using the following read structure: Read 1, 118 cycles (custom primer); i7 index, 8 cycles; read 2, 40 cycles. Raw FASTQ files were processed using the ATAC-Seq Analysis Toolkit (Bio-Rad) for debarcoding and alignment. The demultiplexing of individual clones was performed using a custom PseuMO-Tag Decoder pipeline.

To selectively enrich the reads containing the PseuMO-Tag barcode, two-round nested PCR was performed. For first-round PCR, ∼50 ng of scATAC-seq library DNA was used in a 100-μl reaction that was split into two 50-μl aliquots. The thermal cycling conditions were as follows: 98°C for 30 s; 15 to 20 cycles of 98°C for 10 s, 67°C for 15 s, and 72°C for 15 s. The pooled products were purified with SPRIselect beads (dual size selection, exclusion: 0.95×, selection: 1.2×) and eluted in 10 μl of TE buffer. The second-round PCR used 10 ng of the purified product in a 50-μl reaction, cycled at 98°C for 30 s, followed by 5 cycles of 98°C for 10 s, 66°C for 15 s, and 72°C for 15 s. The products were purified again using the same dual size selection strategy. The final barcode-enriched libraries were assessed using an Agilent TapeStation (HSD1000 kit) and quantified using a Quantus Fluorometer (Promega). Sequencing was performed on an Illumina NextSeq 550 platform with the same read structure as the original scATAC-seq libraries. The primer sequences used for enrichment are listed in table S6.

### CUT&Tag library preparation and sequencing

CUT&Tag was used to profile SMARCA4 binding in parental BxPC3 cells and in the clonal populations, SCR2#01, SCR2#04, ARI4#14, and ARI4#21. The experiments were performed using the CUT&Tag-IT Assay Kit (Active Motif, #53160) with modifications based on the manufacturer’s protocol and a previous report (*54*). Approximately 200,000 cells were fixed in 0.1% paraformaldehyde for 10 min at room temperature and quenched with 125 mM glycine. After washing, the cells were gently scraped, incubated with concanavalin A–coated magnetic beads and supplemented with 40 μL of CUT&Tag-IT Spike-in Nuclei (Active Motif, #53168) for normalization. The bead-bound nuclei were resuspended in 50 μl of antibody buffer containing 1 μl of anti-SMARCA4 antibody (Brg1 [D1Q7F] Rabbit mAb, Cell Signaling Technology, #49360) and 1 μl of spike-in antibody (Active Motif, #53168). The samples were incubated overnight at 4°C on a nutator. After washing to remove unbound antibodies, guinea pig anti-rabbit secondary antibody (Active Motif, included in the kit) was added and incubated for 30 min at room temperature. After additional washes, the nuclei were incubated with CUT&Tag-IT-assembled pA-Tn5 Transposomes for 1 hour at room temperature. Tagmentation was done by incubating the samples in Tagmentation Buffer for 2 min at 4°C, followed by 20 min at 37°C. To release tagmented DNA, the samples were treated with SDS and Proteinase K at 55°C for 1 hour. DNA was purified using the DNA Purification Columns provided in the kit. Library amplification was achieved using indexed primers according to the manufacturer’s instructions. Libraries were size-selected using SPRIselect beads (Beckman Coulter), assessed for fragment size distribution with an Agilent TapeStation (HSD1000), and quantified using a Quantus Fluorometer (Promega). The final libraries were sequenced on an Illumina NextSeq 550 platform with paired-end reads (read 1: 38 bp; index 1: 8 bp; index 2: 8 bp; read 2: 38 bp).

### Transcriptomic heterogeneity estimation in the clinical samples

To determine the relationship between the expression of EMs (*35*) and transcriptomic heterogeneity in primary human pancreatic tumors, we used two complementary computational approaches with bulk RNA-seq data from TCGA pancreatic adenocarcinoma cohort.

First, we adopted Shannon’s equitability-based method, with minor modifications from a previously published protocol by Hinohara *et al.* (*32*). For each EM, the patients were stratified into four quartile groups of equal size based on gene expression levels. Shannon’s equitability was calculated for each tumor based on the distribution of expression values across all detected genes. This metric normalizes Shannon’s entropy to a [0,1] scale, in which 1 indicates uniform gene expression and values near 0 indicate transcriptional dominance by a small subset of genes.

Second, we used a PCA-based transcriptomic diversity scoring method adapted from Ma *et al.* (*36*). PCA was performed using the prcomp function in R for all patient samples. Top *n* principal components (PCs) were extracted to represent the dominant axes of transcriptomic variation. Each patient was projected into this PC space, and the centroid of the population was computed for each quartile group. Transcriptomic diversity was defined as the mean Euclidean distance of each patient to the global centroid and calculated as follows

$$\text{Diversity score}=\frac{1}{m}\sum_{i=1}^{m}\sqrt{\sum_{j=1}^{n}{\left(x_{\mathrm{ij}}-\mu_{j}\right)}^{2}}$$

Here, *m* is the number of patients, *n* is the number of PCs, *x_ij_* is the *j*th PC score of the *i*th patient, and μ_j_ is the mean of the *j*th PC for all patients. To minimize the influence of outliers, samples with PC values exceeding ±3 SDs for any of the top three PCs were excluded. The top 30 PCs were selected on the basis of the cumulative variance explained and the stability of the resulting diversity scores.

### Analysis of the clonal distribution

To extract high-quality barcode sequences from the raw amplicon sequencing reads, the 5′ adaptor sequences (5′-GTCGAGGCAGGAAACAGCTATGACTATGCA-3′) and 3′ adaptor sequences (5′-TGCATCGTTGAGCAATAA-3′) were trimmed using cutadapt (v4.4). Reads containing ambiguous nucleotides (Ns) shorter than 28 nucleotides or lacking the characteristic barcode motif (5′-NNNGTNNNCTNNNAGNNNTGNNNCANNN-3′) were excluded. The remaining reads were filtered to retain only those with an average Phred quality score ≥ 30. The qualified barcode sequences were subjected to error correction using Starcode (v1.4) with a maximum Levenshtein distance of 1. The corrected barcode counts were aggregated for each sample and subsequently used for downstream clonal distribution analyses.

### Clone calling; PseuMO-Tag decoder

Demultiplexing of individual clones was done using a custom pipeline, *PseuMO-Tag Decoder*, which was developed to assign clonal identities based on synthetic barcodes introduced via the shPseuMO-Tag vector (fig. S2).

For scRNA-seq data, amplicon-seq FASTQ files were generated as described in the “scRNA-seq library preparation and sequencing” section. From read 1, the first 16 and 12 bp were extracted and designated as the cell barcode (CB) and unique molecular identifier (UMI), respectively. Error correction was performed and allowed up to one mismatch (Hamming distance ≤ 1). Adapter sequences (5′-CAGGAAACAGCTATGACT-3′) were trimmed from Read 2 using cutadapt, and a barcode region composed of HashTag-PseuTag-HashTag (5 bp–10 bp–5 bp) was extracted. The HashTag and PseuTag sequences were independently error-corrected, allowing one mismatch. PseuTag error correction was performed using Starcode (*55*). To remove PCR duplicates, the read counts were collapsed by UMI to generate CB-PseuTag pairs. Only cells with at least two UMI-supported PseuTag reads were retained for downstream analysis.

For scATAC-seq data, clonal assignment was done using the corresponding amplicon-seq FASTQ files (see the “scATAC-seq library preparation and sequencing” section). CBs were identified from read 1 using the ATAC-Seq Analysis Toolkit (Bio-Rad). Read 2 was processed similarly to the scRNA-seq workflow using the adapter sequence: 5′-CGCTCGCTAGTTATTGCTCAACG-3′, followed by HashTag and PseuTag extraction and error correction as described above.

To integrate clonal information across the scRNA-seq and scATAC-seq datasets, shRNA identities were assigned to cells, in which a single HashTag accounted for >60% of the total UMI counts. For clonal classification, a cell-by-PseuTag count matrix was constructed separately for each shRNA group.

Hierarchical clustering was performed using the heatmap3 R package (v1.1.9) with Ward.D linkage and cells within the same cluster were assigned to the same clone. In Fig. 4, cells were directly assigned to individual clones if a single dominant PseuTag accounted for more than 60% of the total PseuTag counts. This framework enabled the robust linkage of single-CBs to their corresponding clonal identities (PseuTags), which facilitated clone-aware single-cell transcriptomic and epigenomic analyses.

### Data analysis for clonal heterogeneity

For scRNA-seq analysis, raw sequencing reads were processed using the Cell Ranger pipeline (v6.1.2, 10x Genomics) and aligned to the GRCh38 human reference genome to generate gene-by-cell expression matrices as described below [see the “Data analysis for clonal heterogeneity” and (Figs. 1 to 4)]. The demultiplexing of individual clones was done using the custom PseuMO-Tag Decoder pipeline.

Downstream analyses were performed using the Seurat R package (v4.0.1) (*56*) with modified quality control thresholds. Cells with >10% mitochondrial transcript content, fewer than 800 detected genes, or more than 4000 detected genes were excluded.

Gene expression matrices were log-normalized and scaled, and PCA was performed as previously described. The top 30 PCs were used for clustering (FindNeighbors() and FindClusters() with a resolution of 0.4) and UMAP visualization.

GEPs were identified using cNMF (*40*). Factorization was performed with seven components (--components 7) and a local density threshold of 0.2. The top 50 genes for each GEP were subjected to enrichment analysis using the Enrichr web server (*57*–*59*) against the MSigDB Hallmark 2020 and GO Biological Process 2025 databases. GEPs were manually annotated on the basis of enrichment profiles. Clone-level GEP annotations were assigned by computing the median GEP activity per clone, and hierarchical clustering was performed using the heatmap3 package (method = “ward.D2”). Clones were grouped into four transcriptional classes based on the dendrogram structure.

The transcriptional regulatory inference of GEP3-associated programs was performed with decoupleR (v1.6.1) (*60*) and leveraged the CollecTRI database for prior knowledge. TF activity scores were inferred by the ULM method [run_ulm()].

For the scATAC-seq data, the raw reads were processed and analyzed using the ArchR package (v1.0.2) (*61*) as described above, with minor modifications. Demultiplexing was performed using the PseuMO-Tag Decoder pipeline. Clone-specific marker peaks were identified with getMarkerFeatures() followed by filtering with thresholds of false discovery rate (FDR) ≤ 0.5 and log_2_ fold change ≥ 0.25. TF activity was inferred using chromVAR deviation scores with motif annotations from the CIS-BP database. The 100 most variable motifs (*z*-scored) were visualized using the ComplexHeatmap package (v2.6.2).

To quantify chromatin accessibility at GEP3-associated regulatory regions, 100 cells per clone were randomly sampled, and the number of Tn5 fragments overlapping the promoter regions of the top five GEP3-associated genes was calculated using getFragmentsFromProject(). Sampling was repeated 100 times per clone, and the results were visualized using boxplots [median, interquartile range (IQR), and whiskers at 1.5× IQR].

### Cross-dataset cNMF and component comparison

cNMF was performed separately for each clone-resolved dataset using a dataset-specific factorization rank: BxPC3_2, *k* = 7; SW1990, *k* = 8; HPAC, *k* = 9; and SUIT-2, *k* = 8. Because the factorization was performed independently, similarly numbered components across datasets were not assumed to represent identical gene-expression programs. Pairwise similarity between components was evaluated using the Jaccard index calculated from the top 50 genes of each component. Pathway enrichment was evaluated against the MSigDB Hallmark 2020 and Cancer Cell Line Encyclopedia gene-set libraries using Enrichr. For each dataset, clone-level component usage was summarized as the median usage across cells and correlated with clone-level median peak entropy using Pearson’s correlation.

### Analysis of matched in vitro and ex vivo scRNA-seq data

Matched in vitro and lung-derived ex vivo scRNA-seq datasets from the 12-clone pool were processed using Cell Ranger with the human reference genome and analyzed using Seurat. Cells with low transcript counts, high mitochondrial gene fractions, or poor-quality barcode assignment were excluded. Sample identities were assigned based on cell multiplexing where applicable, and clonal identities were assigned using the PseuMO-Tag Decoder pipeline.

To compare transcriptional components before and after lung colonization, cNMF was performed separately for the original 28-clone BxPC3 dataset, the matched in vitro 12-clone dataset, and the lung-derived dataset, using *k* = 7, *k* = 6, and *k* = 9, respectively. Components identified in these datasets are referred to as BxPC3-GEPs, InVitro-GEPs, and InVivo-GEPs, respectively. Correspondence between components was evaluated using the Jaccard index based on the top 50 genes of each component. The original GEP3 module score was calculated independently using Seurat’s AddModuleScore() function and should not be interpreted as the usage of an InVivo cNMF component.

### Data analysis for drug treatment

For scRNA-seq analysis, data processing, clone demultiplexing (PseuMO-Tag Decoder), and CellPlex sample assignment (Cell Ranger v6.1.2) were performed as described above (Fig. 4). Cells with >20% mitochondrial transcript content, fewer than 200 detected genes, or more than 4,000 detected genes were excluded. Normalization, scaling, PCA, clustering, and UMAP visualization followed the procedures described earlier. The GEP3 module score was calculated for each cell using the AddModuleScore() function in Seurat.

For the scATAC-seq data, processing and analysis were conducted using the ArchR package (v1.0.2) (*61*), following the same workflow described above. Demultiplexing was conducted using the PseuMO-Tag Decoder pipeline. Quality control, dimensionality reduction (LSI), clustering, UMAP projection, peak calling using MACS2, and the addition of peak matrices were performed as previously described.

### Data analysis for clonal heterogeneity

For scRNA-seq data, raw sequencing reads were processed using Cell Ranger (v6.1.2, 10x Genomics) with default parameters and aligned to the GRCh38 human reference genome to generate gene-by-cell expression matrices (Fig. 5). Demultiplexing of individual clones was performed using the PseuMO-Tag Decoder pipeline (see above). Downstream analysis was done using the Seurat R package (v4.0.1) (*56*). Low-quality cells were filtered out based on the following criteria: mitochondrial transcript proportion of >20%, <200 detected genes, or > 4000 detected genes. Gene expression matrices were normalized using Seurat’s NormalizeData() function, which performs log-normalization by scaling each UMI count for each cell to 10,000, followed by natural logarithmic transformation. Data were subsequently scaled using ScaleData(). Highly variable genes were identified using FindVariableFeatures() with the “vst” method, and the top 2000 genes were selected. PCA was performed with RunPCA(). For dimensionality reduction and clustering, the top 30 PCs were used as input for FindNeighbors() and FindClusters() with a resolution of 0.4. UMAP visualization was generated using RunUMAP() based on the same PCs. The knockdown efficiency of the target genes was evaluated by calculating the average expression values for each shRNA group using AverageExpression() and visualized with the ComplexHeatmap package (v2.6.2). Moreover, differentially expressed genes (DEGs) for each clone were identified using Seurat’s FindMarkers() function. To ensure robust detection, an equal number of cells were randomly subsampled 100 times from the scramble control group to match the number of cells per clone. For each iteration, log_2_ fold changes and adjusted *P* values were calculated. Genes with a median log_2_ fold change of ≥0.25 and a median adjusted *P* value of ≤0.1 across iterations were defined as DEGs for that clone. Functional enrichment analysis of the DEGs was done using the Enrichr web server (*57*–*59*), and the enriched pathways were visualized using Appyter available through Enrichr.

For scATAC-seq data, raw reads were processed using the ArchR package (v1.0.2) (*61*) with modifications based on previously described workflows (*46*, *62*). The demultiplexing of individual clones was done using the PseuMO-Tag Decoder pipeline (see above). Analyses were conducted using the hg19 human genome assembly specified with addArchRGenome (“hg19”). Initial quality control was performed with createArrowFiles() to compute the transcription start site (TSS) enrichment scores and fragment counts per nucleus. Cells with TSS enrichment scores <8 or fewer than 2500 unique fragments were excluded. Potential doublets were removed using addDoubletScores() with parameters *k* = 10, knnMethod = “UMAP”, and LSIMethod = 1. Dimensionality reduction was done using addIterativeLSI() based on genome-wide 500-bp tiles. Clustering was performed using addClusters() and Seurat’s FindClusters() function, and the UMAP projection was generated by addUMAP(). To identify reproducible peak regions, pseudo-bulk replicates were generated using addGroupCoverages(), and peak calling was conducted with MACS2 (v2.2.7.1) through addReproduciblePeakSet() with parameters shift = −40, extsize = 80, --nomodel, and --nolambda. Resulting peak set was incorporated into the project using addPeakMatrix(). Marker peaks were identified using getMarkerFeatures() and filtered with getMarkers() under the thresholds *P* value of ≤0.05 and log_2_ fold change of ≥1. TF activity was inferred using chromVAR deviation scores. Motif annotations were added with addMotifAnnotations() using the CIS-BP database, and putative regulatory TFs were inferred based on the overlap between the motif locations and the marker peak regions.

### CUT&Tag data processing

Adapter-trimmed, paired-end reads were aligned to the human reference genome (hg38) using Bowtie2 (v2.4.5) with the parameters: --very-sensitive -X 2000 --no-mixed --no-discordant. The resulting SAM files were converted to BAM format and sorted using SAMtools (v1.15.1). To retain only uniquely mapped reads, the alignments containing the “XS” tag were excluded. PCR duplicates were removed using the MarkDuplicates tool from Picard (v2.27.5) and the options VALIDATION_STRINGENCY = LENIENT and REMOVE_DUPLICATES = true. Reads mapping to genomic blacklist regions (ENCODE hg38 blacklist v2) were removed using intersectBed from the Bedtools suite (v2.30.0) with the corresponding blacklist BED file. Peak calling was performed using MACS2 (v2.2.7.1) in narrowPeak mode with the parameters: --format BAMPE and --qvalue 0.1. To normalize for spike-in controls, reads were also aligned to the *Drosophila melanogaster* reference genome (Dmel_A4_1.0) using the same alignment pipeline. Sample-specific scaling factors were calculated on the basis of mapping percentages. Quantification of the read abundance around the peak centers (±2 kb) identified from the scATAC-seq data was performed using the computeMatrix function of the deepTools package (v3.5.1) (*63*) and applying spike-in–normalized BAM files. The profiles were stratified by genomic region categories (promoter, intronic, exonic, and distal) and visualized using the plotProfile function in deepTools. Peak correlations between samples were calculated using the DiffBind package (v3.12.10), and peak annotations were obtained using the ChIPseeker package (v1.38.0).

### Quantification of the transcriptomic heterogeneity

To determine the effects of EM loss on transcriptomic heterogeneity, we calculated the pairwise NMI scores based on a method previously described by Marjanovic *et al.* (*4*, *38*) and Fennell *et al.* (*4*).

Gene expression values for each clone or condition were discretized into 10 bins using either differentially expressed genes or NMF-derived signature genes as the input feature set. The entropy of an individual cell *X* was defined as follows

$$H\left(X\right)=-\sum_{i=1}^{n}p\left(x_{i}\right)\text{log}p\left(x_{i}\right)$$

Here, *p*(*x_i_*) represents the empirical probability of the expression level of a gene falling into the *i*th bin. To capture the information shared between cell pairs, mutual information was calculated as follows

$$I\left(X;Y\right)=\sum_{x}\sum_{y}p\left(x,y\right)\text{log}\left(\frac{p\left(x,y\right)}{p\left(x\right)p\left(y\right)}\right)$$

Here, *p*(*x*,*y*) denotes the joint distribution of binned gene expression across two cells, and *p*(*x*) and *p*(*y*) are their respective marginal distributions. The NMI between the two cells was then computed as follows

$$\text{NMI}\left(X,Y\right)=\frac{I\left(X;Y\right)}{\sqrt{H\left(X\right)H\left(Y\right)}}$$

To robustly estimate the population-level heterogeneity, 50 cells were randomly subsampled without replacement from each group, and the average pairwise NMI was computed across all cell pairs. This process was repeated 1000 times to minimize sampling bias. NMI distributions were visualized using boxplots, in which the boxes represent the IQR, the center lines indicate the median, and the whiskers extend to 1.5 times the IQR.

To assess interclonal transcriptomic heterogeneity from scRNA-seq data (Fig. 5F), we computed a diversity score following the method described by Ma *et al.* (*36*), with minor modifications. Briefly, PCA was performed on all high-quality cells to reduce dimensionality and extract the major sources of variation. PCA was conducted using the RunPCA function in the Seurat package (v4.0.1) with default parameters. Furthermore, each cell was represented as a vector in the space of the top *n* PCs, and the centroid of the population was defined as the mean vector across all cells. Diversity score was calculated as the average Euclidean distance from each cell to the centroid using the following equation

$$\text{Diversity score}=\frac{1}{m}\sum_{i=1}^{m}\sqrt{\sum_{j=1}^{n}{\left(x_{\mathrm{ij}}-\mu_{j}\right)}^{2}}$$

Here, *m* is the number of cells, *n* is the number of PCs, *x_ij_* is the value of the *j*th PC in the *i*th cell, and μ*_j_* is the mean of the *j*th PC across all cells. To minimize the effect of extreme outliers, cells with values exceeding ±3 SDs from the population mean in any of the first three PCs were excluded.

Diversity scores were calculated separately for each clone and visualized as boxplots. In the boxplots, the lower and upper hinges represent the first and third quartiles, respectively, whereas the horizontal line within the box represents the median. The upper and lower whiskers extend to the highest and lowest values within 1.5 times the IQR from the hinge.

For the interclonal analysis, the top 30 PCs were used for the diversity score calculation. Results were robust to the number of PCs used, with minimal variation observed when adjusting the number of components.

### Quantification of chromatin accessibility heterogeneity in the scATAC-seq data

To quantify the heterogeneity in chromatin accessibility, we calculated the peak entropy for each cCRE. A reproducible peak set was first obtained from the scATAC-seq data using the getMatrixFromProject() function in the ArchR package. Regarding functionally relevant regulatory elements, peaks were filtered to retain only those overlapping with ENCODE cCREs. ENCODE cCRE annotations were downloaded from the UCSC Genome Browser (GRCh38/hg38) and converted to GRCh37/hg19 coordinates using the UCSC LiftOver tool. Tn5 insertion sites were inferred from aligned reads by correcting strand-specific bias: +4 bp for reads mapped to the plus strand and −5 bp for reads on the minus strand (*64*, *65*). Moreover, downstream analyses were restricted to cCRE-overlapping peaks detected in at least 1000 single cells.

To calculate peak entropy, we adapted the single-cell entropy calculation described by Pastore *et al.* (*66*). For each peak, the fraction of positive cells (fpc), defined as the proportion of single cells with at least one Tn5 insertion at that peak, was used to calculate the Shannon entropy as follows

$$\text{Entropy}=-1\times \text{[fpc}\cdot {\text{log}}_{2}\left(\text{fpc}\right)+\left(1-\text{fpc}\right)\cdot {\text{log}}_{2}\left(1-\text{fpc}\right)\text{]}$$

This metric captures the degree of dispersion in chromatin accessibility across the cell population at each cCRE. Peaks with intermediate fpc values (closer to 0.5) yield higher entropy, indicating greater heterogeneity, whereas those with values near 0 or 1 yield lower entropy, reflecting more uniform accessibility.

### Analysis of publicly available PDAC single-cell RNA-seq datasets

Publicly available single-cell RNA-seq datasets from Storrs *et al.* (GSE242230), Peng *et al.* (PRJCA001063), and Zhang *et al.* (GSE197177) were analyzed using Seurat. For GSE242230, malignant cells were selected according to the cell-type annotations provided by the original study. For PRJCA001063, tumor-derived cells annotated as Ductal cell type 1 or Ductal cell type 2 were retained. For GSE197177, cells with 200 to 10,000 detected genes and less than 20% mitochondrial reads were retained, and ductal epithelial cells were selected on the basis of unsupervised clustering and expression of epithelial markers.

GEP3 module scores were calculated using Seurat’s AddModuleScore() function with the top 20 genes of the original BxPC3 GEP3 component. Within each patient or lesion, cells with no detected ARID1A transcript in the normalized expression matrix were classified as ARID1A transcript negative, whereas cells with detectable ARID1A expression were classified as ARID1A transcript positive. The difference in GEP3 module score (ΔGEP3) was defined as the median score in ARID1A transcript-negative cells minus that in ARID1A transcript-positive cells. Differences between the two groups were evaluated using two-sided Wilcoxon rank-sum tests. For GSE197177, matched primary and metastatic lesions from cases 1 to 3 were analyzed separately, and *P* values across the six case-by-site comparisons were adjusted using the Benjamini-Hochberg method.

### Statistical analysis

Statistical analyses were conducted using R software. Parametric tests included unpaired two-tailed Student’s *t* tests and one-way analysis of variance (ANOVA), followed by Dunnett’s or Tukey’s post hoc multiple comparison tests where appropriate. For nonparametric comparisons, the Wilcoxon rank-sum test was used. A *P* value < 0.05 was considered statistically significant.

### Figure preparation

Experimental schematics were created using BioRender and assembled with Microsoft PowerPoint.
