# Supplementary material for: Clonal lineage tracing and parallel multiomics profiling reveal transcriptional heterogeneity induced by ARID1A deficiency
Source: Sci Adv. 2026 Oct 7;12(41):eaed7880. doi: 10.1126/sciadv.aed7880 (PMC13644795; doi:10.1126/sciadv.aed7880)
Supplement: Supplementary file 1 [file sciadv.aed7880_sm.pdf]

Science Advances
AAAS

Supplementary Materials for

Clonal lineage tracing and parallel multiomics profiling reveal transcriptional heterogeneity induced by ARID1A deficiency

Kenichi Miyata *et al.*

Corresponding author: Kenichi Miyata, kenichi.miyata@jfcr.or.jp; Reo Maruyama, reo.maruyama@jfcr.or.jp

Sci. Adv. **12**, eaed7880 (2026)
DOI: 10.1126/sciadv.aed7880

The PDF file includes:

Figs. S1 to S10
Supplementary Text S1 to S3
Legends for tables S1 to S5, S7
Table S6

Other Supplementary Material for this manuscript includes the following:

Tables S1 to S5, S7

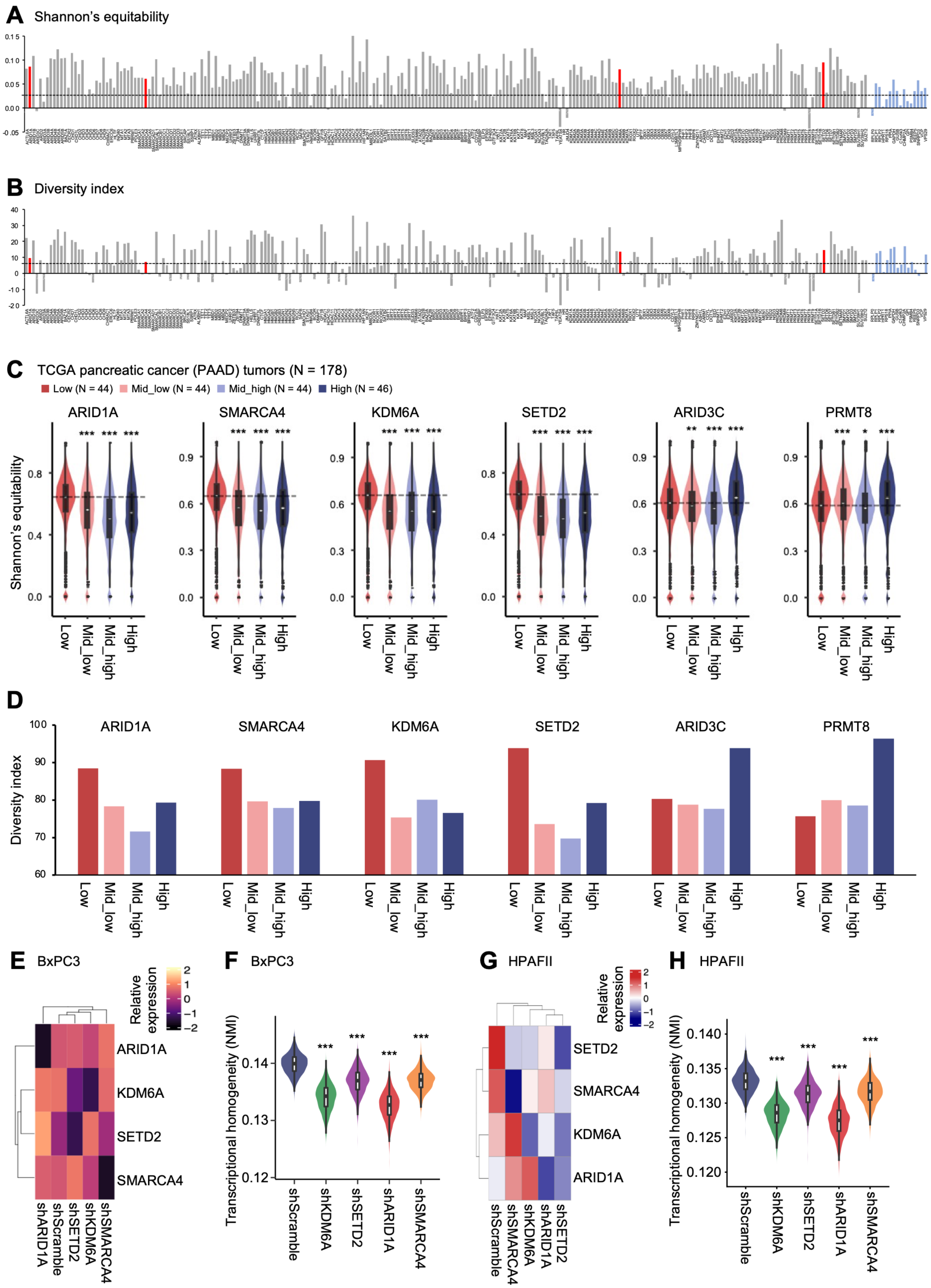
A Shannon's equitability
B Diversity index
C TCGA pancreatic cancer (PAAD) tumors (N = 178)
Low (N = 44) Mid_low (N = 44) Mid_high (N = 44) High (N = 46)
ARID1A
SMARCA4
KDM6A
SETD2
ARID3C
PRMT8
Shannon's equitability
Low
Mid_low
Mid_high
High
D
Diversity index
E BxPC3
Relative expression
ARID1A
KDM6A
SETD2
SMARCA4
shARID1A
shScramble
shSETD2
shKDM6A
shSMARCA4
F BxPC3
Transcriptional homogeneity (NMI)
shScramble
shKDM6A
shSETD2
shARID1A
shSMARCA4
G HPAFII
Relative expression
SETD2
SMARCA4
KDM6A
ARID1A
shScramble
shSMARCA4
shKDM6A
shARID1A
shSETD2
H HPAFII
Transcriptional homogeneity (NMI)
shScramble
shKDM6A
shSETD2
shARID1A
shSMARCA4

Fig. S1. Association between epigenetic modifier expression and transcriptomic heterogeneity in PDAC

(**A** and **B**) Differences in transcriptomic heterogeneity between tumors in the lowest and highest expression quartiles for each gene in the TCGA pancreatic adenocarcinoma cohort (N = 178), quantified using Shannon's equitability index (A) or a PCA-based diversity index (B). Dashed lines indicate the median value for housekeeping genes. Red bars indicate the selected epigenetic modifiers, and blue bars indicate housekeeping genes. (**C** and **D**) Shannon's equitability index (C) and PCA-based diversity index (D) in tumors stratified into four expression quartiles for ARID1A, SMARCA4, KDM6A, SETD2, ARID3C, or PRMT8: Low (N = 44), Mid-low (N = 44), Mid-high (N = 44), and High (N = 46). *P < 0.05, **P < 0.01, and ***P < 0.001 by one-way ANOVA followed by Dunnett's post hoc multiple-comparisons test. (**E** and **G**) Heatmaps of target-gene expression in BxPC3 (E) and HPAFII (G) cells transduced with the indicated shPseuMO-Tag constructs. (**F** and **H**) Transcriptional homogeneity quantified by NMI in BxPC3 (F) and HPAFII (H) cells. For each perturbation, 1,000 bootstrap samples of 50 cells were analyzed. ***P < 0.001 by one-way ANOVA followed by Dunnett's post hoc test.

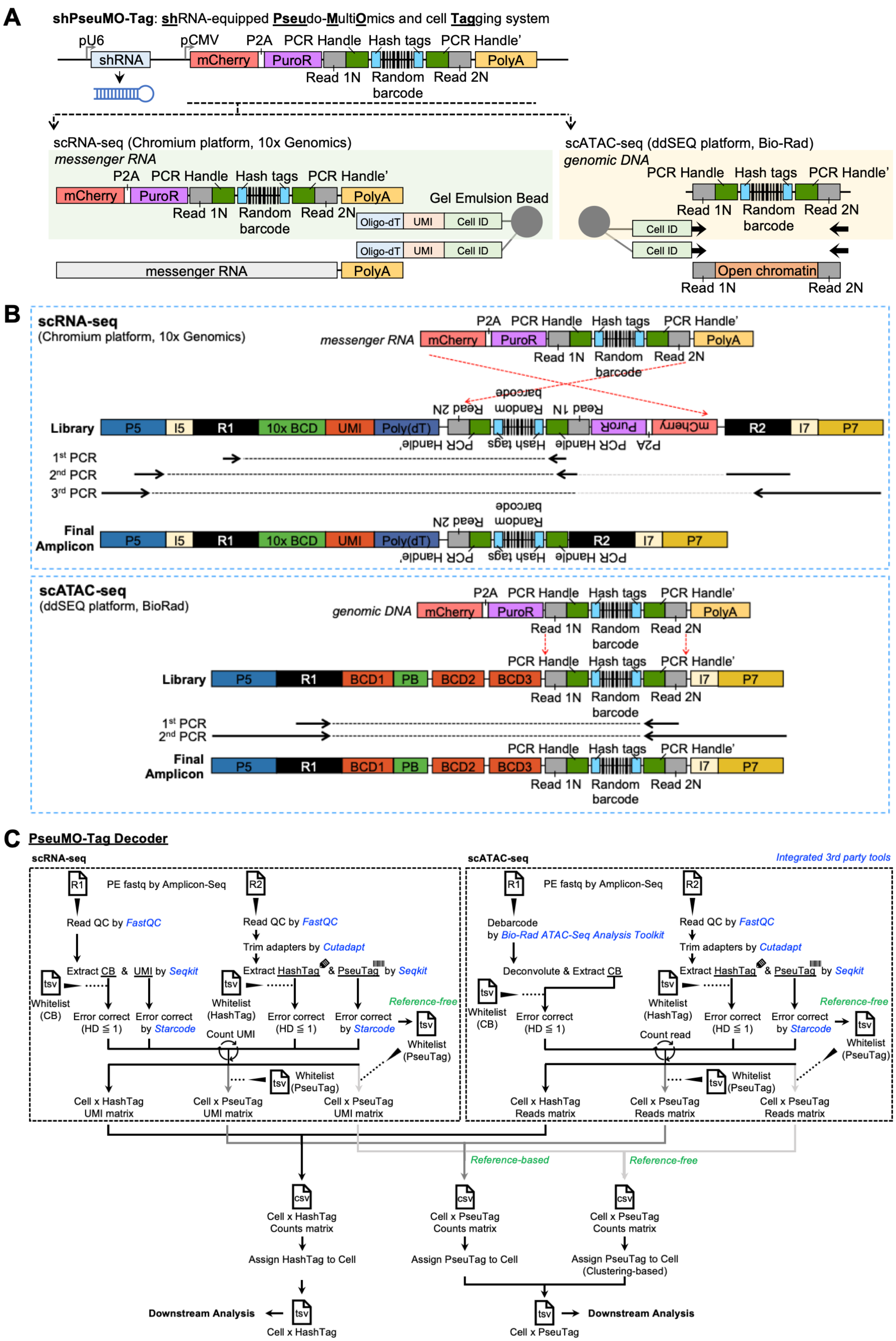
A
shPseuMO-Tag: shRNA-equipped Pseudo-MultiOmics and cell Tagging system
pU6
shRNA
pCMV
mCherry
P2A
PuroR
PCR Handle
Hash tags
PCR Handle'
PolyA
Read 1N
Random barcode
Read 2N
scRNA-seq (Chromium platform, 10x Genomics)
messenger RNA
Gel Emulsion Bead
Oligo-dT
UMI
Cell ID
scATAC-seq (ddSEQ platform, Bio-Rad)
genomic DNA
Open chromatin
B
scRNA-seq
(Chromium platform, 10x Genomics)
Library
P5
I5
R1
10x BCD
UMI
Poly(dT)
R2
I7
P7
1st PCR
2nd PCR
3rd PCR
Final Amplicon
scATAC-seq
(ddSEQ platform, BioRad)
BCD1
PB
BCD2
BCD3
C
PseuMO-Tag Decoder
Integrated 3rd party tools
PE fastq by Amplicon-Seq
Read QC by FastQC
Trim adapters by Cutadapt
Extract CB & UMI by Seqkit
Extract HashTag & PseuTag by Seqkit
Whitelist (CB)
Whitelist (HashTag)
Whitelist (PseuTag)
Error correct (HD ≦ 1)
Error correct by Starcode
Reference-free
Count UMI
Cell x HashTag UMI matrix
Cell x PseuTag UMI matrix
Debarcode by Bio-Rad ATAC-Seq Analysis Toolkit
Deconvolute & Extract CB
Count read
Cell x HashTag Reads matrix
Cell x PseuTag Reads matrix
Reference-based
Cell x HashTag Counts matrix
Cell x PseuTag Counts matrix
Assign HashTag to Cell
Assign PseuTag to Cell
Assign PseuTag to Cell (Clustering-based)
Downstream Analysis
Cell x HashTag
Cell x PseuTag

Fig. S2. Overview of the PseuMO-Tag sequencing strategy and decoding pipeline

(**A**) Schematic of shPseuMO-Tag (shRNA-equipped Pseudo-MultiOmics and cell Tagging), which combines an shRNA cassette with a multiomics-compatible barcode embedded in the 3′ UTR of an mCherry-P2A-PuroR transcript. The barcode contains a random PseuMO-Tag flanked by shRNA-associated HashTags and can be recovered from both scRNA-seq and scATAC-seq libraries. (**B**) Nested-PCR workflows used to enrich PseuMO-Tag-containing reads from Chromium scRNA-seq libraries (top) and ddSEQ scATAC-seq libraries (bottom). (**C**) PseuMO-Tag Decoder workflow for scRNA-seq and scATAC-seq. Cell barcodes, HashTags, and PseuMO-Tags were extracted and error-corrected to generate cell-by-HashTag and cell-by-PseuMO-Tag matrices. PseuMO-Tags were assigned to cells using reference-based or reference-free clone calling for downstream clone-resolved analyses.

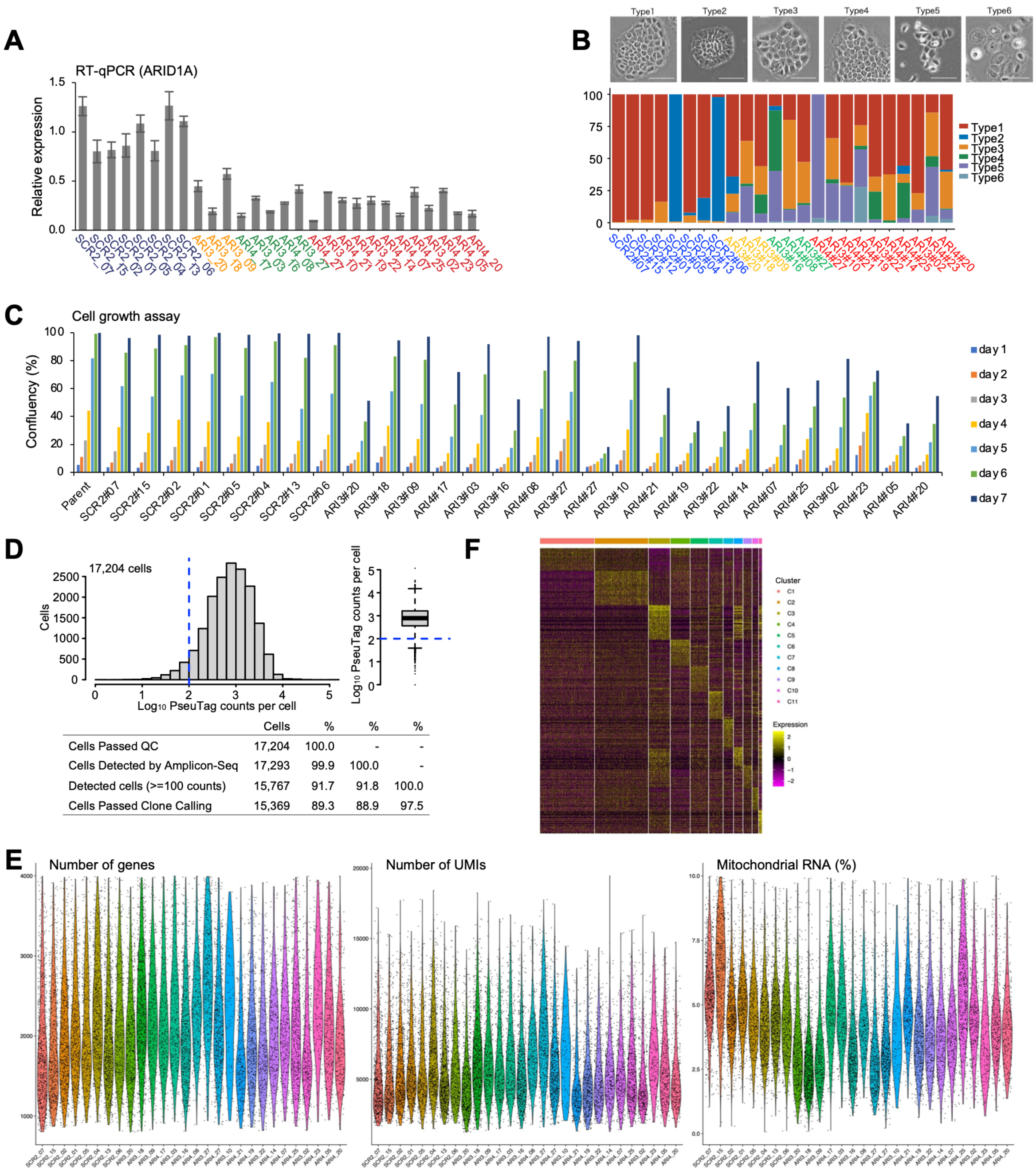

	Cells	%	%	%
Cells Passed QC	17,204	100.0	-	-
Cells Detected by Amplicon-Seq	17,293	99.9	100.0	-
Detected cells (>=100 counts)	15,767	91.7	91.8	100.0
Cells Passed Clone Calling	15,369	89.3	88.9	97.5

Fig. S3. Characterization and scRNA-seq quality metrics of the 28 BxPC3 clones

(**A**) RT-qPCR analysis of ARID1A expression across the 28 clones. Expression was normalized to ACTB and is shown relative to the mean of the control SCR2 clones. Data are mean ± s.d. from three biological replicates. (**B**) Representative phase-contrast images of the six morphological classes and their fractional distribution within each clone. Scale bar, 35 µm. (**C**) Cell growth measured as confluence over 7 days using IncuCyte live-cell imaging. (**D**) PseuMO-Tag-based clone calling for scRNA-seq. The histogram and box plot show log_{10}-transformed PseuMO-Tag counts per cell; the table summarizes amplicon-sequencing detection and clone-assignment rates. (**E**) scRNA-seq quality-control metrics by clone: number of detected genes, number of UMIs, and

percentage of mitochondrial RNA. (**F**) Heatmap of scaled expression of clone-associated differentially expressed genes across single cells; columns are ordered by transcriptomic cluster.

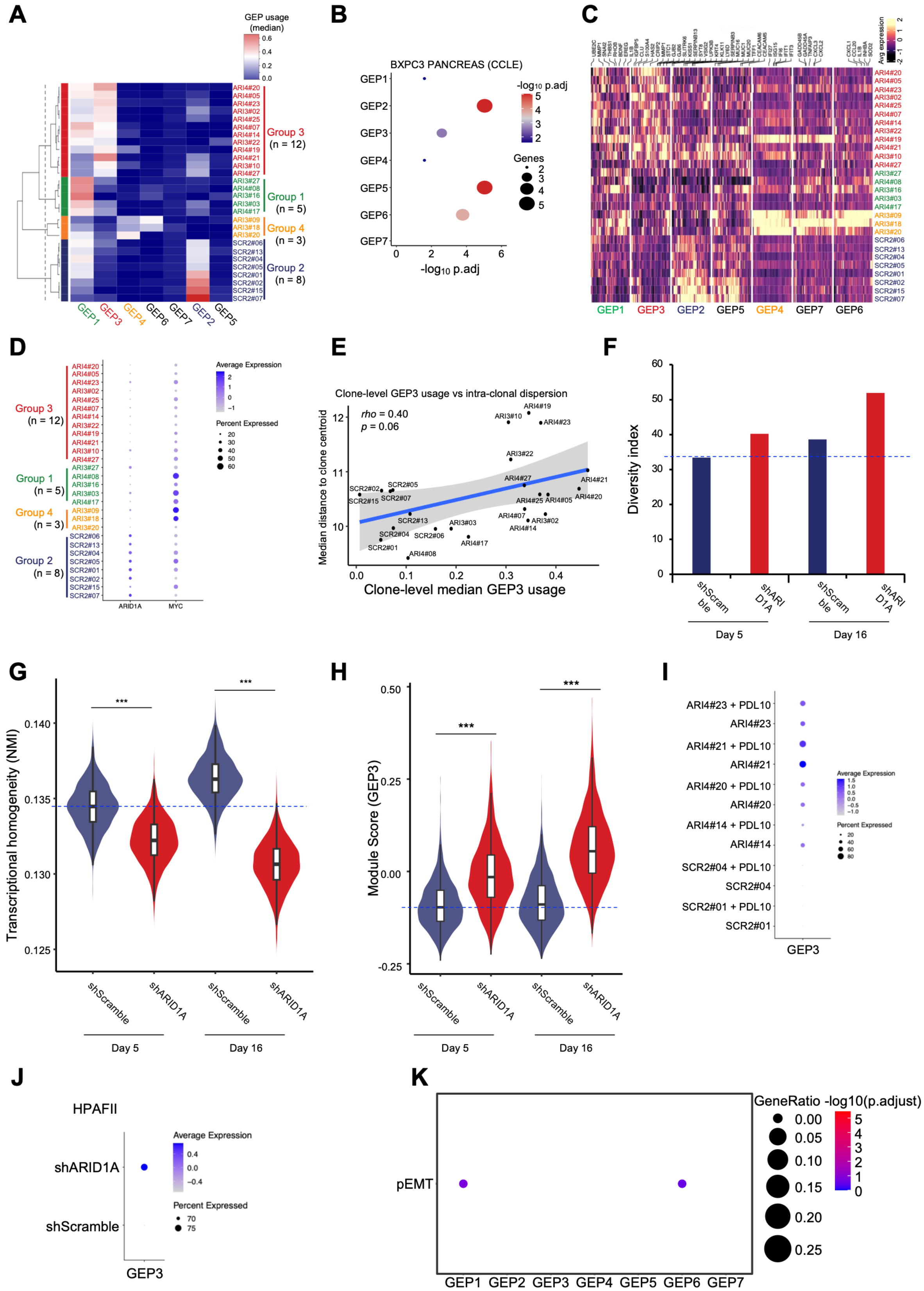
A
GEP usage (median)
Group 3 (n = 12)
Group 1 (n = 5)
Group 4 (n = 3)
Group 2 (n = 8)
GEP1 GEP3 GEP4 GEP6 GEP7 GEP2 GEP5
B
BXPC3 PANCREAS (CCLE)
-log10 p.adj
Genes
C
Avg expression
D
Average Expression
Percent Expressed
ARID1A
MYC
E
Clone-level GEP3 usage vs intra-clonal dispersion
rho = 0.40
p = 0.06
Median distance to clone centroid
Clone-level median GEP3 usage
F
Diversity index
shScramble
shARID1A
Day 5
Day 16
G
Transcriptional homogeneity (NMI)
shScramble
shARID1A
Day 5
Day 16
H
Module Score (GEP3)
I
GEP3
J
HPAFII
shARID1A
shScramble
GEP3
K
GeneRatio -log10(p.adjust)
pEMT
GEP1 GEP2 GEP3 GEP4 GEP5 GEP6 GEP7

Fig. S4. GEP usage and functional associations across the 28 BxPC3 clones

(**A**) Hierarchical clustering of clones based on clone-level median GEP usage. (**B**) Overlap of genes associated with each GEP with BxPC3 marker genes from the Cancer Cell Line Encyclopedia. Dot size indicates the number of overlapping genes, and color indicates the adjusted P value. (**C**) Average expression across clones of the top 50 genes associated with each GEP. (**D**) Expression of ARID1A and MYC across individual clones. Dot size indicates the percentage of expressing cells, and color indicates average expression. (**E**) Association between clone-level median GEP3 usage and clone-level transcriptional dispersion, quantified as the median distance of cells from the clone-specific transcriptional centroid. Each point represents one clone; the line and shaded area show the linear fit and 95% confidence interval. Spearman $\rho = 0.398$, $P = 0.061$. (**F**) PCA-based transcriptional diversity index at days 5 and 16 after transduction with shScramble or shARID1A. (**G**) Transcriptional homogeneity quantified by NMI at days 5 and 16. Each violin represents 1,000 bootstrap samples of 50 cells. ***$P < 0.001$ by one-way ANOVA followed by Tukey-Kramer's post hoc test. (**H**) GEP3 module scores at days 5 and 16 after transduction. ***$P < 0.001$ by one-way ANOVA followed by Tukey-Kramer's post hoc test. (**I**) Expression of representative GEP3-associated genes in selected BxPC3 clones at the initial analysis and after an additional 10 population doublings (PDL10). Dot size indicates the percentage of expressing cells, and color indicates average expression. (**J**) GEP3 module scores in pooled HPAFII cells transduced with shScramble or shARID1A. (**K**) Enrichment of a curated partial-EMT gene set from Werba et al. across GEP1–GEP7. Dot size indicates gene ratio, and color indicates the adjusted P value.

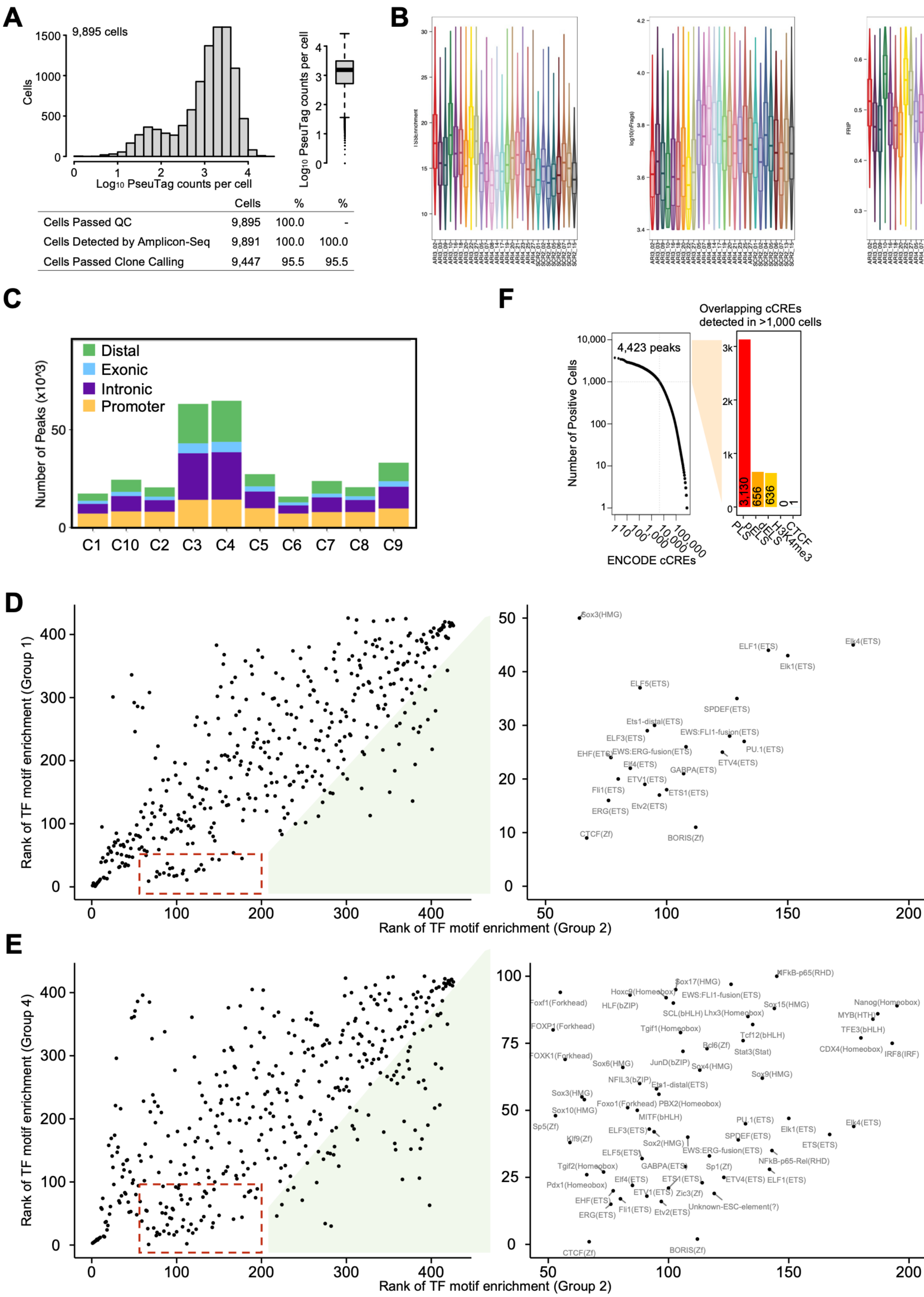

Fig. S5. Chromatin-accessibility profiling and motif analysis of the 28 BxPC3 clones

(**A**) PseuMO-Tag-based clone calling for scATAC-seq. The histogram and box plot show $\log_{10}$-transformed PseuMO-Tag counts per cell; the table summarizes amplicon-sequencing detection and clone-assignment rates. (**B**) scATAC-seq quality-control metrics by clone: number of

fragments per cell, TSS enrichment score, and fraction of reads in peaks (FRiP). (**C**) Numbers of accessible peaks assigned to promoter, intronic, exonic, or distal regions within each chromatin cluster. (**D** and **E**) Comparison of transcription factor motif-enrichment ranks between Group 2 (x axis) and Group 1 (D) or Group 4 (E) (y axis), with motifs ranked by hypergeometric-test P value. Insets show motifs with relatively high enrichment in the indicated ARID1A-knockdown group. (**F**) Number of cells with accessible signal at each ENCODE cCRE (left) and annotation-class distribution of the 4,423 cCRE-overlapping peaks detected in more than 1,000 cells (right).

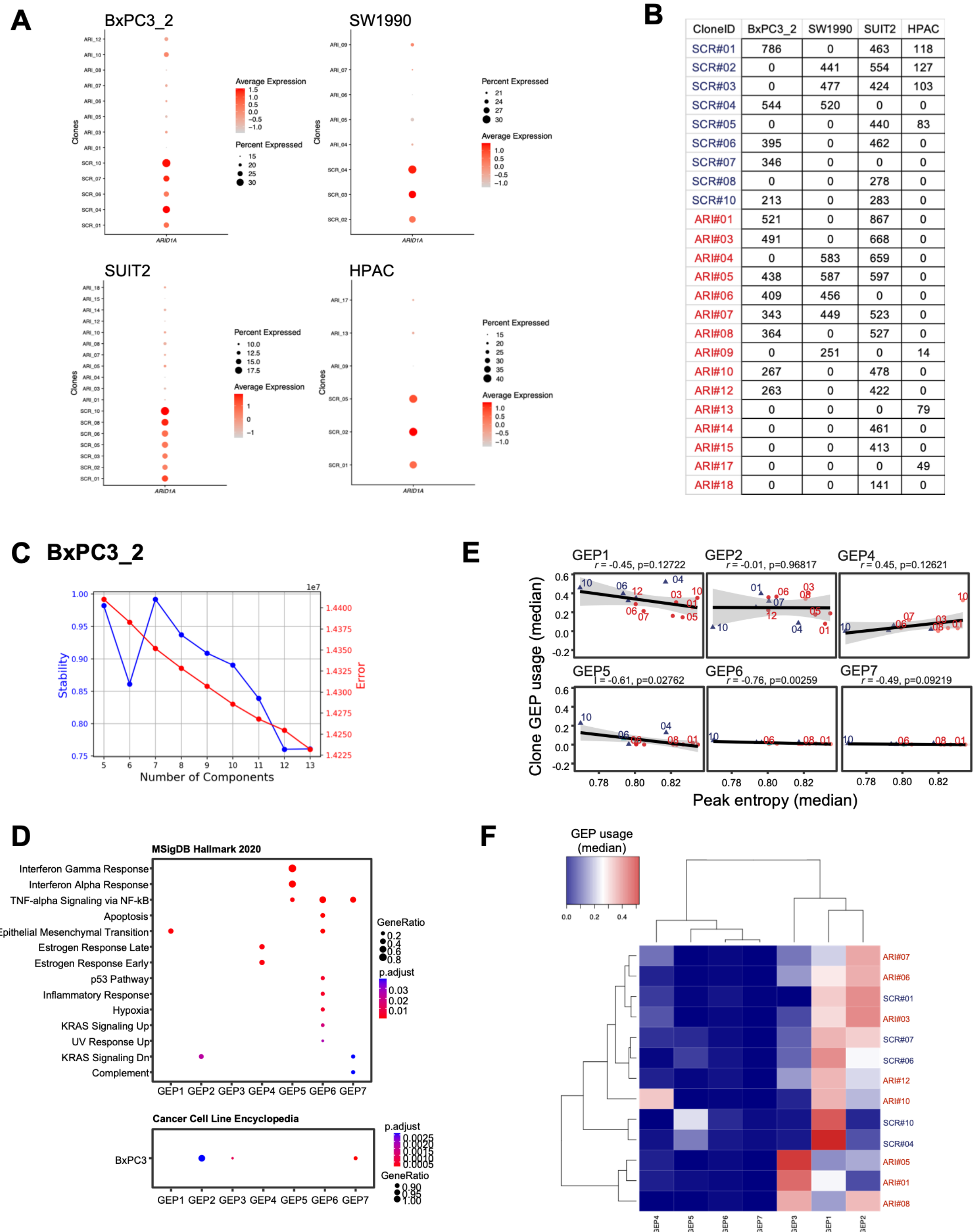

CloneID	BxPC3_2	SW1990	SUIT2	HPAC
SCR#01	786	0	463	118
SCR#02	0	441	554	127
SCR#03	0	477	424	103
SCR#04	544	520	0	0
SCR#05	0	0	440	83
SCR#06	395	0	462	0
SCR#07	346	0	0	0
SCR#08	0	0	278	0
SCR#10	213	0	283	0
ARI#01	521	0	867	0
ARI#03	491	0	668	0
ARI#04	0	583	659	0
ARI#05	438	587	597	0
ARI#06	409	456	0	0
ARI#07	343	449	523	0
ARI#08	364	0	527	0
ARI#09	0	251	0	14
ARI#10	267	0	478	0
ARI#12	263	0	422	0
ARI#13	0	0	0	79
ARI#14	0	0	461	0
ARI#15	0	0	413	0
ARI#17	0	0	0	49
ARI#18	0	0	141	0

Fig. S6. Independent BxPC3 replication and clone recovery across PDAC cell lines

(**A**) ARID1A expression across shPseuMO-Tag-labeled clones from the independent BxPC3 experiment (BxPC3_2), SW1990, SUIT-2, and HPAC cells. Dot size indicates the percentage of expressing cells, and color indicates average expression. (**B**) Numbers of cells assigned to each clone after quality filtering and clone calling. (**C** to **F**) Analysis of the independent BxPC3 experiment. (C) cNMF k-selection plot used to determine the factorization rank. (D) Enrichment of MSigDB Hallmark 2020 and Cancer Cell Line Encyclopedia gene sets among genes associated with each component. Dot size indicates gene ratio, and color indicates the adjusted P value. (E)

Correlations between clone-level median component usage and median peak entropy. Pearson correlation coefficients and P values are indicated. (F) Hierarchical clustering of clones based on clone-level median component usage; inferred functional annotations are shown below the heatmap.

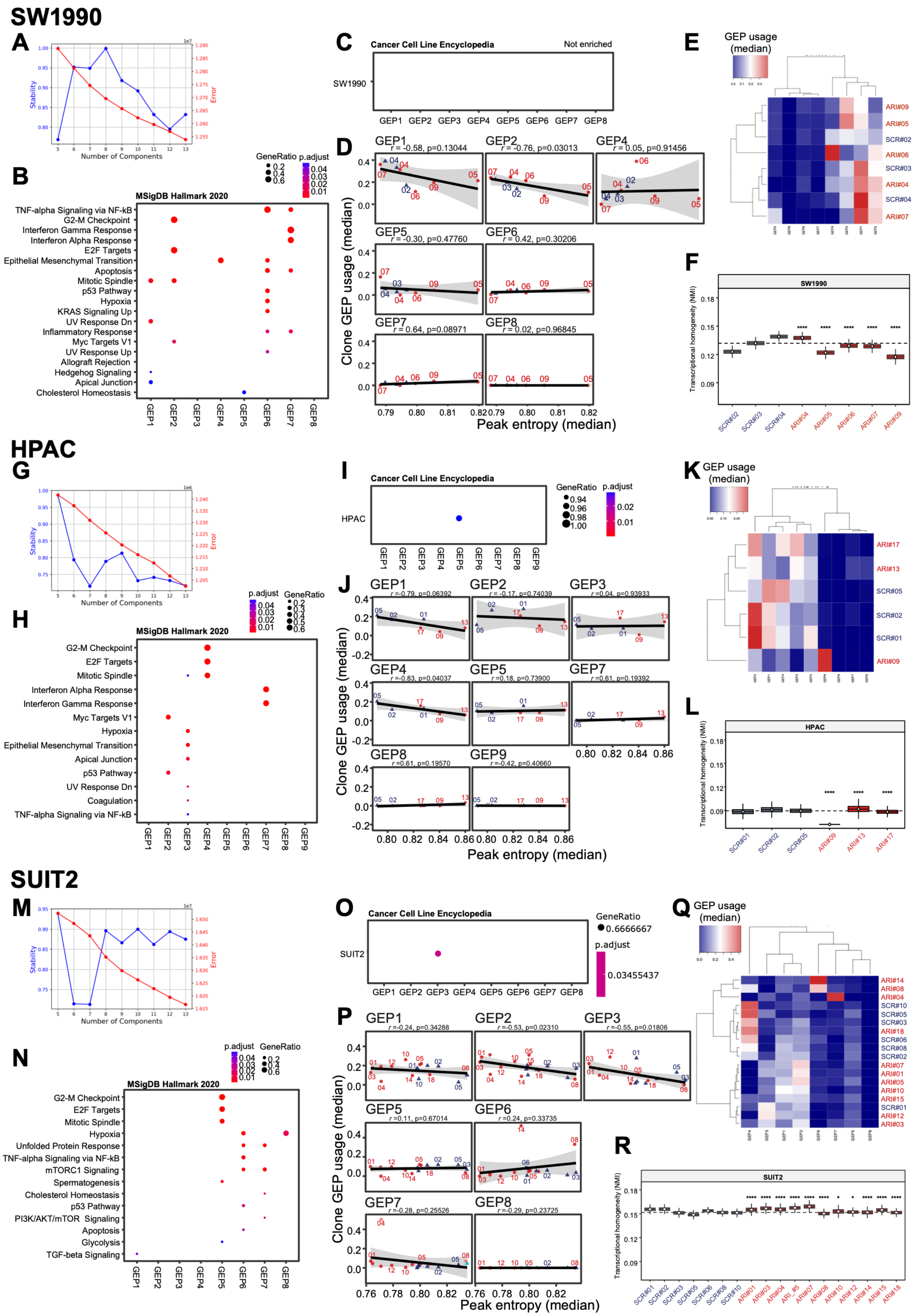

SW1990
A
Stability
Error
Number of Components
B
GeneRatio
p.adjust
MSigDB Hallmark 2020
TNF-alpha Signaling via NF-kB
G2-M Checkpoint
Interferon Gamma Response
Interferon Alpha Response
E2F Targets
Epithelial Mesenchymal Transition
Apoptosis
Mitotic Spindle
p53 Pathway
Hypoxia
KRAS Signaling Up
UV Response Dn
Inflammatory Response
Myc Targets V1
UV Response Up
Allograft Rejection
Hedgehog Signaling
Apical Junction
Cholesterol Homeostasis
C
Cancer Cell Line Encyclopedia
Not enriched
SW1990
D
Clone GEP usage (median)
Peak entropy (median)
E
GEP usage (median)
F
SW1990
Transcriptional homogeneity (NMI)
HPAC
G
H
MSigDB Hallmark 2020
G2-M Checkpoint
E2F Targets
Mitotic Spindle
Interferon Alpha Response
Interferon Gamma Response
Myc Targets V1
Hypoxia
Epithelial Mesenchymal Transition
Apical Junction
p53 Pathway
UV Response Dn
Coagulation
TNF-alpha Signaling via NF-kB
I
Cancer Cell Line Encyclopedia
HPAC
J
K
GEP usage (median)
L
HPAC
SUIT2
M
N
MSigDB Hallmark 2020
G2-M Checkpoint
E2F Targets
Mitotic Spindle
Hypoxia
Unfolded Protein Response
TNF-alpha Signaling via NF-kB
mTORC1 Signaling
Spermatogenesis
Cholesterol Homeostasis
p53 Pathway
PI3K/AKT/mTOR Signaling
Apoptosis
Glycolysis
TGF-beta Signaling
O
Cancer Cell Line Encyclopedia
SUIT2
GeneRatio
0.6666667
p.adjust
0.03455437
P
Q
GEP usage (median)
R
SUIT2

Fig. S7. Cross-cell-line assessment of signature-poor cNMF components after ARID1A knockdown

(**A** to **F**) Analysis of SW1990 clones. (A) cNMF k-selection plot. (B and C) Enrichment of MSigDB Hallmark 2020 (B) and Cancer Cell Line Encyclopedia (C) gene sets among genes associated with each component. (D) Correlations between clone-level median component usage and median peak entropy. Pearson correlation coefficients and P values are indicated. (E) Hierarchical clustering of clones based on clone-level median component usage; inferred functional annotations are shown below the heatmap. (F) Transcriptional homogeneity quantified by NMI for individual clones. (**G** to **L**) Corresponding analyses of HPAC clones. (**M** to **R**) Corresponding analyses of SUIT-2 clones. In (F), (L), and (R), dashed lines indicate the median of the control clones. *$P < 0.05$, **$P < 0.01$, ***$P < 0.001$, and ****$P < 0.0001$ by one-way ANOVA followed by Dunnett's post hoc test; NS, not significant.

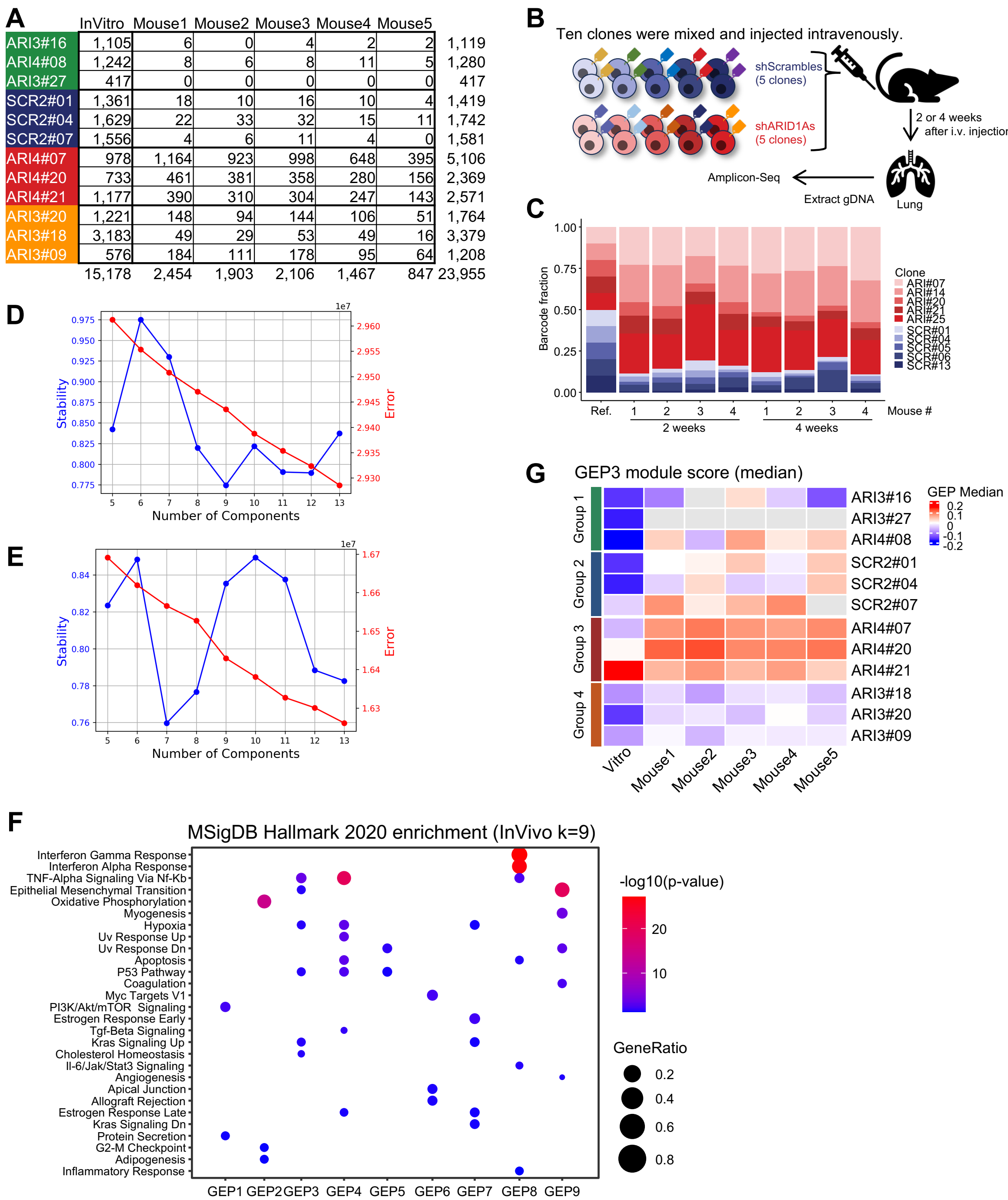

Fig. S8. Single-cell characterization of lung-colonizing BxPC3 clones

(**A**) Numbers of scRNA-seq cells assigned to each clone in the matched pre-injection 12-clone pool and in lung-derived samples from five individual mice. Clones are grouped according to the four transcriptional groups defined in the original BxPC3 experiment. (**B** and **C**) Independent 10-clone lung-colonization assay. (B) Five control and five ARID1A-knockdown clones were mixed and injected intravenously into NSG mice; lungs were collected after 2 or 4 weeks for barcode analysis. (C) Clonal composition of the input population (Ref) and lung-derived populations from

four mice per time point. The schematic in (B) was created by the authors using Microsoft PowerPoint. (**D** and **E**) cNMF k-selection plots for the matched pre-injection dataset (D) and lung-derived dataset (E). (**F**) Enrichment of MSigDB Hallmark 2020 gene sets among genes associated with InVivo-GEP1–GEP9. Dot size indicates gene ratio, and color indicates $-\log_{10}$(P value). (**G**) Median module score calculated using the original BxPC3 GEP3 gene set across clones in the matched pre-injection and lung-derived datasets.

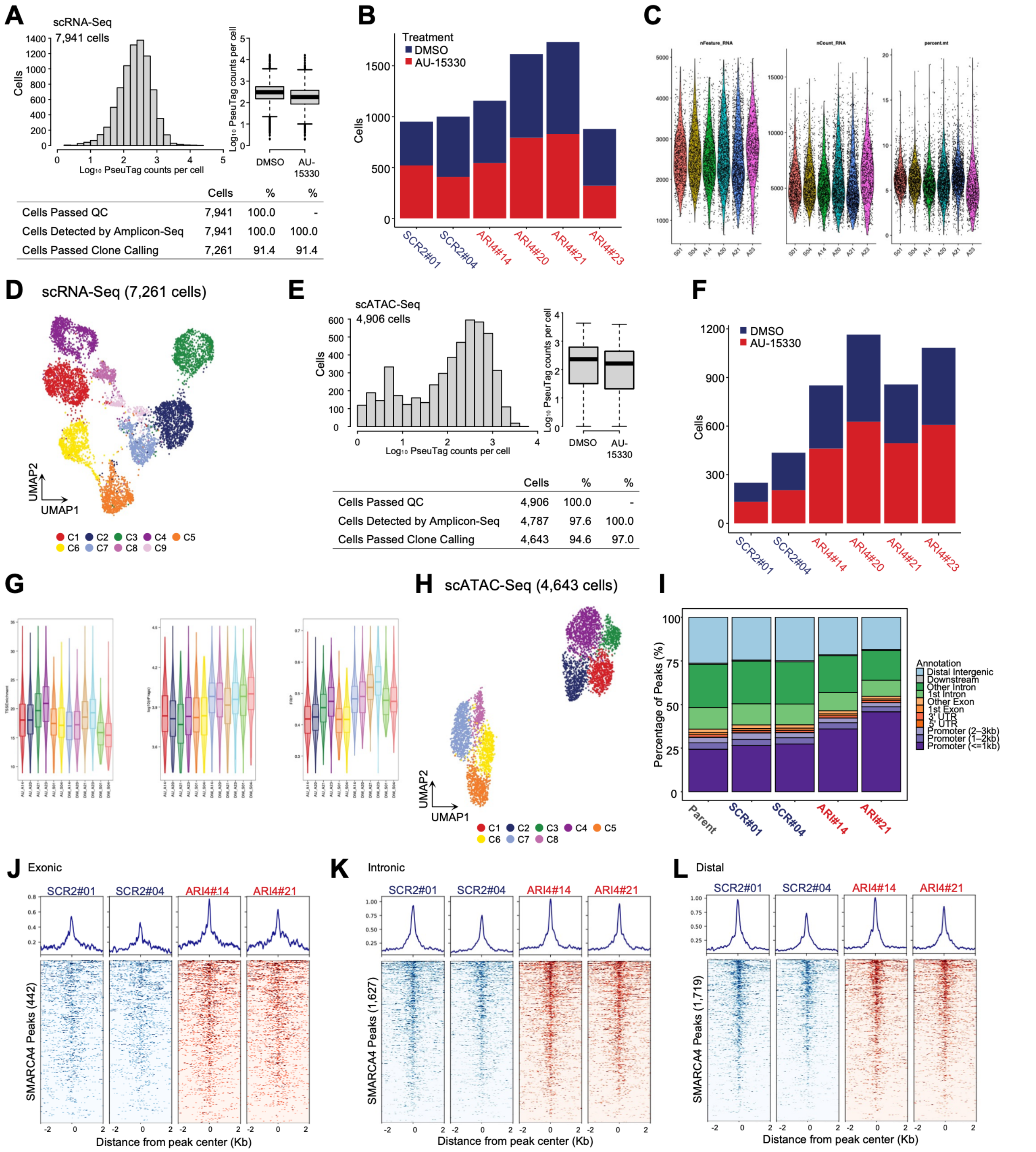

Fig. S9. Quality control and single-cell profiling of BxPC3 clones after SMARCA4 degradation

(**A** and **E**) PseuMO-Tag-based clone calling for scRNA-seq (A; 7,941 cells passing QC) and scATAC-seq (E; 4,906 cells passing QC) after DMSO or AU-15330 treatment. Histograms and box plots show $\log_{10}$-transformed PseuMO-Tag counts per cell; tables summarize amplicon-sequencing detection and clone-assignment rates. (**B** and **F**) Numbers of cells retained for downstream scRNA-seq (B) and scATAC-seq (F), stratified by clone and treatment. (**C** and **G**)

Quality-control metrics for scRNA-seq (C; detected genes, UMI counts, and mitochondrial RNA percentage) and scATAC-seq (G; fragments per cell, TSS enrichment, and FRiP). (**D** and **H**) UMAP projections of scRNA-seq (D) and scATAC-seq (H) cells, colored by unsupervised cluster. (**I**) Genomic annotation of SMARCA4 CUT&Tag peaks in parental BxPC3 cells and the indicated control and ARID1A-knockdown clones. (**J** to **L**) Spike-in-normalized SMARCA4 CUT&Tag signal profiles and heatmaps centered on peaks enriched in ARI4#21 and classified as exonic (J), intronic (K), or distal (L).

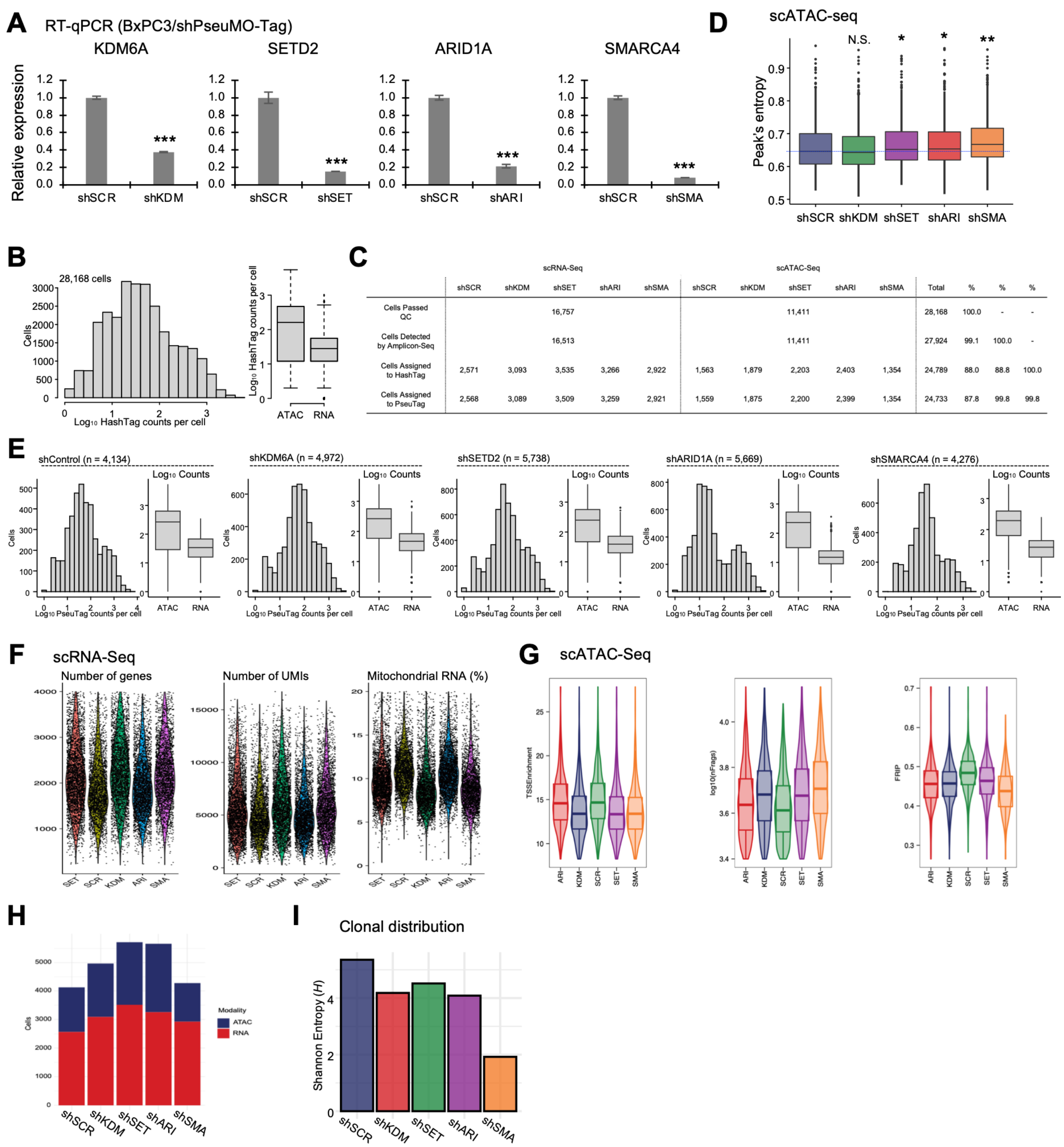

Fig. S10. Quality control and clonal distributions in the large-scale pooled experiment

(**A**) RT-qPCR analysis of KDM6A, SETD2, ARID1A, and SMARCA4 expression in BxPC3 cells transduced with the corresponding shPseuMO-Tag vectors or a nontargeting control. Expression was normalized to ACTB. Data are mean ± s.d. from three biological replicates. ***$P < 0.001$ by two-tailed Student's t test. (**B**) Distribution of $\log_{10}$-transformed HashTag counts per cell across the combined scRNA-seq and scATAC-seq datasets. The box plot compares HashTag counts between modalities. (**C**) Summary of quality-control, HashTag-assignment, and PseuMO-Tag-assignment metrics for each shRNA condition and modality. (**D**) Peak-entropy distributions across shRNA conditions, calculated at cCRE-overlapping accessible peaks. The dashed line indicates

the median of the control condition. *P < 0.05 and **P < 0.01 by one-way ANOVA followed by Dunnett's post hoc test; NS, not significant. (**E**) Distributions of $\log_{10}$-transformed PseuMO-Tag counts per cell for each shRNA condition in scRNA-seq and scATAC-seq. (**F**) scRNA-seq quality-control metrics by shRNA condition: number of detected genes, number of UMIs, and percentage of mitochondrial RNA. (**G**) scATAC-seq quality-control metrics by shRNA condition: TSS enrichment, number of fragments per cell, and FRiP. (**H**) Numbers of scRNA-seq and scATAC-seq cells assigned to each shRNA condition. (**I**) Shannon entropy of clone-frequency distributions across shRNA conditions.

Supplementary Text

Supplementary Text 1. IMPACH enables clone-resolved analysis across complementary experimental scales

Dissecting how a defined molecular perturbation reshapes transcriptional and chromatin-accessibility profiles requires perturbation identity, clonal ancestry, and single-cell molecular measurements to be linked within a common experimental framework. We therefore developed the integrated multimodal platform for analyzing clonal heterogeneity (IMPACH), which combines perturbation-coupled cellular barcoding, clonal expansion, parallel single-cell profiling, and downstream functional interrogation. The shPseuMO-Tag vector encodes both an shRNA identity and a heritable cellular barcode that can be recovered from scRNA-seq and scATAC-seq libraries, allowing each profiled cell to be assigned to its perturbation condition and clonal lineage. The scRNA-seq and scATAC-seq measurements were generated from separate aliquots of the same cellular mixture rather than from the same individual cells. IMPACH therefore provides clone-resolved pseudo-multiomic integration: transcriptomic and chromatin-accessibility profiles are connected through their shared clonal identities, but the two modalities are not simultaneously measured in the same cell. Pooling the clonal populations before library preparation reduces technical variation among clones and permits multiple lineages to be compared under matched culture, processing, and sequencing conditions.

IMPACH can be configured at different experimental resolutions according to the biological question. In the single-cell-cloning configuration used for the 28-clone experiment (Figs. 1 to 4), each clone was operationally defined as the descendants of a single founder cell isolated after shPseuMO-Tag transduction. All cells within a clone therefore shared a common post-perturbation ancestor at the time of isolation. Expansion of a limited number of clones provided sufficient cellular depth to examine variation within each single-cell-derived population while also comparing independently derived lineages exposed to the same perturbation.

Analyzing multiple independent clones is important because the outcome of ARID1A depletion may depend on the transcriptional and chromatin context of the founder cell, as well as on changes acquired during subsequent expansion. Features recurring across several independently derived clones are therefore less likely to reflect an idiosyncratic property of one founder lineage. Conversely, clone-specific outcomes remain biologically informative because they reveal that a shared perturbation does not necessarily impose a uniform transcriptional response.

A practical advantage of the single-cell-cloning configuration is that the physically expanded populations remain available after destructive single-cell profiling. Clones selected on the basis of their molecular features can therefore be returned to for functional and mechanistic experiments. In this study, characterized clones were remixed at defined proportions and subjected to environmental stress or lung-colonization assays (Fig. 3), and selected Group 2 and Group 3 clones were used to test the effects of SMARCA4 degradation (Fig. 4). The selected Group 3 clones were chosen on the basis of high GEP3 usage and/or elevated peak entropy together with confirmed ARID1A knockdown. These focused experiments were designed to interrogate clones displaying the features of interest and were not intended to establish that the selected clones represented the full range of Group 3 behavior.

Re-barcoding an established clone provides a finer level of lineage resolution within a previously isolated population. In the re-barcoding experiment, descendants of SCR2#04 or ARI4#21 were marked with additional barcodes before in vivo selection, allowing barcode-defined sublineage complexity to be compared after lung colonization (Fig. 3, G to M). This approach tracks the relative representation of descendant sublineages, but it does not directly reveal the transcriptional

state of the individual cell that initiates or survives colonization. Greater retained barcode diversity should therefore not, by itself, be interpreted as direct evidence that a broader range of transcriptional states was maintained.

When broad clonal coverage is prioritized over deep cellular sampling of each lineage, the single-cell-isolation step can be omitted. In the configuration used in Fig. 5, approximately 400 independently transduced founder cells were seeded for each perturbation and expanded as pooled populations, enabling the simultaneous analysis of approximately 2,000 barcode-defined clones across five perturbation conditions. This configuration provided broad coverage of clonal abundance and perturbation-associated variation, although fewer cells were recovered from each individual lineage and the physical clones were not separately preserved for targeted follow-up.

The 28-clone and approximately 2,000-clone configurations should not be viewed as measuring fundamentally distinct biological levels of heterogeneity. The definition of a clone depends on when a lineage is marked or isolated and on the duration of subsequent expansion. In both configurations, a clone is an experimentally defined population descended from a marked founder cell. The two designs instead provide complementary resolutions: the limited-clone design offers greater cellular depth and experimental tractability for selected lineages, whereas the pooled design provides broader coverage of independently perturbed founders.

This distinction is also important when interpreting selection. IMPACH identifies which barcode-defined clones become enriched or depleted, but it does not determine which transcriptional profile within a clone was directly selected. For example, preferential recovery of clones with high baseline GEP3 usage does not demonstrate that individual cells with high GEP3 usage initiated lung colonization or survived a particular stress. Such conclusions would require lineage and molecular-state measurements at a finer temporal and cellular resolution than provided here.

Together, these complementary configurations allow IMPACH to link a defined molecular perturbation with clonal ancestry, transcriptomic and chromatin-accessibility profiles, clonal abundance, and experimentally testable clone-level phenotypes. Its main value lies not in imposing a fixed hierarchy of heterogeneity, but in allowing the experimental resolution to be adjusted from broad clonal screening to detailed molecular and functional interrogation.

Supplementary Text 2. Analytical characterization of GEP3 as a recurrent low-coherence component

GEP3 was recurrently detected across ARID1A-deficient clones but, unlike several of the other cNMF-derived components, lacked a dominant pathway annotation. We retained the designation GEP3 for continuity with the original analysis, but this label should not be taken to imply that GEP3 represents a conventional coordinated gene-expression program. We therefore examined whether cells with high GEP3 usage shared the internal coherence expected of a discrete transcriptional state.

Cells with high GEP3 usage showed substantially lower cell-cell similarity than cells with high usage of more coherent components, such as GEP4 (Fig. 1I). The top-ranked genes associated with GEP3 also showed comparatively weak gene-gene coexpression across cells with high GEP3 usage (Fig. 1J). Moreover, gene-module coherence remained low across progressively higher GEP3 usage bins and did not increase in the highest-usage cells (Fig. 1K). Thus, increasing GEP3 usage was not accompanied by convergence toward a shared expression profile. These findings argue against interpreting cells with high GEP3 usage as a discrete cellular state defined by a coordinated molecular signature.

We next examined the relationship between GEP3 usage and the breadth of transcriptional profiles within individual clonal populations. Clone-level median GEP3 usage tended to be positively associated with transcriptional dispersion, quantified as the distance of individual cells from their clone-specific transcriptional centroid (Spearman's $\rho = 0.398$, $P = 0.061$; fig. S4E). Although this nominal association did not reach conventional statistical significance, its direction was consistent with GEP3 usage reflecting the breadth of transcriptional profiles occupied within a clone rather than the abundance of one defined state.

Because matrix factorization can generate poorly annotated or residual components, we further tested whether the observed properties of GEP3 could arise from NMF applied to data lacking cell-level biological structure. Independently permuting the expression of each gene across cells disrupted gene-gene coexpression, cell-level expression structure, and associations with clonal identity while preserving the marginal expression distribution of each gene. NMF factors derived from these expression-shuffled matrices therefore provided a null distribution against which the observed GEP3 component could be evaluated.

The cell-cell similarity of the observed GEP3 component was substantially lower than that of factors recovered from the expression-shuffled data (observed GEP3, 0.163; null median, 0.503; empirical $P = 0.0047$; Fig. 1L). In addition, the association between observed GEP3 usage and clone-level transcriptional dispersion was stronger than expected among the null factors (observed Spearman's $\rho = 0.398$; empirical $P = 0.033$; Fig. 1M). The combination of unusually low cell-level coherence and coupling to clone-level transcriptional dispersion was therefore not typically reproduced by factorization of the shuffled data.

These analyses do not assign GEP3 to a single molecular pathway, nor do they establish GEP3 itself as a causal determinant of transcriptional diversification. Rather, they support an operational interpretation of GEP3 as a recurrent, low-coherence cNMF-derived component associated with heterogeneous transcriptional diversification following ARID1A loss. Under this interpretation, GEP3 usage summarizes an axis along which cells and clones differ in transcriptional variability; it does not quantify the abundance of a discrete cell state or the activity of a conventional coordinated transcriptional program.

Accordingly, throughout the manuscript we distinguish among GEP3 usage, cells with high GEP3 usage, and GEP3-enriched clones. These terms refer, respectively, to the cNMF-derived usage value, cells positioned strongly along this variability-associated axis, and clones with relatively high median usage. They should not be interpreted as defining a fixed GEP3 state, a stable GEP3-positive subpopulation, or a single GEP3-driven phenotype.

Supplementary Text 3. Reproducibility and cell-line dependence of ARID1A-associated transcriptional diversification

To assess the reproducibility and cell-line dependence of ARID1A-associated transcriptional diversification, the clone-resolved shPseuMO-Tag workflow was repeated in an independent BxPC3 experiment and applied to SW1990, HPAC, and SUIT-2 cells (Fig. 2, I and J; figs. S6 and S7). HPAFII cells were examined separately as a pooled population because stable single-cell-derived clones could not be established. The HPAFII analysis therefore tested whether the original BxPC3 GEP3 gene set responded to ARID1A knockdown, but it did not provide a clone-resolved assessment of GEP structure or its relationship with chromatin-accessibility entropy.

cNMF was performed independently for each clone-resolved dataset. Component numbering was therefore dataset-specific, and similarly numbered components in different cell lines should not be assumed to represent identical molecular programs. Components were compared on the basis of

their constituent genes, pathway annotations, transcriptional homogeneity, and relationships with clone-level chromatin-accessibility entropy. Because GEP3 lacks a defining pathway signature, correspondence across datasets necessarily rests more heavily on shared statistical properties than on conservation of a specific gene set.

In the independent BxPC3 experiment, a subset of ARID1A-deficient clones again showed high usage of a signature-poor component, BxPC3_2 GEP3. This component lacked a dominant MSigDB Hallmark annotation and was associated with elevated peak entropy and reduced transcriptional homogeneity (Fig. 2, I and J; fig. S6). These observations reproduced the principal properties of the original GEP3 component in an independently transduced, cloned, expanded, and profiled set of BxPC3 lineages.

The independent BxPC3 experiment also recovered EMT-like and interferon-like components. However, unlike in the original experiment, these components were detected in control as well as ARID1A-deficient clones. Their occurrence in control lineages indicates that these transcriptional profiles were not created exclusively by ARID1A depletion. A more cautious interpretation is that ARID1A loss alters the relative accessibility, usage, or distribution of profiles already available within the parental population, while also favoring the emergence of less readily annotated variability-associated components in a subset of lineages.

In SW1990 cells, a signature-poor component was likewise associated with elevated peak entropy. The genes contributing to the SW1990 component were not identical to those defining the original BxPC3 GEP3 component. This difference is compatible with the interpretation that the recurring feature is a mode of transcriptional variability rather than one conserved gene-expression module. Nevertheless, because the correspondence is based on shared statistical and epigenomic properties rather than direct identity of the constituent genes, the BxPC3 and SW1990 components should be regarded as related rather than equivalent.

In HPAC cells, GEP6 lacked a dominant Hallmark annotation but did not show a significant overall association with peak entropy. One ARID1A-deficient clone showed relatively high values for both GEP6 usage and peak entropy, but this observation was insufficient to establish a general clone-level relationship. The smaller number of informative cells and clones may have limited statistical power; alternatively, the signature-poor HPAC component may differ biologically from the variability-associated components observed in BxPC3 and SW1990. The HPAC result should therefore be regarded as suggestive but inconclusive.

SUIT-2 cells showed a qualitatively different response. ARID1A knockdown was accompanied by increased peak entropy but greater, rather than reduced, transcriptional homogeneity. This uncoupling demonstrates that increased variability in chromatin accessibility does not necessarily produce increased transcriptional heterogeneity in every cellular background. The mechanisms responsible for this difference were not examined directly, and the SUIT-2 result therefore emphasizes that the transcriptional consequences of ARID1A depletion depend on cellular context.

In HPAFII cells, ARID1A knockdown increased the module score calculated from the original BxPC3 GEP3 gene set (fig. S4J). This observation supports the detectability of GEP3-associated expression outside BxPC3. However, because HPAFII cells were analyzed as pooled populations rather than independently derived clones, the result does not establish the recurrence of a low-coherence cNMF component, increased clone-level transcriptional dispersion, or coupling between GEP3 usage and peak entropy in HPAFII.

Taken together, the cross-dataset results support reproducibility at two different levels. First, the main properties of GEP3 were recovered in a fully independent BxPC3 experiment. Second, signature-poor components associated with chromatin-accessibility variability were observed in

some additional pancreatic cancer backgrounds, most clearly in SW1990. The results were weaker or qualitatively different in HPAC and SUIT-2, indicating that this response is not universal.
Accordingly, the recurrent feature should not be described as induction of one conserved GEP3 program across all cell lines. A more appropriate interpretation is that ARID1A loss can relax transcriptional and chromatin constraints in a manner that permits variability-associated components to emerge, while the genes contributing to those components and the extent of their coupling to chromatin-accessibility entropy depend on the pre-existing molecular context. Thus, reproducibility lies in the recurrence of a mode of diversification in selected backgrounds, not in the generation of a single, fixed transcriptional state.

Table S1.
List of genes constituting the GEP identified by 28 clone analysis (BxPC3 cells).

Table S2.
Genes nearest to C3/C4-specific accessible peaks overlapping ENCODE cCREs

Table S3.
List of genes constituting the GEP (BxPC3_2, HPAC, SUIT-2, SW1990 cells)

Table S4.
List of genes constituting the GEP (InVitro, InVivo)

Table S5.
List of genes constituting the GEP identified by 2,000 clone analysis (Fig. 5).

Table S6.
Sequence of shPseuMO-Tag and PseuMO-Tag.

Table S7.
List of primers used in the manuscript.

Table S6.
Sequence of shPseuMO-Tag and PseuMO-Tag.

1. shPseuMO-Tag-shS (8,720 bp, circular)

```
LOCUS       shPseuMO-Tag-shS        8720 bp    DNA     circular SYN 07-AUG-2025
DEFINITION  Lentiviral shPseuMO-Tag vector encoding U6-driven shScram#2 together
            with a CMV-driven mCherry-P2A-PuroR cassette and a
            Tn5/Nextera-flanked cell barcode module.
KEYWORDS    .
SOURCE      synthetic DNA construct
  ORGANISM  synthetic DNA construct
FEATURES             Location/Qualifiers
     source          1..8720
                     /mol_type="other DNA"
                     /organism="synthetic DNA construct"
     rep_origin      1..589
                     /direction=RIGHT
                     /label="ori"
                     /note="high-copy-number ColE1/pMB1/pBR322/pUC origin of
                     replication"
     protein_bind    877..898
                     /label="CAP binding site"
                     /bound_moiety="E. coli catabolite activator protein"
                     /note="CAP binding activates transcription in the presence
                     of cAMP."
     promoter        join(913..918,919..936,937..943)
                     /label="lac promoter"
                     /note="promoter for the E. coli lac operon"
     protein_bind    951..967
                     /label="lac operator"
                     /bound_moiety="lac repressor encoded by lacI"
                     /note="The lac repressor binds to the lac operator to
                     inhibit transcription in E. coli. This inhibition can be
                     relieved by adding lactose or
                     isopropyl-beta-D-thiogalactopyranoside (IPTG)."
     primer_bind     975..991
                     /label="M13 rev"
                     /note="common sequencing primer, one of multiple similar
                     variants"
     promoter        1012..1030
                     /label="T3 promoter"
                     /note="promoter for bacteriophage T3 RNA polymerase"
     promoter        1058..1284
                     /label="RSV promoter"
                     /note="Rous sarcoma virus enhancer/promoter"
     LTR             1285..1465
                     /label="5' LTR (truncated)"
                     /note="truncated 5' long terminal repeat (LTR) from HIV-1"
     misc_feature    1512..1637
                     /label="HIV-1 Psi"
                     /note="packaging signal of human immunodeficiency virus
                     type 1"
     misc_feature    2130..2363
                     /label="RRE"
                     /note="The Rev response element (RRE) of HIV-1 allows for
                     Rev-dependent mRNA export from the nucleus to the
                     cytoplasm."
     CDS             2548..2592
                     /codon_start=1
                     /product="antigenic peptide corresponding to amino acids
                     655 to 669 of the HIV envelope protein gp41 (Lutje Hulsik
                     et al., 2013)"
                     /label="gp41 peptide"
                     /note="recognized by the 2H10 single-chain llama nanobody"
                     /translation="KNEQELLELDKWASL"
     promoter        2886..3126
                     /label="U6 promoter"
                     /note="RNA polymerase III promoter for human U6 snRNA"
```

```
     misc_feature    3136..3188
                     /label="shScram#2"
     misc_feature    3230..3347
                     /label="cPPT/CTS"
                     /note="central polypurine tract and central termination
                     sequence of HIV-1"
     enhancer        3431..3734
                     /label="CMV enhancer"
                     /note="human cytomegalovirus immediate early enhancer"
     promoter        3735..3938
                     /label="CMV promoter"
                     /note="human cytomegalovirus (CMV) immediate early
                     promoter"
     regulatory      3945..3954
                     /label="Kozak sequence"
                     /note="vertebrate consensus sequence for strong initiation
                     of translation (Kozak, 1987)"
                     /regulatory_class="other"
     CDS             3951..4658
                     /codon_start=1
                     /product="monomeric derivative of DsRed fluorescent protein
                     (Shaner et al., 2004)"
                     /label="mCherry"
                     /note="mammalian codon-optimized"
                     /translation="MVSKGEEDNMAIIKEFMRFKVHMEGSVNGHEFEIEGEGEGRPYEG
                     TQTAKLKVTKGGPLPFAWDILSPQFMYGSKAYVKHPADIPDYLKLSFPEGFKWERVMNF
                     EDGGVVTVTQDSSLQDGEFIYKVKLRGTNFPSDGPVMQKKTMGWEASSERMYPEDGALK
                     GEIKQRLKLKDGGHYDAEVKTTYKAKKPVQLPGAYNVNIKLDITSHNEDYTIVEQYERA
                     EGRHSTGGMDELYK"
     CDS             4668..4724
                     /codon_start=1
                     /product="2A peptide from porcine teschovirus-1
                     polyprotein"
                     /label="P2A"
                     /note="Eukaryotic ribosomes fail to insert a peptide bond
                     between the Gly and Pro residues, yielding separate
                     polypeptides."
                     /note="cleavage site after base 4721"
                     /translation="ATNFSLLKQAGDVEENPGP"
     CDS             4725..5321
                     /codon_start=1
                     /gene="pac from Streptomyces alboniger"
                     /product="puromycin N-acetyltransferase"
                     /label="PuroR"
                     /note="confers resistance to puromycin"
                     /translation="TEYKPTVRLATRDDVPRAVRTLAAAFADYPATRHTVDPDRHIERV
                     TELQELFLTRVGLDIGKVWVADDGAAVAVWTTPESVEAGAVFAEIGPRMAELSGSRLAA
                     QQQMEGLLAPHRPKEPAWFLATVGVSPDHQGKGLGSAVVLPGVEAAERAGVPAFLETSA
                     PRNLPFYERLGFTVTADVEVPEGPRTWCMTRKPGA"
     misc_feature    5322..5335
                     /label="Nextera S5xx primer entry point (s5)"
     misc_feature    5336..5354
                     /label="Tn5 ME S5"
                     /note="hyperactive mosaic end for Tn5 transposase
                     recognition (Reznikoff et al., 2004)"
     misc_feature    5355..5390
                     /label="PCR Handle_Fwd"
     gap             5391..5395
                     /label="HashTag"
                     /estimated_length=5
     gap             5396..5405
                     /label="Cell BCD"
                     /estimated_length=10
     gap             5406..5410
                     /label="HashTag"
                     /estimated_length=5
     misc_feature    complement(5411..5435)
                     /label="PCR Handle_Rev"
     misc_feature    5436..5454
                     /label="Tn5 ME N7"
```

```
                     /note="hyperactive mosaic end for Tn5 transposase
                     recognition (Reznikoff et al., 2004)"
     misc_feature    complement(5455..5469)
                     /label="Nextera N7xx primer entry point (s7)"
     polyA_signal    5471..5605
                     /label="SV40 poly(A) signal"
                     /note="SV40 polyadenylation signal"
     misc_feature    5633..6221
                     /label="WPRE"
                     /note="woodchuck hepatitis virus posttranscriptional
                     regulatory element"
     CDS             complement(6104..6115)
                     /codon_start=1
                     /product="Factor Xa recognition and cleavage site"
                     /label="Factor Xa site"
                     /note="cleavage site after base 6103"
                     /translation="IEGR"
     LTR             6296..6529
                     /label="3' LTR (Delta-U3)"
                     /note="self-inactivating 3' long terminal repeat (LTR) from
                     HIV-1"
     polyA_signal    6601..6722
                     /label="SV40 poly(A) signal"
                     /note="SV40 polyadenylation signal"
     rep_origin      6762..6897
                     /label="SV40 ori"
                     /note="SV40 origin of replication"
     promoter        complement(6918..6936)
                     /label="T7 promoter"
                     /note="promoter for bacteriophage T7 RNA polymerase"
     primer_bind     complement(6946..6962)
                     /label="M13 fwd"
                     /note="common sequencing primer, one of multiple similar
                     variants"
     rep_origin      7104..7559
                     /direction=RIGHT
                     /label="f1 ori"
                     /note="f1 bacteriophage origin of replication; arrow
                     indicates direction of (+) strand synthesis"
     promoter        7585..7689
                     /gene="bla"
                     /label="AmpR promoter"
     CDS             join(7690..7758,7759..8550)
                     /codon_start=1
                     /gene="bla"
                     /product="beta-lactamase"
                     /label="AmpR"
                     /note="confers resistance to ampicillin, carbenicillin, and
                     related antibiotics"
                     /note="signal sequence at 7690..7758; cleavage site after
                     base 7758"
                     /translation="MSIQHFRVALIPFFAAFCLPVFAHPETLVKVKDAEDQLGARVGYI
                     ELDLNSGKILESFRPEERFPMMSTFKVLLCGAVLSRIDAGQEQLGRRIHYSQNDLVEYS
                     PVTEKHLTDGMTVRELCSAAITMSDNTAANLLLTTIGGPKELTAFLHNMGDHVTRLDRW
                     EPELNEAIPNDERDTTMPVAMATTLRKLLTGELLTLASRQQLIDWMEADKVAGPLLRSA
                     LPAGWFIADKSGAGERGSRGIIAALGPDGKPSRIVVIYTTGSQATMDERNRQIAEIGAS
                     LIKHW"
ORIGIN
        1 ttgagatcct ttttttctgc gcgtaatctg ctgcttgcaa acaaaaaaac caccgctacc
       61 agcggtggtt tgtttgccgg atcaagagct accaactctt tttccgaagg taactggctt
      121 cagcagagcg cagataccaa atactgttct tctagtgtag ccgtagttag gccaccactt
      181 caagaactct gtagcaccgc ctacatacct cgctctgcta atcctgttac cagtggctgc
      241 tgccagtggc gataagtcgt gtcttaccgg gttggactca agacgatagt taccggataa
      301 ggcgcagcgg tcgggctgaa cggggggttc gtgcacacag cccagcttgg agcgaacgac
      361 ctacaccgaa ctgagatacc tacagcgtga gctatgagaa agcgccacgc ttcccgaagg
      421 gagaaaggcg gacaggtatc cggtaagcgg cagggtcgga acaggagagc gcacgaggga
      481 gcttccaggg ggaaacgcct ggtatcttta tagtcctgtc gggtttcgcc acctctgact
      541 tgagcgtcga tttttgtgat gctcgtcagg ggggcggagc ctatggaaaa acgccagcaa
      601 cgcggccttt ttacggttcc tggccttttg ctggcctttt gctcacatgt tctttcctgc
      661 gttatcccct gattctgtgg ataaccgtat taccgccttt gagtgagctg ataccgctcg
```

721 ccgcagccga acgaccgagc gcagcgagtc agtgagcgag gaagcggaag agcgcccaat
781 acgcaaaccg cctctccccg cgcgttggcc gattcattaa tgcagctggc acgacaggtt
841 tcccgactgg aaagcgggca gtgagcgcaa cgcaattaat gtgagttagc tcactcatta
901 ggcaccccag gctttacact ttatgcttcc ggctcgtatg ttgtgtggaa ttgtgagcgg
961 ataacaattt cacacaggaa acagctatga ccatgattac gccaagcgcg caattaaccc
1021 tcactaaagg gaacaaaagc tggagctgca agcttaatgt agtcttatgc aatactcttg
1081 tagtcttgca acatggtaac gatgagttag caacatgcct tacaaggaga gaaaaagcac
1141 cgtgcatgcc gattggtgga agtaaggtgg tacgatcgtg ccttattagg aaggcaacag
1201 acgggtctga catggattgg acgaaccact gaattgccgc attgcagaga tattgtattt
1261 aagtgcctag ctcgatacat aaacgggtct ctctggttag accagatctg agcctgggag
1321 ctctctggct aactagggaa cccactgctt aagcctcaat aaagcttgcc ttgagtgctt
1381 caagtagtgt gtgcccgtct gttgtgtgac tctggtaact agagatccct cagacccttt
1441 tagtcagtgt ggaaaatctc tagcagtggc gcccgaacag ggacttgaaa gcgaaaggga
1501 aaccagagga gctctctcga cgcaggactc ggcttgctga agcgcgcacg gcaagaggcg
1561 aggggcggcg actggtgagt acgccaaaaa ttttgactag cggaggctag aaggagagag
1621 atgggtgcga gagcgtcagt attaagcggg ggagaattag atcgcgatgg gaaaaaattc
1681 ggttaaggcc agggggaaag aaaaaatata aattaaaaca tatagtatgg gcaagcaggg
1741 agctagaacg attcgcagtt aatcctggcc tgttagaaac atcagaaggc tgtagacaaa
1801 tactgggaca gctacaacca tcccttcaga caggatcaga agaacttaga tcattatata
1861 atacagtagc aaccctctat tgtgtgcatc aaaggataga gataaaagac accaaggaag
1921 ctttagacaa gatagaggaa gagcaaaaca aaagtaagac caccgcacag caagcggccg
1981 ctgatcttca gacctggagg aggagatatg agggacaatt ggagaagtga attatataaa
2041 tataaagtag taaaaattga accattagga gtagcaccca ccaaggcaaa gagaagagtg
2101 gtgcagagag aaaaaagagc agtgggaata ggagctttgt tccttgggtt cttgggagca
2161 gcaggaagca ctatgggcgc agcgtcaatg acgctgacgg tacaggccag acaattattg
2221 tctggtatag tgcagcagca gaacaatttg ctgagggcta ttgaggcgca acagcatctg
2281 ttgcaactca cagtctgggg catcaagcag ctccaggcaa gaatcctggc tgtggaaaga
2341 tacctaaagg atcaacagct cctggggatt tggggttgct ctggaaaact catttgcacc
2401 actgctgtgc cttggaatgc tagttggagt aataaatctc tggaacagat ttggaatcac
2461 acgacctgga tggagtggga cagagaaatt aacaattaca caagcttaat acactcctta
2521 attgaagaat cgcaaaacca gcaagaaaag aatgaacaag aattattgga attagataaa
2581 tgggcaagtt tgtggaattg gtttaacata acaaattggc tgtggtatat aaaattattc
2641 ataatgatag taggaggctt ggtaggttta agaatagttt ttgctgtact ttctatagtg
2701 aatagagtta ggcagggata ttcaccatta tcgtttcaga cccacctccc aaccccgagg
2761 ggacccgaca ggcccgaagg aatagaagaa gaaggtggag agagagacag agacagatcc
2821 attcgattag tgaacggatc tcgacggtat cggttaacta gcctcgagcg gccgccccct
2881 tcaccgaggg cctatttccc atgattcctt catatttgca tatacgatac aaggctgtta
2941 gagagataat tggaattaat ttgactgtaa acacaaagat attagtacaa aatacgtgac
3001 gtagaaagta ataatttctt gggtagtttg cagttttaaa attatgtttt aaaatggact
3061 atcatatgct taccgtaact tgaaagtatt tcgatttctt ggctttatat atcttgtgga
3121 aaggacgcgg gatccagtct taatcgcgta taagttcaag agaagtctta atcgcgtata
3181 agtttttttgt cgactcgacc tcgagacaaa tggcagtatt catccacaat tttaaaagaa
3241 aaggggggat tggggggtac agtgcagggg aaagaatagt agacataata gcaacagaca
3301 tacaaactaa agaattacaa aaacaaatta caaaaattca aaattttcgg gtttattaca
3361 gggacagcag agatccactt tggccgcggc tcgagtcccc aggctcccca tctagagctt
3421 gggagttccg cgttacataa cttacggtaa atggcccgcc tggctgaccg cccaacgacc
3481 cccgcccatt gacgtcaata atgacgtatg ttcccatagt aacgccaata gggactttcc
3541 attgacgtca atgggtggag tatttacggt aaactgccca cttggcagta catcaagtgt
3601 atcatatgcc aagtacgccc cctattgacg tcaatgacgg taaatggccc gcctggcatt
3661 atgcccagta catgacctta tgggactttc ctacttggca gtacatctac gtattagtca
3721 tcgctattac catggtgatg cggttttggc agtacatcaa tgggcgtgga tagcggtttg
3781 actcacgggg atttccaagt ctccacccca ttgacgtcaa tgggagtttg ttttggcacc
3841 aaaatcaacg ggactttcca aaatgtcgta acaactccgc cccattgacg caaatgggcg
3901 gtaggcgtgt acggtgggag gtctatataa gcagagctaa gcttgccacc atggtgagca
3961 agggcgagga ggataacatg gccatcatca aggagttcat gcgcttcaag gtgcacatgg
4021 agggctccgt gaacggccac gagttcgaga tcgagggcga gggcgagggc cgcccctacg
4081 agggcaccca gaccgccaag ctgaaggtga ccaagggtgg cccccctgccc ttcgcctggg
4141 acatcctgtc ccctcagttc atgtacggct ccaaggccta cgtgaagcac cccgccgaca
4201 tccccgacta cttgaagctg tccttccccg agggcttcaa gtgggagcgc gtgatgaact
4261 tcgaggacgg cggcgtggtg accgtgaccc aggactcctc cctgcaggac ggcgagttca
4321 tctacaaggt gaagctgcgc ggcaccaact tcccctccga cggccccgta atgcagaaga
4381 agaccatggg ctgggaggcc tcctccgagc ggatgtaccc cgaggacggc gccctgaagg
4441 gcgagatcaa gcagaggctg aagctgaagg acggcggcca ctacgacgct gaggtcaaga
4501 ccacctacaa ggccaagaag cccgtgcagc tgcccggcgc ctacaacgtc aacatcaagt
4561 tggacatcac ctcccacaac gaggactaca ccatcgtgga acagtacgaa cgcgccgagg
4621 gccgccactc caccggcggc atggacgagc tgtacaaggg ctccggcgca acaaacttct
4681 ctctgctgaa acaagccgga gatgtcgaag agaatcctgg accgaccgag tacaagccca
4741 cggtgcgcct cgccacccgc gacgacgtcc ccagggccgt acgcaccctc gccgccgcgt
4801 tcgccgacta ccccgccacg cgccacaccg tcgatccgga ccgccacatc gagcgggtca
4861 ccgagctgca agaactcttc ctcacgcgcg tcgggctcga catcggcaag gtgtgggtcg

4921 cggacgacgg cgccgcggtg gcggtctgga ccacgccgga gagcgtcgaa gcgggggcgg
4981 tgttcgccga gatcggcccg cgcatggccg agttgagcgg ttcccggctg gccgcgcagc
5041 aacagatgga aggcctcctg gcgccgcacc ggcccaagga gcccgcgtgg ttcctggcca
5101 ccgtcggagt ctcgcccgac caccagggca agggtctggg cagcgccgtc gtgctccccg
5161 gagtggaggc ggccgagcgc gccggggtgc ccgccttcct ggagacctcc gcgccccgca
5221 acctcccctt ctacgagcgg ctcggcttca ccgtcaccgc cgacgtcgag gtgcccgaag
5281 gaccgcgcac ctggtgcatg acccgcaagc ccggtgcctg atcgtcggca gcgtcagatg
5341 tgtataagag acagtagaag gcacagtcga ggcaggaaac agctatgact atgcannnnn
5401 nnnnntgcat cgttgagcaa taactagcga gcggactgtc tcttatacac atctccgagc
5461 ccacgagaca gatccagaca tgataagata cattgatgag tttggacaaa ccacaactag
5521 aatgcagtga aaaaaatgct ttatttgtga aatttgtgat gctattgctt tatttgtaac
5581 cattataagc tgcaataaac aagtttctga ggcttgggcc cagttcctcg acaatcaacc
5641 tctggattac aaaatttgtg aaagattgac tggtattctt aactatgttg ctccttttac
5701 gctatgtgga tacgctgctt taatgccttt gtatcatgct attgcttccc gtatggcttt
5761 cattttctcc tccttgtata aatcctggtt gctgtctctt tatgaggagt tgtggcccgt
5821 tgtcaggcaa cgtggcgtgg tgtgcactgt gtttgctgac gcaaccccca ctggttgggg
5881 cattgccacc acctgtcagc tcctttccgg gactttcgct ttccccctcc ctattgccac
5941 ggcggaactc atcgccgcct gccttgcccg ctgctggaca ggggctcggc tgttgggcac
6001 tgacaattcc gtggtgttgt cggggaagct gacgtccttt ccatggctgc tcgcctgtgt
6061 tgccacctgg attctgcgcg ggacgtcctt ctgctacgtc ccttcggccc tcaatccagc
6121 ggaccttcct tcccgcggcc tgctgccggc tctgcggcct cttccgcgtc ttcgccttcg
6181 ccctcagacg agtcggatct ccctttgggc cgcctccccg cctggaattc tttaagacca
6241 atgacttaca aggcagctgt agatcttagc cactttttaa aagaaaaggg gggactggaa
6301 gggctagctc actcccaacg aagacaagat ctgctttttg cttgtactgg gtctctctgg
6361 ttagaccaga tctgagcctg ggagctctct ggctaactag ggaacccact gcttaagcct
6421 caataaagct tgccttgagt gcttcaagta gtgtgtgccc gtctgttgtg tgactctggt
6481 aactagagat ccctcagacc cttttagtca gtgtggaaaa tctctagcag tagtagttca
6541 tgtcatctta ttattcagta tttataactt gcaaagaaat gaatatcaga gagtgagagg
6601 aacttgttta ttgcagctta taatggttac aaataaagca atagcatcac aaatttcaca
6661 aataaagcat ttttttcact gcattctagt tgtggtttgt ccaaactcat caatgtatct
6721 tatcatgtct ggctctagct atcccgcccc taactccgcc catcccgccc ctaactccgc
6781 ccagttccgc ccattctccg ccccatggct gactaatttt ttttatttat gcagaggccg
6841 aggccgcctc ggcctctgag ctattccaga agtagtgagg aggctttttt ggaggcctag
6901 ggacgtaccc aattcgccct atagtgagtc gtattacgcg cgctcactgg ccgtcgtttt
6961 acaacgtcgt gactgggaaa accctggcgt tacccaactt aatcgccttg cagcacatcc
7021 ccctttcgcc agctggcgta atagcgaaga ggcccgcacc gatcgccctt cccaacagtt
7081 gcgcagcctg aatggcgaat gggacgcgcc ctgtagcggc gcattaagcg cggcgggtgt
7141 ggtggttacg cgcagcgtga ccgctacact tgccagcgcc ctagcgcccg ctcctttcgc
7201 tttcttccct tcctttctcg ccacgttcgc cggctttccc cgtcaagctc taaatcgggg
7261 gctcccttta gggttccgat ttagtgcttt acggcacctc gaccccaaaa aacttgatta
7321 gggtgatggt tcacgtagtg ggccatcgcc ctgatagacg gtttttcgcc ctttgacgtt
7381 ggagtccacg ttctttaata gtggactctt gttccaaact ggaacaacac tcaaccctat
7441 ctcggtctat tcttttgatt tataagggat tttgccgatt tcggcctatt ggttaaaaaa
7501 tgagctgatt taacaaaaat ttaacgcgaa ttttaacaaa atattaacgc ttacaattta
7561 ggtggcactt ttcggggaaa tgtgcgcgga acccctattt gtttattttt ctaaatacat
7621 tcaaatatgt atccgctcat gagacaataa ccctgataaa tgcttcaata atattgaaaa
7681 aggaagagta tgagtattca acatttccgt gtcgccctta ttcccttttt tgcggcattt
7741 tgccttcctg tttttgctca cccagaaacg ctggtgaaag taaaagatgc tgaagatcag
7801 ttgggtgcac gagtgggtta catcgaactg gatctcaaca gcggtaagat ccttgagagt
7861 tttcgccccg aagaacgttt tccaatgatg agcactttta aagttctgct atgtggcgcg
7921 gtattatccc gtattgacgc cgggcaagag caactcggtc gccgcataca ctattctcag
7981 aatgacttgg ttgagtactc accagtcaca gaaaagcatc ttacggatgg catgacagta
8041 agagaattat gcagtgctgc cataaccatg agtgataaca ctgcggccaa cttacttctg
8101 acaacgatcg gaggaccgaa ggagctaacc gcttttttgc acaacatggg ggatcatgta
8161 actcgccttg atcgttggga accggagctg aatgaagcca taccaaacga cgagcgtgac
8221 accacgatgc ctgtagcaat ggcaacaacg ttgcgcaaac tattaactgg cgaactactt
8281 actctagctt cccggcaaca attaatagac tggatggagg cggataaagt tgcaggacca
8341 cttctgcgct cggcccttcc ggctggctgg tttattgctg ataaatctgg agccggtgag
8401 cgtgggtctc gcggtatcat tgcagcactg gggccagatg gtaagccctc ccgtatcgta
8461 gttatctaca cgacggggag tcaggcaact atggatgaac gaaatagaca gatcgctgag
8521 ataggtgcct cactgattaa gcattggtaa ctgtcagacc aagtttactc atatatactt
8581 tagattgatt taaaacttca tttttaattt aaaaggatct aggtgaagat cctttttgat
8641 aatctcatga ccaaaatccc ttaacgtgag ttttcgttcc actgagcgtc agaccccgta
8701 gaaaagatca aaggatcttc
//

2. PseuMO-Tag (8,404 bp, circular)

```
LOCUS       PseuMO-Tag              8404 bp    DNA     circular SYN 07-AUG-2025
DEFINITION  Lentiviral PseuMO-Tag vector encoding a CMV-driven mCherry-P2A-PuroR
            cassette and a Tn5/Nextera-flanked PseuTag clonal barcode module.
KEYWORDS    .
SOURCE      synthetic DNA construct
  ORGANISM  synthetic DNA construct
FEATURES             Location/Qualifiers
     source          1..8404
                     /mol_type="other DNA"
                     /organism="synthetic DNA construct"
     promoter        1..105
                     /gene="bla"
                     /label="AmpR promoter"
     CDS             join(106..174,175..966)
                     /codon_start=1
                     /gene="bla"
                     /product="beta-lactamase"
                     /label="AmpR"
                     /note="confers resistance to ampicillin, carbenicillin, and
                     related antibiotics"
                     /note="signal sequence at 106..174; cleavage site after
                     base 174"
                     /translation="MSIQHFRVALIPFFAAFCLPVFAHPETLVKVKDAEDQLGARVGYI
                     ELDLNSGKILESFRPEERFPMMSTFKVLLCGAVLSRIDAGQEQLGRRIHYSQNDLVEYS
                     PVTEKHLTDGMTVRELCSAAITMSDNTAANLLLTTIGGPKELTAFLHNMGDHVTRLDRW
                     EPELNEAIPNDERDTTMPVAMATTLRKLLTGELLTLASRQQLIDWMEADKVAGPLLRSA
                     LPAGWFIADKSGAGERGSRGIIAALGPDGKPSRIVVIYTTGSQATMDERNRQIAEIGAS
                     LIKHW"
     rep_origin      1137..1725
                     /direction=RIGHT
                     /label="ori"
                     /note="high-copy-number ColE1/pMB1/pBR322/pUC origin of
                     replication"
     protein_bind    2013..2034
                     /label="CAP binding site"
                     /bound_moiety="E. coli catabolite activator protein"
                     /note="CAP binding activates transcription in the presence
                     of cAMP."
     promoter        join(2049..2054,2055..2072,2073..2079)
                     /label="lac promoter"
                     /note="promoter for the E. coli lac operon"
     protein_bind    2087..2103
                     /label="lac operator"
                     /bound_moiety="lac repressor encoded by lacI"
                     /note="The lac repressor binds to the lac operator to
                     inhibit transcription in E. coli. This inhibition can be
                     relieved by adding lactose or
                     isopropyl-beta-D-thiogalactopyranoside (IPTG)."
     primer_bind     2111..2127
                     /label="M13 rev"
                     /note="common sequencing primer, one of multiple similar
                     variants"
     promoter        2148..2166
                     /label="T3 promoter"
                     /note="promoter for bacteriophage T3 RNA polymerase"
     promoter        2194..2420
                     /label="RSV promoter"
                     /note="Rous sarcoma virus enhancer/promoter"
     LTR             2421..2601
                     /label="5' LTR (truncated)"
                     /note="truncated 5' long terminal repeat (LTR) from HIV-1"
     misc_feature    2648..2773
                     /label="HIV-1 Psi"
                     /note="packaging signal of human immunodeficiency virus
                     type 1"
     misc_feature    3266..3499
                     /label="RRE"
                     /note="The Rev response element (RRE) of HIV-1 allows for
```

```
                     Rev-dependent mRNA export from the nucleus to the
                     cytoplasm."
     CDS             3684..3728
                     /codon_start=1
                     /product="antigenic peptide corresponding to amino acids
                     655 to 669 of the HIV envelope protein gp41 (Lutje Hulsik
                     et al., 2013)"
                     /label="gp41 peptide"
                     /note="recognized by the 2H10 single-chain llama nanobody"
                     /translation="KNEQELLELDKWASL"
     misc_feature    4032..4149
                     /label="cPPT/CTS"
                     /note="central polypurine tract and central termination
                     sequence of HIV-1"
     enhancer        4233..4536
                     /label="CMV enhancer"
                     /note="human cytomegalovirus immediate early enhancer"
     promoter        4537..4740
                     /label="CMV promoter"
                     /note="human cytomegalovirus (CMV) immediate early
                     promoter"
     misc_feature    4747..4756
                     /label="Kozak sequence"
     CDS             4753..5460
                     /codon_start=1
                     /product="monomeric derivative of DsRed fluorescent protein
                     (Shaner et al., 2004)"
                     /label="mCherry"
                     /note="mammalian codon-optimized"
                     /translation="MVSKGEEDNMAIIKEFMRFKVHMEGSVNGHEFEIEGEGEGRPYEG
                     TQTAKLKVTKGGPLPFAWDILSPQFMYGSKAYVKHPADIPDYLKLSFPEGFKWERVMNF
                     EDGGVVTVTQDSSLQDGEFIYKVKLRGTNFPSDGPVMQKKTMGWEASSERMYPEDGALK
                     GEIKQRLKLKDGGHYDAEVKTTYKAKKPVQLPGAYNVNIKLDITSHNEDYTIVEQYERA
                     EGRHSTGGMDELYK"
     CDS             5470..5526
                     /codon_start=1
                     /product="2A peptide from porcine teschovirus-1
                     polyprotein"
                     /label="P2A"
                     /note="Eukaryotic ribosomes fail to insert a peptide bond
                     between the Gly and Pro residues, yielding separate
                     polypeptides."
                     /note="cleavage site after base 5523"
                     /translation="ATNFSLLKQAGDVEENPGP"
     CDS             5527..6123
                     /codon_start=1
                     /gene="pac from Streptomyces alboniger"
                     /product="puromycin N-acetyltransferase"
                     /label="PuroR"
                     /note="confers resistance to puromycin"
                     /translation="TEYKPTVRLATRDDVPRAVRTLAAAFADYPATRHTVDPDRHIERV
                     TELQELFLTRVGLDIGKVWVADDGAAVAVWTTPESVEAGAVFAEIGPRMAELSGSRLAA
                     QQQMEGLLAPHRPKEPAWFLATVGVSPDHQGKGLGSAVVLPGVEAAERAGVPAFLETSA
                     PRNLPFYERLGFTVTADVEVPEGPRTWCMTRKPGA"
     misc_feature    6124..6137
                     /label="Nextera S5xx primer entry point (s5)"
     misc_feature    6138..6156
                     /label="Tn5 ME S5"
                     /note="hyperactive mosaic end for Tn5 transposase
                     recognition (Reznikoff et al., 2004)"
     misc_feature    6157..6187
                     /label="PCR Handle1"
     misc_feature    6188..6191
                     /label="BsmBI"
     gap             6193..6197
                     /label="HashTag"
                     /estimated_length=5
     misc_feature    6198..6225
                     /label="PseuTag"
     gap             6226..6230
```

```
                     /label="HashTag"
                     /estimated_length=5
     misc_feature    6232..6255
                     /label="PCR Handle2"
     misc_feature    6256..6274
                     /label="Tn5 ME N7"
                     /note="hyperactive mosaic end for Tn5 transposase
                     recognition (Reznikoff et al., 2004)"
     misc_feature    complement(6275..6289)
                     /label="Nextera N7xx primer entry point (s7)"
     polyA_signal    6291..6425
                     /label="SV40 poly(A) signal"
                     /note="SV40 polyadenylation signal"
     misc_feature    6453..7041
                     /label="WPRE"
                     /note="woodchuck hepatitis virus posttranscriptional
                     regulatory element"
     CDS             complement(6924..6935)
                     /codon_start=1
                     /product="Factor Xa recognition and cleavage site"
                     /label="Factor Xa site"
                     /note="cleavage site after base 6923"
                     /translation="IEGR"
     misc_feature    7045..7049
                     /label="EcoRI'"
     LTR             7116..7349
                     /label="3' LTR (Delta-U3)"
                     /note="self-inactivating 3' long terminal repeat (LTR) from
                     HIV-1"
     polyA_signal    7421..7542
                     /label="SV40 poly(A) signal"
                     /note="SV40 polyadenylation signal"
     rep_origin      7582..7717
                     /label="SV40 ori"
                     /note="SV40 origin of replication"
     promoter        complement(7738..7756)
                     /label="T7 promoter"
                     /note="promoter for bacteriophage T7 RNA polymerase"
     primer_bind     complement(7766..7782)
                     /label="M13 fwd"
                     /note="common sequencing primer, one of multiple similar
                     variants"
     rep_origin      7924..8379
                     /direction=RIGHT
                     /label="f1 ori"
                     /note="f1 bacteriophage origin of replication; arrow
                     indicates direction of (+) strand synthesis"
ORIGIN
        1 cgcggaaccc ctatttgttt atttttctaa atacattcaa atatgtatcc gctcatgaga
       61 caataaccct gataaatgct tcaataatat tgaaaaagga agagtatgag tattcaacat
      121 ttccgtgtcg cccttattcc cttttttgcg gcattttgcc ttcctgtttt tgctcaccca
      181 gaaacgctgg tgaaagtaaa agatgctgaa gatcagttgg gtgcacgagt gggttacatc
      241 gaactggatc tcaacagcgg taagatcctt gagagttttc gccccgaaga acgttttcca
      301 atgatgagca cttttaaagt tctgctatgt ggcgcggtat tatcccgtat tgacgccggg
      361 caagagcaac tcggtcgccg catacactat tctcagaatg acttggttga gtactcacca
      421 gtcacagaaa agcatcttac ggatggcatg acagtaagag aattatgcag tgctgccata
      481 accatgagtg ataacactgc ggccaactta cttctgacaa cgatcggagg accgaaggag
      541 ctaaccgctt ttttgcacaa catgggggat catgtaactc gccttgatcg ttgggaaccg
      601 gagctgaatg aagccatacc aaacgacgag cgtgacacca cgatgcctgt agcaatggca
      661 acaacgttgc gcaaactatt aactggcgaa ctacttactc tagcttcccg gcaacaatta
      721 atagactgga tggaggcgga taaagttgca ggaccacttc tgcgctcggc ccttccggct
      781 ggctggttta ttgctgataa atctggagcc ggtgagcgtg ggtctcgcgg tatcattgca
      841 gcactggggc cagatggtaa gccctcccgt atcgtagtta tctacacgac ggggagtcag
      901 gcaactatgg atgaacgaaa tagacagatc gctgagatag gtgcctcact gattaagcat
      961 tggtaactgt cagaccaagt ttactcatat atactttaga ttgatttaaa acttcatttt
     1021 taatttaaaa ggatctaggt gaagatcctt tttgataatc tcatgaccaa aatcccttaa
     1081 cgtgagtttt cgttccactg agcgtcagac cccgtagaaa agatcaaagg atcttcttga
     1141 gatccttttt ttctgcgcgt aatctgctgc ttgcaaacaa aaaaaccacc gctaccagcg
     1201 gtggtttgtt tgccggatca agagctacca actctttttc cgaaggtaac tggcttcagc
     1261 agagcgcaga taccaaatac tgttcttcta gtgtagccgt agttaggcca ccacttcaag
```

1321 aactctgtag caccgcctac atacctcgct ctgctaatcc tgttaccagt ggctgctgcc
1381 agtggcgata agtcgtgtct taccgggttg gactcaagac gatagttacc ggataaggcg
1441 cagcggtcgg gctgaacggg gggttcgtgc acacagccca gcttggagcg aacgacctac
1501 accgaactga gatacctaca gcgtgagcta tgagaaagcg ccacgcttcc cgaagggaga
1561 aaggcggaca ggtatccggt aagcggcagg gtcggaacag gagagcgcac gagggagctt
1621 ccagggggaa acgcctggta tctttatagt cctgtcgggt ttcgccacct ctgacttgag
1681 cgtcgatttt tgtgatgctc gtcagggggg cggagcctat ggaaaaacgc cagcaacgcg
1741 gcctttttac ggttcctggc cttttgctgg ccttttgctc acatgttctt tcctgcgtta
1801 tcccctgatt ctgtggataa ccgtattacc gcctttgagt gagctgatac cgctcgccgc
1861 agccgaacga ccgagcgcag cgagtcagtg agcgaggaag cggaagagcg cccaatacgc
1921 aaaccgcctc tccccgcgcg ttggccgatt cattaatgca gctggcacga caggtttccc
1981 gactggaaag cgggcagtga gcgcaacgca attaatgtga gttagctcac tcattaggca
2041 ccccaggctt tacactttat gcttccggct cgtatgttgt gtggaattgt gagcggataa
2101 caatttcaca caggaaacag ctatgaccat gattacgcca agcgcgcaat taaccctcac
2161 taaagggaac aaaagctgga gctgcaagct taatgtagtc ttatgcaata ctcttgtagt
2221 cttgcaacat ggtaacgatg agttagcaac atgccttaca aggagagaaa aagcaccgtg
2281 catgccgatt ggtggaagta aggtggtacg atcgtgcctt attaggaagg caacagacgg
2341 gtctgacatg gattggacga accactgaat tgccgcattg cagagatatt gtatttaagt
2401 gcctagctcg atacataaac gggtctctct ggttagacca gatctgagcc tgggagctct
2461 ctggctaact agggaaccca ctgcttaagc ctcaataaag cttgccttga gtgcttcaag
2521 tagtgtgtgc ccgtctgttg tgtgactctg gtaactagag atccctcaga cccttttagt
2581 cagtgtggaa aatctctagc agtggcgccc gaacagggac ttgaaagcga aagggaaacc
2641 agaggagctc tctcgacgca ggactcggct tgctgaagcg cgcacggcaa gaggcgaggg
2701 gcggcgactg gtgagtacgc caaaaatttt gactagcgga ggctagaagg agagagatgg
2761 gtgcgagagc gtcagtatta agcgggggag aattagatcg cgatgggaaa aaattcggtt
2821 aaggccaggg ggaaagaaaa aatataaatt aaaacatata gtatgggcaa gcagggagct
2881 agaacgattc gcagttaatc ctggcctgtt agaaacatca gaaggctgta gacaaatact
2941 gggacagcta caaccatccc ttcagacagg atcagaagaa cttagatcat tatataatac
3001 agtagcaacc ctctattgtg tgcatcaaag gatagagata aaagacacca aggaagcttt
3061 agacaagata gaggaagagc aaaacaaaag taagaccacc gcacagcaag cggccgctga
3121 tcttcagacc tggaggagga gatatgaggg acaattggag aagtgaatta tataaatata
3181 aagtagtaaa aattgaacca ttaggagtag cacccaccaa ggcaaagaga agagtggtgc
3241 agagagaaaa aagagcagtg ggaataggag ctttgttcct tgggttcttg ggagcagcag
3301 gaagcactat gggcgcagcg tcaatgacgc tgacggtaca ggccagacaa ttattgtctg
3361 gtatagtgca gcagcagaac aatttgctga gggctattga ggcgcaacag catctgttgc
3421 aactcacagt ctggggcatc aagcagctcc aggcaagaat cctggctgtg gaaagatacc
3481 taaaggatca acagctcctg gggatttggg gttgctctgg aaaactcatt tgcaccactg
3541 ctgtgccttg gaatgctagt tggagtaata aatctctgga acagatttgg aatcacacga
3601 cctggatgga gtgggacaga gaaattaaca attacacaag cttaatacac tccttaattg
3661 aagaatcgca aaaccagcaa gaaaagaatg aacaagaatt attggaatta gataaatggg
3721 caagtttgtg gaattggttt aacataacaa attggctgtg gtatataaaa ttattcataa
3781 tgatagtagg aggcttggta ggtttaagaa tagtttttgc tgtactttct atagtgaata
3841 gagttaggca gggatattca ccattatcgt ttcagaccca cctcccaacc ccgaggggac
3901 ccgacaggcc cgaaggaata gaagaagaag gtggagagag agacagagac agatccattc
3961 gattagtgaa cggatctcga cggtatcggt ttcgactcga cctcgagaca aatggcagta
4021 ttcatccaca attttaaaag aaaagggggg attggggggt acagtgcagg ggaaagaata
4081 gtagacataa tagcaacaga catacaaact aaagaattac aaaaacaaat tacaaaaatt
4141 caaaattttc gggtttatta cagggacagc agagatccac tttggccgcg gctcgagtcc
4201 ccaggctccc catctagagc ttgggagttc cgcgttacat aacttacggt aaatggcccg
4261 cctggctgac cgcccaacga cccccgccca ttgacgtcaa taatgacgta tgttcccata
4321 gtaacgccaa tagggacttt ccattgacgt caatgggtgg agtatttacg gtaaactgcc
4381 cacttggcag tacatcaagt gtatcatatg ccaagtacgc cccctattga cgtcaatgac
4441 ggtaaatggc ccgcctggca ttatgcccag tacatgacct tatgggactt tcctacttgg
4501 cagtacatct acgtattagt catcgctatt accatggtga tgcggttttg gcagtacatc
4561 aatgggcgtg gatagcggtt tgactcacgg ggatttccaa gtctccaccc cattgacgtc
4621 aatgggagtt tgttttggca ccaaaatcaa cgggactttc caaaatgtcg taacaactcc
4681 gccccattga cgcaaatggg cggtaggcgt gtacggtggg aggtctatat aagcagagct
4741 aagcttgcca ccatggtgag caagggcgag gaggataaca tggccatcat caaggagttc
4801 atgcgcttca aggtgcacat ggagggctcc gtgaacggcc acgagttcga gatcgagggc
4861 gagggcgagg gccgccccta cgagggcacc cagaccgcca agctgaaggt gaccaagggt
4921 ggccccctgc ccttcgcctg ggacatcctg tcccctcagt tcatgtacgg ctccaaggcc
4981 tacgtgaagc accccgccga catccccgac tacttgaagc tgtccttccc cgagggcttc
5041 aagtgggagc gcgtgatgaa cttcgaggac ggcggcgtgg tgaccgtgac ccaggactcc
5101 tccctgcagg acggcgagtt catctacaag gtgaagctgc gcggcaccaa cttcccctcc
5161 gacggccccg taatgcagaa gaagaccatg ggctgggagg cctcctccga gcggatgtac
5221 cccgaggacg gcgccctgaa gggcgagatc aagcagaggc tgaagctgaa ggacggcggc
5281 cactacgacg ctgaggtcaa gaccacctac aaggccaaga agcccgtgca gctgcccggc
5341 gcctacaacg tcaacatcaa gttggacatc acctcccaca acgaggacta caccatcgtg
5401 gaacagtacg aacgcgccga gggccgccac tccaccggcg gcatggacga gctgtacaag
5461 ggctccggcg caacaaactt ctctctgctg aaacaagccg gagatgtcga agagaatcct

```
5521 ggaccgaccg agtacaagcc cacggtgcgc ctcgccaccc gcgacgacgt ccccagggcc
5581 gtacgcaccc tcgccgccgc gttcgccgac taccccgcca cgcgccacac cgtcgatccg
5641 gaccgccaca tcgagcgggt caccgagctg caagaactct tcctcacgcg cgtcgggctc
5701 gacatcggca aggtgtgggt cgcggacgac ggcgccgcgg tggcggtctg gaccacgccg
5761 gagagcgtcg aagcgggggc ggtgttcgcc gagatcggcc cgcgcatggc cgagttgagc
5821 ggttcccggc tggccgcgca gcaacagatg gaaggcctcc tggcgccgca ccggcccaag
5881 gagcccgcgt ggttcctggc caccgtcgga gtctcgcccg accaccaggg caagggtctg
5941 ggcagcgccg tcgtgctccc cggagtggag gcggccgagc gcgccggggt gcccgccttc
6001 ctggagacct ccgcgccccg caacctcccc ttctacgagc ggctcggctt caccgtcacc
6061 gccgacgtcg aggtgcccga aggaccgcgc acctggtgca tgacccgcaa gcccggtgcc
6121 tgatcgtcgg cagcgtcaga tgtgtataag agacagtaga aggcacagtc gaggcaggaa
6181 acagctatga ctatgcannn gtnnnctnnn agnnntgnnn cannntgcat cgttgagcaa
6241 taactagcga gcggactgtc tcttatacac atctccgagc ccacgagaca gatccagaca
6301 tgataagata cattgatgag tttggacaaa ccacaactag aatgcagtga aaaaaatgct
6361 ttatttgtga aatttgtgat gctattgctt tatttgtaac cattataagc tgcaataaac
6421 aagtttctga ggcttgggcc cagttcctcg acaatcaacc tctggattac aaaatttgtg
6481 aaagattgac tggtattctt aactatgttg ctccttttac gctatgtgga tacgctgctt
6541 taatgccttt gtatcatgct attgcttccc gtatggcttt cattttctcc tccttgtata
6601 aatcctggtt gctgtctctt tatgaggagt tgtggcccgt tgtcaggcaa cgtggcgtgg
6661 tgtgcactgt gtttgctgac gcaaccccca ctggttgggg cattgccacc acctgtcagc
6721 tcctttccgg gactttcgct ttccccctcc ctattgccac ggcggaactc atcgccgcct
6781 gccttgcccg ctgctggaca ggggctcggc tgttgggcac tgacaattcc gtggtgttgt
6841 cggggaagct gacgtccttt ccatggctgc tcgcctgtgt tgccacctgg attctgcgcg
6901 ggacgtcctt ctgctacgtc ccttcggccc tcaatccagc ggaccttcct tcccgcggcc
6961 tgctgccggc tctgcggcct cttccgcgtc ttcgccttcg ccctcagacg agtcggatct
7021 ccctttgggc cgcctccccg cctggaattc tttaagacca atgacttaca aggcagctgt
7081 agatcttagc cactttttaa aagaaaaggg gggactggaa gggctagctc actcccaacg
7141 aagacaagat ctgctttttg cttgtactgg gtctctctgg ttagaccaga tctgagcctg
7201 ggagctctct ggctaactag ggaacccact gcttaagcct caataaagct tgccttgagt
7261 gcttcaagta gtgtgtgccc gtctgttgtg tgactctggt aactagagat ccctcagacc
7321 cttttagtca gtgtggaaaa tctctagcag tagtagttca tgtcatctta ttattcagta
7381 tttataactt gcaaagaaat gaatatcaga gagtgagagg aacttgttta ttgcagctta
7441 taatggttac aaataaagca atagcatcac aaatttcaca aataaagcat ttttttcact
7501 gcattctagt tgtggtttgt ccaaactcat caatgtatct tatcatgtct ggctctagct
7561 atcccgcccc taactccgcc catcccgccc ctaactccgc ccagttccgc ccattctccg
7621 ccccatggct gactaatttt ttttatttat gcagaggccg aggccgcctc ggcctctgag
7681 ctattccaga agtagtgagg aggctttttt ggaggcctag ggacgtaccc aattcgccct
7741 atagtgagtc gtattacgcg cgctcactgg ccgtcgtttt acaacgtcgt gactgggaaa
7801 accctggcgt tacccaactt aatcgccttg cagcacatcc ccctttcgcc agctggcgta
7861 atagcgaaga ggcccgcacc gatcgccctt cccaacagtt gcgcagcctg aatggcgaat
7921 gggacgcgcc ctgtagcggc gcattaagcg cggcgggtgt ggtggttacg cgcagcgtga
7981 ccgctacact tgccagcgcc ctagcgcccg ctcctttcgc tttcttccct tcctttctcg
8041 ccacgttcgc cggctttccc cgtcaagctc taaatcgggg gctcccttta gggttccgat
8101 ttagtgcttt acggcacctc gaccccaaaa aacttgatta gggtgatggt tcacgtagtg
8161 ggccatcgcc ctgatagacg gtttttcgcc ctttgacgtt ggagtccacg ttctttaata
8221 gtggactctt gttccaaact ggaacaacac tcaaccctat ctcggtctat tcttttgatt
8281 tataagggat tttgccgatt tcggcctatt ggttaaaaaa tgagctgatt taacaaaaat
8341 ttaacgcgaa ttttaacaaa atattaacgc ttacaattta ggtggcactt ttcggggaaa
8401 tgtg
```
//
